# A Dynamic Microtubule Cytoskeleton Directs Medial Actomyosin Function during Tube Formation

**DOI:** 10.1016/j.devcel.2014.03.023

**Published:** 2014-06-09

**Authors:** Alexander J.R. Booth, Guy B. Blanchard, Richard J. Adams, Katja Röper

**Affiliations:** 1 MRC Laboratory of Molecular Biology, Cambridge Biomedical Campus, Francis Crick Avenue, Cambridge CB2 0QH, UK; 2 Department of Physiology, Development and Neuroscience, University of Cambridge, Downing Street, Cambridge CB2 3DY, UK

## Abstract

The cytoskeleton is a major determinant of cell-shape changes that drive the formation of complex tissues during development. Important roles for actomyosin during tissue morphogenesis have been identified, but the role of the microtubule cytoskeleton is less clear. Here, we show that during tubulogenesis of the salivary glands in the fly embryo, the microtubule cytoskeleton undergoes major rearrangements, including a 90° change in alignment relative to the apicobasal axis, loss of centrosomal attachment, and apical stabilization. Disruption of the microtubule cytoskeleton leads to failure of apical constriction in placodal cells fated to invaginate. We show that this failure is due to loss of an apical medial actomyosin network whose pulsatile behavior in wild-type embryos drives the apical constriction of the cells. The medial actomyosin network interacts with the minus ends of acentrosomal microtubule bundles through the cytolinker protein Shot, and disruption of Shot also impairs apical constriction.

## Highlights

• Large-scale rearrangement of microtubules accompanies early tube formation
• Loss of microtubules leads to loss of apical constriction during tube formation
• During tubulogenesis, apical constriction is driven by pulsatile medial actomyosin
• Microtubules and the cytolinker Shot stabilize the medial actomyosin

## Graphical Abstract

**Figure:**
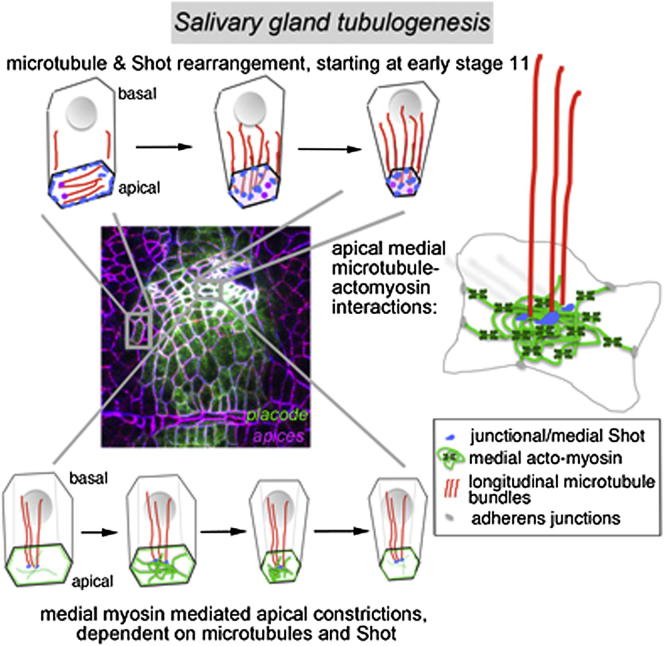

## Teaser

The cytoskeleton drives cell-shape changes during morphogenesis. Booth et al. shed light on how the dynamic apical medial actomyosin cytoskeleton depends on the presence of longitudinal microtubule bundles. These bundles interact with the actomyosin through the cytolinker protein Shot, and this interaction is important for tube morphogenesis in *Drosophila*.

## Introduction

Tissue formation during embryogenesis is largely driven by cell-shape changes and cell rearrangements. Cell shape itself is determined intracellularly by the cytoskeleton as well as by cell-extrinsic forces. Over the last decade, the importance of the actin cytoskeleton in the determination of cell shape has been shown for many tissues (Pollard and Cooper, 2009). Actin, with myosin, forms contractile arrays that are key constituents of different morphogenetic processes ranging from epithelial folding to cell intercalation and tissue convergence (Bertet et al., 2004; Martin et al., 2009; Simões et al., 2010). Important functions for different actomyosin structures have emerged, and a distinct population of apical medial actomyosin forming an interlinked network across many cell diameters may be crucial for apical cell constriction and the size of apical cell-cell junctions (Martin et al., 2009; Mason et al., 2013; Rauzi et al., 2010). However, relatively little is known of the roles of microtubules (MTs) during morphogenesis and cell-shape changes.

MTs serve as major tracks for cellular transport, including an important role in membrane uptake and delivery. They are also important for the turnover of adhesion receptors through endo- and exocytosis during cell growth and cell-shape changes (Akhmanova et al., 2009; Mimori-Kiyosue, 2011). However, whereas actin and actomyosin have roles in directly driving cell-shape changes, defined roles for MTs during these processes are scarcer. Examples of roles of MTs in *Drosophila* morphogenesis include roles in cell flattening during amnioserosa elongation (Pope and Harris, 2008), during the zippering stages of dorsal closure (Jankovics and Brunner, 2006), and in the establishment of the correct tracheal branching pattern in embryos (Brodu et al., 2010).

It remains to be elucidated how the actin and MT cytoskeletons interact during cell-shape changes and morphogenesis, even though we know that such crosstalk must be important (Bosher et al., 2003; Hetherington et al., 2011; Lee and Kolodziej, 2002; Röper and Brown, 2003). The clearest example for crosstalk is between aster MTs and the contractile actomyosin ring during cell division (D’Avino et al., 2008; Somers and Saint, 2003; Vale et al., 2009). Additionally, during cell migration and also growth cone steering, close interplay between actin and MTs is important (Basu and Chang, 2007; Broussard et al., 2008; Schaefer et al., 2008).

**Figure 1:**
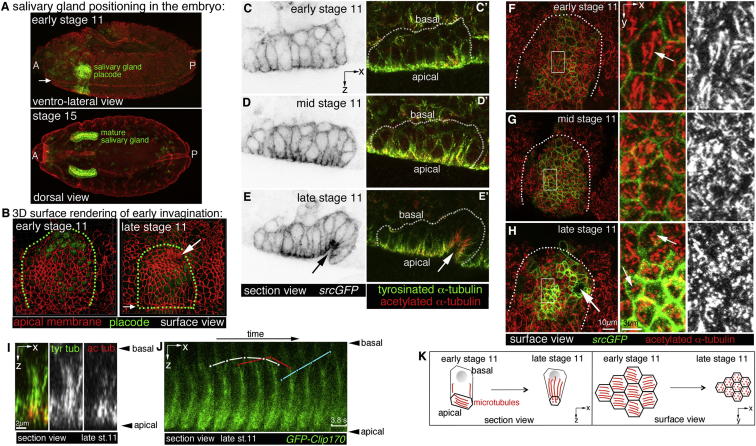
Microtubule Rearrangements during Early Salivary Gland Invagination (A) Confocal stacks illustrating the position of the salivary gland placode at early stage 11 (top panel; green) and of the fully invaginated glands at stage 15 (lower panel; green). A, anterior; P, posterior. (B) 3D rendering of apical cortices marked by Crumbs illustrates the early invagination of placodal cells (green), processing from a flat epithelial sheet at early stage 11 (left) to an early invaginated pit at late stage 11 (right). Small arrows in (A) and (B) point to the ventral midline; large arrow in (B) points to the forming pit; green dotted lines in (B) mark the placode area. (C–E′) Lateral section views of placodal cells at early stage 11 before apical constriction (C), midstage 11 during constriction but before invagination (D), and late stage 11 after initial invagination (E). *srcGFP* (under control of *fkhGal4*) is shown in inverse panels (C)–(E) to outline membranes of placodal cells. Labeling for tyrosinated α-tubulin (green in C′–E′) and acetylated α-tubulin (red in C′–E′) reveals that the number of MTs projecting from the apical surface into the cells increases during early constriction and invagination. The arrows in (E) and (E′) point to the invaginating pit. (F–H) Surface views of placodes showing that MTs undergo a 90° rearrangement during cell constriction. At early stage 11, labeling for acetylated α-tubulin (red) shows a dense MT network lying parallel to the apical surface of the cells (F). During midstage 11, these MT bundles change orientation (G) to run perpendicular to the apical surface as longitudinal bundles by late stage 11 (H). Constricting apices are marked by *srcGFP* in green. White boxes indicate areas magnified in the center and right panels; small arrows point to apical parallel MTs (F) and the end foci of longitudinal bundles (H); large arrow points to the invaginating pit; white dotted lines mark the placode area. (I) z section of an MT bundle, with the level of acetylation of MTs being greater nearer the apical surface. Acetylated α-tubulin, red; tyrosinated α-tubulin, green. (J) Kymograph of a z section time-lapse analysis of microtubule dynamics in an invaginating cell at late stage 11; MTs are visualized using *GFP-Clip170*; frames are 3.87 s apart (see Movie S1). White, blue, and red dots mark individual MTs emanating from the bundle. (K) Schematic of MT rearrangements in the placode during stage 11. See also Figure S1 and Movie S1.

We have used a model process of tube formation to address the role of the MT cytoskeleton during tissue morphogenesis. The tubes of the salivary gland in the *Drosophila* embryo form from two epithelial placodes through a process of highly coordinated apical cell constriction and invagination (Figure 1A) (Andrew and Ewald, 2010; Myat and Andrew, 2000). Once the cells of the placode have been specified, no further cell division or death occurs here, leaving cell-shape changes and rearrangements as the driving forces of the invagination and making this an ideal system to study how cell-shape changes and rearrangements drive tube invagination. Topologically similar processes of tube formation or budding in mammals can be found during early lung morphogenesis or the elaboration of kidney tubules (Costantini and Kopan, 2010; Warburton et al., 2010). We have previously shown that actomyosin plays an important role during morphogenesis of the glands and identified specific subpools of actomyosin present in the gland placode, in particular a dense junctional and apical medial actomyosin network as well as a circumferential actomyosin cable (Röper, 2012).

We demonstrate that during early steps of tube formation, the placodal MT cytoskeleton undergoes a radical 90° rearrangement with respect to the apicobasal axis, leading to a network of acentrosomal, longitudinal (parallel to the apicobasal axis) MT bundles that abut the apical medial myosin network. Depletion of MTs in the placode leads to a failure of placodal cells to constrict apically, due to a loss of the pulsatile apical medial myosin II network. This medial myosin II network is required to drive contractions of the apical surface of cells in wild-type placodes, and interference with MTs, similar to interfering directly with myosin, affects these dynamic contractions. We show that the cytolinker protein Short Stop (Shot) is localized between apical medial actomyosin and MT (−) ends to mediate a functional interaction between the two networks and that interfering with Shot also impairs apical constriction and tube formation.

## Results

### Apical Microtubules Rearrange into a Longitudinal Network Concomitant with the Onset of Apical Constriction

To investigate MT organization during salivary gland formation, we used embryos expressing *fkhGal4* and *UAS-srcGFP*, which highlighted salivary gland cells with membrane-targeted GFP (Maybeck and Röper, 2009). In these embryos, we analyzed the distribution of MTs by labeling tyrosinated α-tubulin, a marker of dynamic or newly polymerized MTs, and acetylated α-tubulin, a marker of stable and longer-lived MTs (Westermann and Weber, 2003). We focused on the early stages of gland formation, where cells prior to invagination begin to elongate along their apicobasal axis and begin to constrict apically (early to midstage 11; Figures 1B–1D), eventually leading to the formation of a dimple that is the early invaginating tube (late stage 11; Figures 1B and 1E).

**Figure 2:**
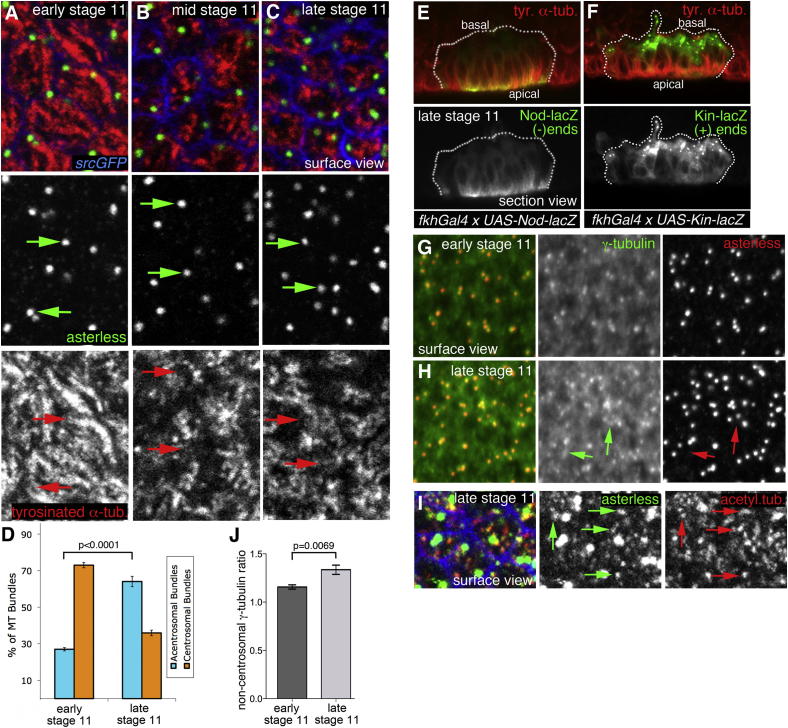
Microtubules Change from Centrosomal to Acentrosomal Nucleation/Anchoring during Early Invagination (A–C) Surface projections show that at early stage 11, the ends of many apical MTs colocalize with the centrosomal protein asterless (A), but through midstage 11 this changes (B) so that by late stage 11, centrosomes labeled by asterless less frequently colocalize with MT foci (C). Asterless, green; tyrosinated α-tubulin, red; *srcGFP*, blue. Arrows point to centrosomes and the matching positions in the MT channel. (D) Quantification of colocalization of MT bundle ends and centrosomes at early and late stage 11 (350 MT bundles from six different placodes for each stage; shown are mean ± SEM, p < 0.0001 using Student’s t test; see Table S1). (E and F) Section views of late stage 11 placodes: *Nod-LacZ*, a marker of MT (−) ends (E, green), accumulates apically in a flat region of a placode, indicating that MT (−) ends are located apically, whereas an MT (+) end marker, *Kin-LacZ*, is found basally (F, green). Tyrosinated α-tubulin, red. The white dotted lines mark placodal cells. (G and H) In surface projections of placodal cells, γ-tubulin becomes less tightly centrosome associated from early stage 11 to late stage 11. At early stage 11, the brightest γ-tubulin foci (green) colocalize with centrosomes (red) labeled by asterless (G). At late stage 11, in addition to centrosome foci, further noncentrosomal densities of γ-tubulin labeling have appeared within the placode (H, green arrows), not colocalizing with centrosomes marked by asterless. Panels are higher magnifications of boxes in Figures S1E and S1G. (I) When asterless labeling (green) at late stage 11 is analyzed at higher laser power, it shows, in addition to strong labeling of centrosomes, many fainter acentrosomal foci that colocalize with apical MT foci (arrows). Acetylated α-tubulin, red; *srcGFP*, blue. (J) Quantification of the mean noncentrosomal γ-tubulin fluorescence inside versus outside the placode (six placodes were analyzed for each stage; shown are mean ± SEM, p = 0.0069 using Student’s t test; see Table S1). See also Figure S1.

At early stage 11, MTs in most placodal cells and in the surrounding epidermis were arranged in a dense apical array, lying just underneath the apical plasma membrane, with few MTs extending into the cell interior (Figures 1C′ and 1F). From midstage 11 onward, MTs within the placode changed their major direction of orientation by 90° to align along the apicobasal cell axis (termed longitudinal MTs; Figures 1D′, 1E′, 1G, and 1H). This was particularly obvious in apically grazing sections (Figures 1F–1H and 2A–2C). The rearrangement was tightly coupled in time to the onset of constriction, in that it began in the dorsal-posterior corner of the placode, moving further ventral and anterior as the wave of apical constriction swept across the placode. Reorientation generally appeared to take place prior to apical constriction (Figures S1A–S1B″ available online). Longitudinal MT bundles showed strong acetylation of α-tubulin, a sign of stability, initiating from the apical surface (Figure 1I). Overall, acetylation of MTs was increased within the placode, especially within the highly constricting cells, in comparison to labeling of tyrosinated MTs (Figures S1C–S1D″′). MT bundles labeled by *Clip170-GFP*, a (+) TIP-binding protein (Stramer et al., 2010), that emanated from the apical surface showed dynamic behavior and growth of more basal plus ends (Figure 1J; Movie S1). A striking rearrangement of the MT cytoskeleton thus occurred concomitant with the earliest constriction of placodal cells (Figure 1K).

### Longitudinal Microtubule Arrays Emanate from the Apical Region in a Noncentrosomal Manner

Prior to onset of apical constriction and invagination, before midstage 11, most apical MTs were in close contact with apical centrosomes (Figure 2A), suggesting centrosomal nucleation. In contrast, in the rearranged MT network at late stage 11, most apical foci of tyrosinated and acetylated α-tubulin labeling did not colocalize with centrosomes (Figures 2B–2D; data not shown for acetylated α-tubulin). The apical foci of tubulin labeling at stage 11 corresponded to minus ends of MTs, as revealed through analysis of expression of *UAS-Nod-LacZ* and *UAS-Kin-LacZ*, two motor proteins that move toward and thus label the minus and plus ends of MTs, respectively (Figures 2E and 2F) (Clark et al., 1997), consistent with a similar orientation previously observed in fully invaginated gland cells at the end of embryogenesis (Myat and Andrew, 2002). Acentrosomal MTs in postmitotic epithelial cells, such as the placodal cells analyzed here, could be nucleated apically from γ-tubulin complexes not associated with the centrosomes (Bartolini and Gundersen, 2006; Feldman and Priess, 2012; Mogensen, 1999). We thus analyzed γ-tubulin distribution and found a small but significant increase in the amount of apical γ-tubulin that was not associated with the centrosomes labeled by the centrosomal protein asterless after MT rearrangement (Figures 2G and 2H; quantified in Figure 2J; Figures S1E–S1H′), suggesting that this noncentrosomal apical γ-tubulin might be involved in nucleating the longitudinal MTs. In addition, weaker foci of asterless were visible that colocalized with the ends of the rearranged longitudinal MT bundles (Figure 2I, arrows, MTs labeled using acetylated α-tubulin staining), suggesting that both γ-tubulin and asterless could form constituents of this apical noncentrosomal MT-organizing center.

### Microtubule Loss Affects Early Apical Constriction in the Placode

**Figure 3:**
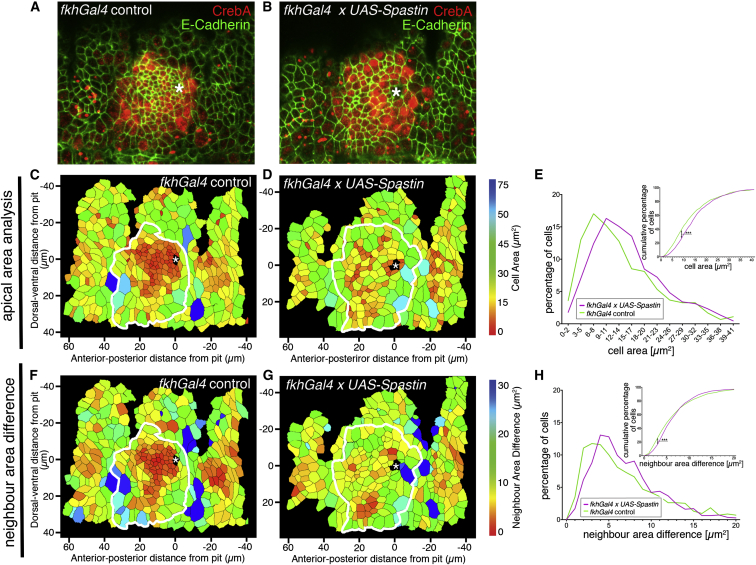
Depletion of the Microtubule Network Disrupts Apical Area Constriction in the Placode (A and B) The MT cytoskeleton was depleted using expression of *UAS-Spastin* under *fkhGal4* control. Representative surface view images of control (A) and MT-depleted (B) placodes at late stage 11, with E-cadherin (green) labeling cell outlines and CrebA (red) marking the cells of the placode. Asterisks denote the invagination point. (C and D) Heat maps corresponding to (A) and (B), respectively, indicating apical surface area size determined through automated tracing of E-cadherin-labeled cell boundaries. White lines denote the border of the placode (determined from CrebA labeling). (E) Quantification of apical area size in MT-depleted (*fkhGal4 x UAS-Spastin*) and control (*fkhGal4*) placodes at late stage 11, showing both the percentage of cells in different-size bins (large graph) and the cumulative percentage of cells relative to apical area size (inset: ^∗∗∗^p << 0.001 using Kolmogorov-Smirnov two-sample test; see Table S1). Ten placodes were segmented and analyzed for each condition; the total number of cells traced was N(*fkhGal4*) = 1,198 and N(*fkhGal4 x UAS-Spastin*) = 1,122. (F and G) Heat maps corresponding to (A) and (B), respectively, indicating the difference in apical surface area size between any given cell and its direct neighbors. (H) Quantification of (F) and (G) as for apical area differences above (N(*fkhGal4*) = 1,148, N(*fkhGal4 x UAS-Spastin*) = 1,117; inset: ^∗∗∗^p << 0.001 using Kolmogorov-Smirnov two-sample test; see Table S1). See also Figure S2.

In order to analyze whether the MT cytoskeleton in the salivary gland placode was important for early invagination steps, we made use of the MT-severing protein Spastin (Roll-Mecak and Vale, 2008). We overexpressed Spastin specifically in the placode, using *fkhGal4* and *UAS-Spastin*, and analyzed the effect on the MT cytoskeleton and cell behavior. Although the driver appeared not strong enough to affect MTs in all placodes, Spastin expression led to a clear reduction of MTs in 36% of placodes at early stage 11 of embryos of the genotype *fkhGal4 x UAS-Spastin* (n = 105; see also Experimental Procedures; Figure S2). We analyzed cell shapes using E-cadherin labeling of the apical circumference combined with microtubule labeling to identify affected placodes. We segmented apical cell outlines in control and MT-depleted placodes at mid to late stage 11, and from this could calculate both apical surface areas of placodal cells and also the dispersion of cells of different sizes (defined as the average difference in area between each cell and all of its neighbors; see Experimental Procedures). All placodes with a depleted MT cytoskeleton appeared strikingly different from the control (compare Figures 3A and 3B). Whereas in the control, cells began to constrict their apical surfaces in a highly ordered fashion starting from the dorsal-posterior corner (Figures 3A and 3C; Myat and Andrew, 2000), cells in MT-depleted placodes showed significantly fewer constricted apices (Figures 3B, 3D, and 3E). Those cells that did constrict were dispersed throughout the placode, unlike the wild-type cells, where constriction was clustered in the dorsal-posterior corner of the placode, where the invagination of the early tube initiates (Figures 3F–3H). When Spastin-expressing salivary glands were analyzed at stage 14 of embryogenesis, when most of the secretory part of the gland had invaginated in the wild-type (Figure S2F), glands with depleted MTs showed a spectrum of phenotypes consistent with aberrant invagination, from lumen defects to a complete failure to invaginate (Figures S2C–S2E). Similar effects on the MT cytoskeleton and gland invagination were also observed when another MT-severing AAA-ATPase, Katanin (Zhang et al., 2011), was expressed in the salivary gland placode (data not shown).

Thus, the MT organization that we observed concomitant with onset of apical constriction and tissue bending appeared to be an important functional part of the tube formation program.

### An Apical Medial Actomyosin Network Is Disrupted in the Absence of Microtubules

**Figure 4:**
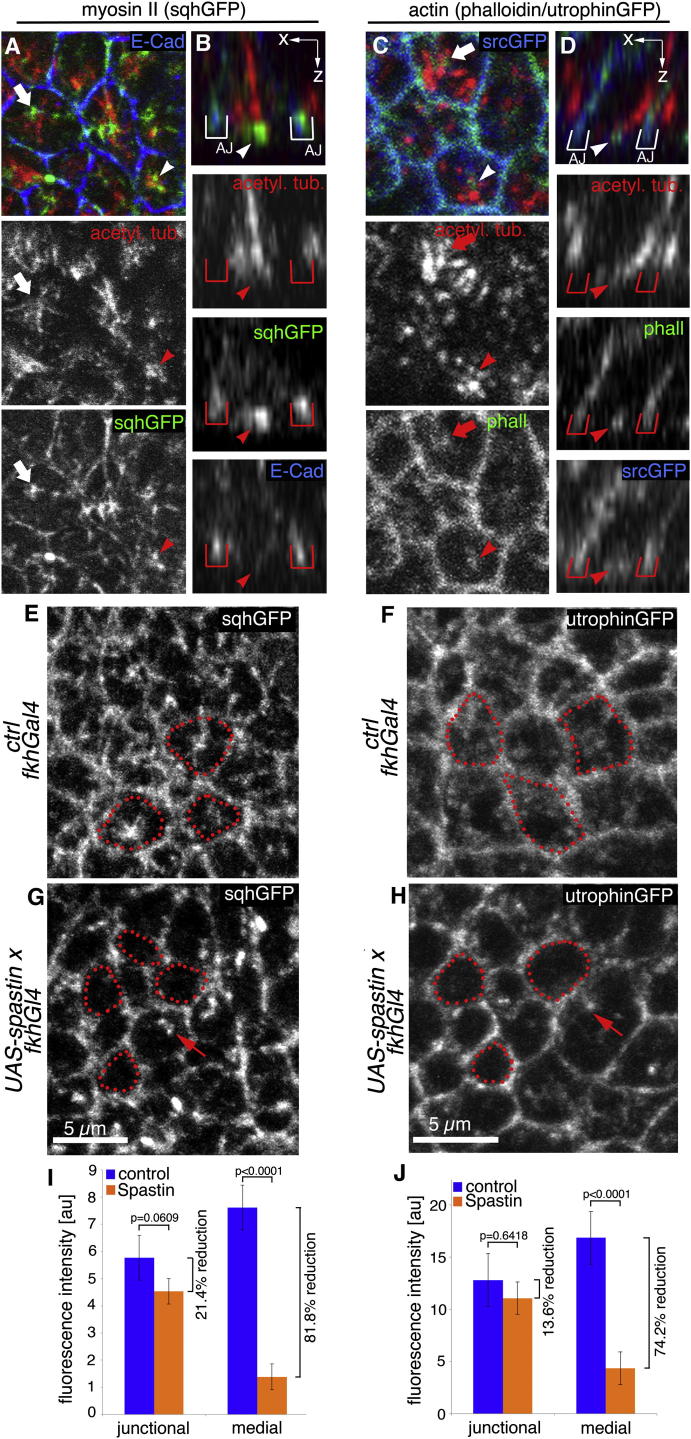
Loss of Microtubules Leads to Loss of the Apical Medial Actomyosin Network (A–D) Longitudinal MT bundles at late stage 11 terminate apically at foci of medial myosin (surface view, A; z section, B) and F-actin (surface view, C; z section, D). Acetylated α-tubulin, red; *sqhGFP*, green; phalloidin, green; E-cadherin, blue (A and B); *srcGFP*, blue (C and D). The arrows and arrowheads point to colocalization of MTs and medial actomyosin; the arrowheads in (A) and (C) point to the bundle that is displayed in the z sections in (B) and (D). Red brackets indicate positions of adherens junctions (AJ). (E–H) MT depletion using *UAS-Spastin* and *fkhGal4* disrupts the apical medial actomyosin network. Comparison of *sqhGFP* and *utrophinGFP* (to label actin) in control (E and F) and MT-depleted (G and H) placodes (the panels are higher magnifications of the boxes indicated in Figure S3). Red dotted lines highlight medial apical domains; arrows point to a myosin or actin remnant upon MT depletion. (I and J) Quantification of the effect of MT depletion on junctional and medial myosin (I), using *sqhGFP*, and actin (J), using *utrophinGFP*. Shown are mean ± SEM of placodal fluorescence intensity above epidermal base level; difference for junctional myosin is p = 0.0609 and for medial myosin is p < 0.0001, and for junctional actin is p = 0.6418 and for medial actin is p < 0.0001 using Student’s t test (see Table S1). See also Figure S3.

What could be the role of longitudinal MTs during the early steps of gland invagination in the wild-type? Apical constriction during epithelial morphogenesis is often mediated by apical actomyosin, located not only at the level of the adherens junctions as an actomyosin belt but also as a medial network of actomyosin underlying the apices of the cells (Blanchard et al., 2010; David et al., 2010; Martin et al., 2009, 2010). The salivary gland placode shows a strong increase in myosin II levels at the start of morphogenesis, consisting of apical junctional myosin as well as a prominent apical medial myosin II network (Röper, 2012), and myosin II is important for wild-type invagination (Blake et al., 1998). We thus investigated whether the MT cytoskeleton displayed any functional interactions with the apical actomyosin network, using a GFP-tagged transgene of nonmuscle myosin II regulatory light chain (MRLC; termed *sqhGFP* in flies) as a readout. Some medial myosin II could already be observed in all placodal cells prior to constriction starting within the placode, and also in placodal cells farther away from the invaginating pit that had not yet started to contract (Figures S3A and S3C). Very little overlap between apical MT bundles and medial myosin II could be found in these cells (Figures S3B–S3B″ and S3D–S3D″). Once the placodal MTs had undergone their 90° reorientation, however, the apically localized minus ends of longitudinal MT bundles were in close contact and just abutting apical medial myosin accumulations in 90% of cells analyzed (n = 350 cells, six embryos; Figure 4A). This was especially clear in confocal z sections (Figure 4B). The same close apposition could also be observed between minus ends of MTs and apical medial actin (Figures 4C and 4D). No close apposition between junctional actomyosin and MTs could be observed. When MTs were depleted using *UAS-Spastin* and *fkhGal4*, the MT-depleted cells not only displayed larger apical surface areas (as shown above) but also showed a significant decrease in the apical medial pool of myosin, an overall reduction of 81.8% (p < 0.0001; Figure 4E versus Figure 4G; quantified in Figure 4I; Figures S3E and S3F). A similar reduction of 74.2% was also observed for the apical medial actin (p < 0.0001; Figure 4F versus Figure 4H; quantified in Figure 4J; Figures S3G–S3J). In contrast, the junctional actomyosin located at the level of the adherens junctions was much less affected by MT depletion, with junctional myosin being reduced by only 21.4% (p = 0.0609; Figure 4I) and junctional actin decreased by only 13.6% (p = 0.6418; Figure 4J).

### An Apical Medial Actomyosin Network Drives Apical Constriction during Normal Tube Formation

**Figure 5:**
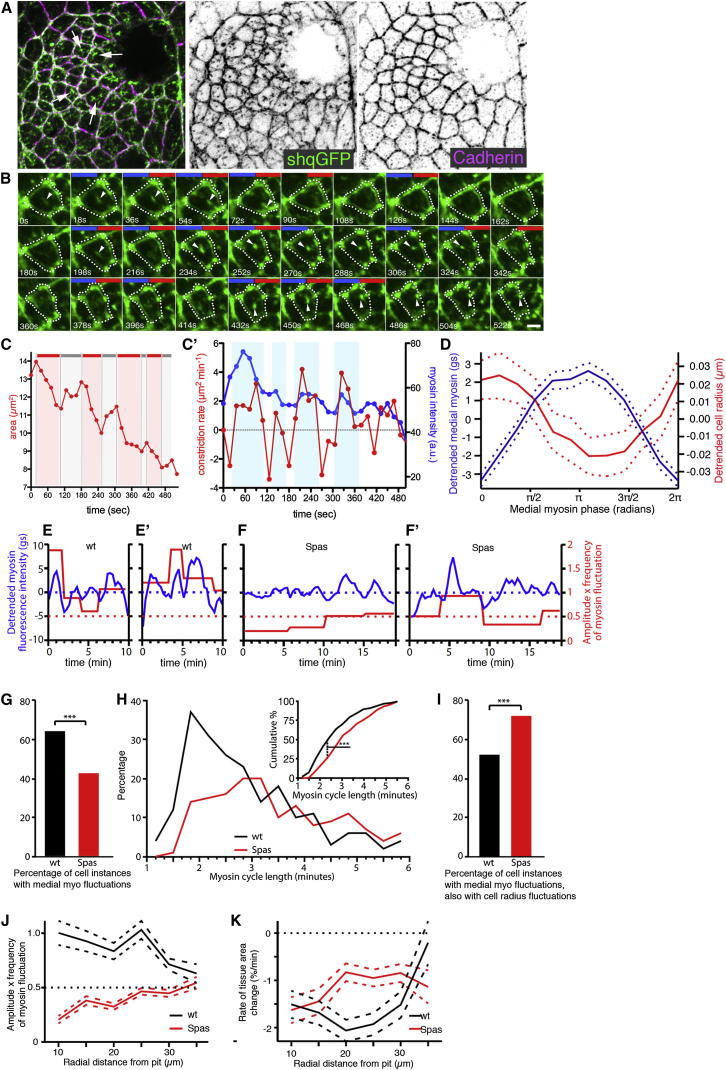
An Apical Medial Actomyosin Network Involved in Apical Constriction during Tubulogenesis (A) Myosin II (*sqhGFP*, green) is organized into an apical junctional and apical medial network across the salivary gland placode (Röper, 2012). The junctional myosin colocalizes with cadherin (magenta), and the medial myosin forms a network-like arrangement across many neighboring cells (arrows). (B) Still frames of a time-lapse movie of a *sqh*^*AX3*^*; sqh::sqhGFP42* embryo (see Figure S4A; Movie S2). The still frames show the fluctuations of medial myosin and the position of the cell cortex (dotted lines) of an exemplary placodal cell; arrowheads point to dynamic, pulsatile concentrations of myosin; blue bars indicate increased myosin II; red bars indicate increased constriction (see C′). The scale bar represents 2 μm. (C) Apical cell area (μm^2^) decreases in discrete pulses (red bars) followed by a period of relaxation and stabilization (gray bars). (C′) Quantification of the constriction rate (μm^2^ min^−1^; red) in comparison to medial myosin II intensity (blue) for a single exemplary cell in a placode. An increase in medial myosin II intensity is closely correlated with an increase in constriction rate. (D) Average medial myosin fluorescence (with trends removed; blue line; gs, grayscale) and cell radius (with trends removed; red line) plotted against phase of medial myosin fluctuation cycle. Two hundred and twelve full cycles of myosin (trough to trough) were pooled and averaged from nine wild-type embryo movies. Dotted lines show 95% confidence intervals. (E and F) Myosin fluorescence intensity (with trends removed, blue lines) and strength of myosin fluctuation (expressed as amplitude × frequency; red lines) for sample cells. Dotted red lines show the threshold value above which the strength of myosin activity was defined as being periodic. (E and E′) Two sample wild-type cells (WT). (F and F′) Two sample MT-depleted cells (Spas). Longer traces are shown for MT-depleted cells because of their longer cycle lengths. (G–K) Comparison of the average behavior of nine control (wild-type; black) and three MT-depleted embryos (Spas; red). For control embryo data, the number of tracked cell instances (see text) for which it could be established whether a cell was fluctuating or not was 2,877, of which 1,849 exhibited myosin fluctuations. Of the latter, apical radius fluctuated in 929. For MT-depleted embryo data, the number of cell instances was 3,711, 1,584, and 1,106, respectively. See Table S1 for details of statistical analysis. (G) Percentage of tracked placode cell instances for which medial myosin fluctuations could be detected (see also Movies S3 and S4). ^∗∗∗^p << 0.0001 using G test of independence. (H) Distribution of cycle lengths of cells showing myosin fluctuations. Inset: cumulative histograms indicating that cycle lengths of cells still showing fluctuations upon MT depletion were significantly increased. ^∗∗∗^p << 0.0001 using Kolmogorov-Smirnov test. (I) Percentage of cell instances with medial myosin fluctuations for which cell-radius fluctuations could also be detected. ^∗∗∗^p << 0.0001 using G test of independence. (J) Average strength of myosin fluctuation versus radial coordinate relative to the pit center. Dashed lines are 95% confidence intervals for pooled cell data. Dotted line at 0.5 amplitude × frequency marks the threshold below which cells were not considered to be periodic. (K) Average rate of change of tissue area versus radial coordinate relative to the pit center (same data as shown in Figures S4E and S4F). Dashed lines show respective 95% confidence intervals. See also Figures S4 and S5 and Movies S2, S4, and S5.

The apical medial myosin network in placodal cells was highly dynamic and showed pulsatile behavior, characterized by periodic increases and decreases in intensity combined with flows across the apical surface (Figures 5A and 5B; Figures S4A and S4B; Movie S2). In many cells, apical constriction occurred in a stepwise fashion, with pulses of constriction followed by periods of stabilization (example in Figure 5C). The periodic increase in myosin fluorescence intensity in individual cells correlated with an increase in the rate of apical constriction (Figures 5B and 5C′). This suggested that in the salivary gland placode, apical medial myosin was important for apical constriction and tube invagination. Apical MT foci also showed dynamic behavior (Figures S4C–S4D′; Movie S3), reminiscent of medial myosin foci.

To be able to assess the effect that MT depletion had on apical medial actomyosin dynamics and tissue contraction, we simultaneously visualized myosin (using *sqhGFP*) and cell membranes (using *GAP43-mCherry*) in wild-type and MT-depleted embryos. We tracked cell shapes in these movies and quantified medial myosin fluorescence (see Experimental Procedures; Movies S4 and S5). In the wild-type, the phase of average apical medial myosin density was consistently ahead of the phase of the inverse of the apical cell radius by approximately one-eighth of a cycle (Figure 5D; 212 full cycles analyzed from nine embryo movies), suggesting that myosin drove cell-shape changes. We classified each “cell instance” (meaning a movie frame through which a cell was tracked, sampled every ∼20 s) as being with or without myosin fluctuation and with or without apical area fluctuation (see Experimental Procedures). Example traces for wild-type and MT-depleted cells show clear differences in myosin fluctuations and strength of myosin activity (Figures 5E–5F′). We used a threshold myosin activity (frequency of fluctuation multiplied by amplitude) of 0.5, above which cells were considered to be fluctuating. This threshold value was set to exclude low-amplitude and/or low-frequency behavior that we would have visually classified as nonperiodic. Pooling cell instances, wild-type placodal cells spent a significantly greater proportion of tracked cell time with measurable myosin fluctuations (64% of tracked cell instances) compared to those in MT-depleted placodes (43%; Figure 5G). Furthermore, the cycle lengths of the myosin fluctuations were also significantly different. Wild-type cells had shorter cycle lengths, with relatively more cycles of less than 3 min in particular (Figure 5H), which in other tissues has been found to be the threshold below which productive tissue contraction, as opposed to unproductive area fluctuation, is achieved (Gorfinkiel and Blanchard, 2011). MT-depleted placodes probably retained some myosin fluctuations and apical area constriction due to an incomplete depletion of MTs across all placodal cells due to inhomogeneous *fkhGal4* expression (Figures S2B, S2B′, S3F, S3H, and S3J). Considering only cell instances with measurable myosin fluctuation, the percentage of these cell instances that also displayed area fluctuations was greater in MT-depleted placodes (72% versus 52% in wild-type; Figure 5I), suggesting that in this treatment, contractile myosin pulses were being less effectively harnessed to generate tissue contractile tension, probably a consequence of the increased myosin cycle length. The above differences between MT-depleted and wild-type placodes are the likely cause of the observed defects in apical constriction upon MT depletion.

We expected myosin contractile activity to lead to some combination of net contraction and/or tissue tension. We therefore investigated further the relationship between myosin activity and net contraction. Whereas there was a trend for increasing myosin activity toward the pit in the wild-type (Figure 5J), the strongest rate of apical area decrease, in addition to the early invaginating pit, was observed in a radial band 20–25 μm anterior-ventral to the pit (Figure 5K; Figure S4E). Thus, the pattern of myosin activity did not completely mirror the pattern of net area decrease in wild-type placodes. This was likely due to tissue tension, and hence resistance to contraction, varying in a radial pattern from the pit as the placode started to invaginate to form a 3D structure. In MT-depleted placodes, the rate of area change within the placode was reduced to less than half of wild-type rates within the 20–25 μm radial band, and myosin activity decreased toward the pit and was overall below the threshold set (Figure 5K; Figure S4F). Altogether, these data strongly suggest that in the wild-type, placode tension and constriction were driven to a large extent by an active medial actomyosin network (Figure S4B). MT depletion led to a reduction in both actomyosin fluctuation and fluctuation-driven productive area changes.

To further analyze whether the loss of dynamic apical constrictions of placodal cells upon MT depletion was due to effects on myosin II and not on other factors downstream of MTs, we disrupted myosin II function specifically in placodal cells. In *sqh*-null mutant embryos expressing *sqhGFP* under its endogenous promoter as the only source of functional MRLC (Royou et al., 2004), we specifically targeted *sqhGFP* for destruction by the proteasome only in placodal cells (using *UAS-deGradFP* and *fkhGal4*; see Experimental Procedures for the full genotype). Loss of *sqhGFP* function in placodal cells consistently led to aberrant glands with strong invagination defects at later stages of embryogenesis (Figures S4G–S4H′). Often, cells at stage 13 remained on the surface of the embryo with wide apical surfaces (Figures S4H–S4H″) but still showed a rearranged longitudinal MT network (Figure S4H″′). Time-lapse analysis of a membrane marker in these embryos revealed that cells did not efficiently constrict apically (Figures S4I and S4J; Movies S6 and S7). Thus, myosin II was crucial for the dynamic apical contractility of placodal cells.

To further exclude that the effect on medial actomyosin was an indirect consequence of a general disruption of cells, we analyzed markers of junctional integrity and apicobasal polarity in control placodes and those that had been depleted of MTs. In a wild-type placode at late stage 11, much MT reorientation had occurred and apical constriction had significantly progressed. In contrast, in MT-depleted placodes at late stage 11, about 1–2 hr after the usual start of cell constriction, only reduced apical constriction had occurred as described above. These MT-depleted placodes showed wild-type levels and localization of E-cadherin and Crumbs (Figure S5). This indicates that the loss of apical medial actomyosin and loss of correct apical constriction observed upon MT depletion were not secondary consequences of loss of junctional integrity or apicobasal polarity prior to this stage. Only at later stages (stage 12 and beyond) did MT-depleted placodes show occasional mislocalization of E-cadherin and Crumbs (Figures S5F, S5G, S5I, S5O, S5P, and S5R), possibly due to effects of prolonged MT depletion on either vesicle delivery or recycling.

Thus, the constriction of apical surfaces that initiated the invagination of the epithelial sheet depended on a dynamic apical medial actomyosin network, which in turn depended on the MT cytoskeleton for its assembly and/or maintenance.

### The Spectraplakin Shot Functionally Bridges Apical MT Ends and Medial Actomyosin

**Figure 6:**
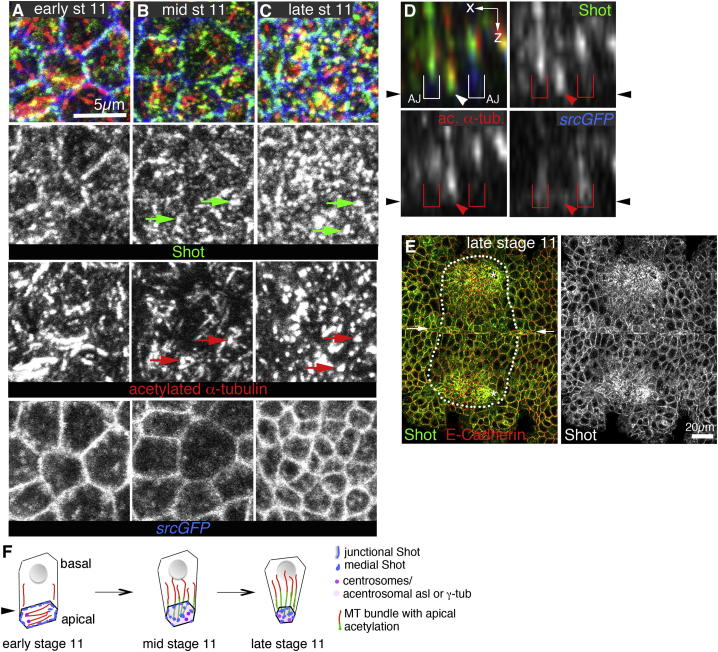
The Cytolinker Shot Relocalizes from the Cell Junctions to the Apical Ends of Microtubule Bundles during Early Invagination (A–C) The spectraplakin Shot contains actin-binding and MT-binding domains (see Figure S6A). At early stage 11, the majority of Shot (green) localizes to the cell cortex as described in other epithelia (A) (Röper and Brown, 2003), but during midstage 11, Shot relocalizes (B) to then colocalize with the ends of longitudinal MT bundles (red) by late stage 11 (C); shown are surface views. Arrows in (B) and (C) point to colocalization between Shot and MT foci. (D) z sections show Shot (green) localized at the end of an MT bundle (red). Eighty-three percent of MT bundles terminate in an apical focus of Shot (see Figure S6F). The arrowheads indicate the end of a microtubule bundle; the brackets indicate the positions of adherens junctions. (E) Overview surface scan at late stage 11 clearly shows the change in Shot localization (green) within the constricting secretory part of the placodes (marked by white dotted lines) compared to junctional Shot outside that placode that colocalizes with E-cadherin (red). Small arrows indicate the ventral midline. (F) Schematic of coordinated MT and Shot reorganization during early constriction. Black arrowheads in (D) and (F) indicate position of the apical domain. See also Figure S6.

**Figure 7:**
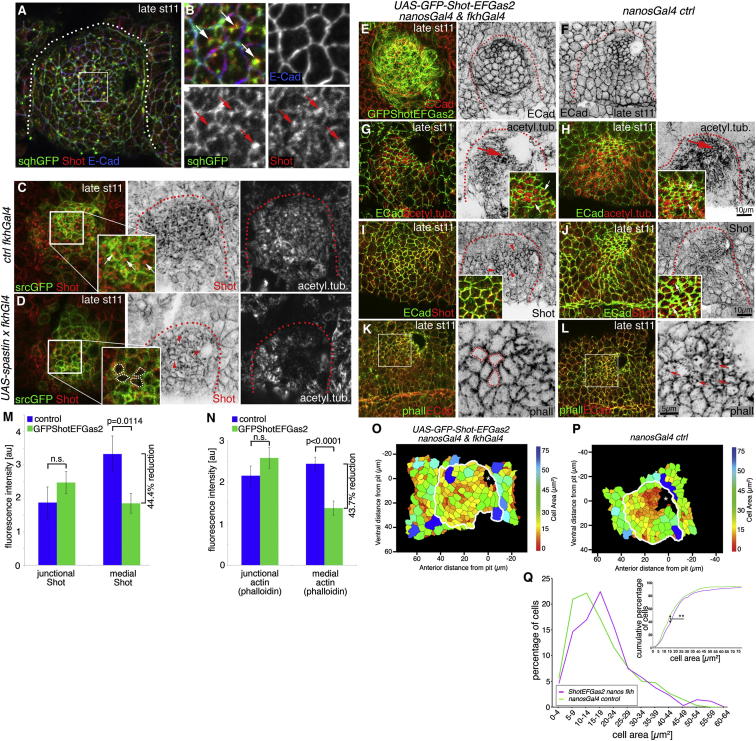
Shot Links Microtubules to Medial Actomyosin, and Medial Shot Is Required for Apical Constriction (A and B) At late stage 11, medial Shot (red) colocalizes with medial myosin (*sqhGFP*, green). The area marked by the white box in (A) is shown enlarged in (B). Arrows point to *sqhGFP*-Shot colocalization. (C and D) Depletion of MTs in the placode leads to a failure to relocalize Shot from the junctions to the apical medial region of placodal cells. In contrast to a control placode (C), where Shot (red) is localized to the apical medial region (see arrows in the inset), when MTs (acetylated α-tubulin) are depleted using *UAS-Spastin* and *fkhGal4* (D), Shot remains associated with the junctional area (green, *srcGFP*) in the placodal cells (inset: dotted lines mark medial regions of cells; arrowheads point to junctional Shot; quantified in Figure S7G). (E and F) Overexpression of *GFP-Shot-EFGas2* using *nanosGal4* and *fkhGal4* interferes with apical constriction (E) compared to the control (F). E-cadherin, red and as a single channel in (E) and (F); *GFP-Shot-EFGas2*, green in (E). (F) The single E-cadherin channel of the panel shown as a composite in (L). (G and H) MTs rearrange and appear stabilized when *GFP-Shot-EFGas2* is overexpressed (G) as in the control (H). Insets: MT bundle ends marked by acetylated α-tubulin (red; arrows) in between cell cortices marked by E-cadherin (green). (I and J) Endogenous Shot (red) remains cortical when *GFP-Shot-EFGas2* is overexpressed (I; red arrowheads) in contrast to the control, where Shot relocalizes to medial MT ends (J). Insets: Shot colocalizing with junctional E-cadherin (green) upon *GFP-Shot-EFGas2* expression (I), in contrast to medial Shot accumulations in the control (J; arrows). Shot levels are quantified in (M). Dotted lines in (A)–(J) mark the area of the placode, unless indicated otherwise. (K and L) Placodes overexpressing *GFP-Shot-EFGas2* often show reduced medial F-actin (K) compared to controls (L). Phalloidin (labeling F-actin; red in K and L and as a single channel in inverse panels); E-cadherin, green; white boxes mark areas magnified in inverse panels; dotted lines mark cells with no medial F-actin; arrows point to medial F-actin in the control. F-actin is quantified in (N). (M and N) Quantification of the effect of *GFP-Shot-EFGas2* overexpression on junctional and medial Shot (M) and actin (N; using phalloidin). Shown are mean ± SEM of placodal fluorescence intensity above epidermal base level; difference for junctional Shot is p = 0.2932 and for medial Shot is p = 0.0114, and for junctional actin is p = 0.2134 and for medial actin is p < 0.0001, using Student’s t test; n.s., nonsignificant (see Table S1). (O and P) Exemplary heat maps indicating the apical surface area size of *UAS-GFP-Shot-EFGas2*-expressing embryos (*nanosGal4* and *fkhGal4* control; M) and of control embryos (N) determined through automated segmentation of E-cadherin-labeled cell boundaries. The white lines denote the border of the placode. Asterisks denote the invagination point. (Q) Quantification of the apical area size in *GFP-Shot-EFGas2*-expressing (using *fkhGal4* and *nanosGal4VP16*) and control (*nanosGal4VP16*) placodes at late stage 11, showing both percentage of cells in different-size bins (large graph) as well as the cumulative percentage of cells relative to apical area size (inset: Kolmogorov-Smirnov two-sample test, ^∗∗^p << 0.01; see Table S1). For GFP-Shot-EFGas2 expression, four placodes, and for the control, three placodes were segmented and analyzed, and the total number of cells traced was N(*nanosGal4*) = 339 and N(*UAS-GFP-Shot-EFGas2 nanosGal4 & fkhGal4*) = 348. See also Figure S6.

What links the apical actomyosin and MT networks? The spectraplakin family of cytolinkers, containing both actin- and MT-binding domains, is a prime candidate to mediate such interaction (Röper et al., 2002; Suozzi et al., 2012). The sole fly spectraplakin is Shot, a large protein containing two N-terminal actin-binding calponin homology (CH) domains and an MT-binding Gas2 domain and C terminus (Figure S6A) (Röper et al., 2002). Shot was strongly expressed within the salivary gland placode during early invagination (Figures 6A and 6E for an overview). Shot was localized in a mostly cortical position at the apical surface of placodal cells at early stage 11, similar to its localization within the surrounding epidermal cells (Figures 6A and 6E). However, from midstage 11 onward, Shot lost much of its cortical concentration within the placode, appearing instead in large apical foci that often colocalized with the apical MT foci (Figures 6B–6E). In fact, 83% of MT bundles terminated in foci of Shot labeling at late stage 11 (Figure S6F). Shot colocalization with apical MT minus ends depended on the Gas2 domain and C terminus but not on its actin-binding CH domains (Figures S6C–S6E′). Shot foci also colocalized with the highest accumulation of apical medial myosin (Figures 7A and 7B), thus making it a prime candidate to bridge longitudinal MT bundles and apical medial actomyosin. MT depletion using UAS-Spastin blocked the Shot rearrangement: Shot remained localized to cortices and did not become concentrated in apical foci (Figure 7C versus Figure 7D; Figure S6G).

Shot is essential for oogenesis and egg formation (Röper and Brown, 2004), and therefore embryos lacking both zygotic and maternal pools of Shot during embryogenesis cannot be generated and analyzed. In embryos zygotically mutant for the null allele *shot*^*3*^, we could still observe residual protein and could not identify any phenotype in gland invagination (data not shown). To interfere with Shot function, we expressed the EF-hand and Gas2 domain of Shot (Figure S6A, green line), fused to GFP under UAS control (*UAS-Shot-EFGas2*; Maybeck and Röper, 2009) using a strong maternal driver, *nanosGal4VP16*, combined with *fkhGal4*. When *UAS-Shot-EFGas2* was expressed using *fkhGal4* alone, no perturbation of gland invagination could be observed (Maybeck and Röper, 2009). In combination with a strong maternal expression, placodal expression of this transgene was able to cause aberrant invagination with variable penetrance, with many invaginating cells not constricting in a wild-type pattern (Figures 7E, 7G, 7I, and 7K; exemplary area heat maps in Figures 7O and 7P; quantification in Figure 7Q; Figures S7H and S7I). Overexpression of *GFP-Shot-EFGas2* did not seem to affect MT rearrangement into longitudinal bundles (Figure 7G versus Figure 7H), but instead appeared to have a dominant-negative effect on endogenous Shot. Much of endogenous Shot remained cortically localized and did not move into medial apical foci (Figure 7I versus Figure 7J and insets; quantification in Figure 7M). Medial F-actin accumulation as a readout for apical medial actomyosin was reduced in a similar manner (Figure 7K versus Figure 7L; quantification in Figure 7N).

Thus, Shot localization to the minus ends of MT bundles within the apical medial domain, where it colocalized with the apical medial myosin network, was important for normal apical constriction during tube formation.

## Discussion

Our analysis shows that MTs play a crucial part in stabilizing and maintaining the medial actomyosin network driving the formation of a tube in fly embryos. We observe a very dynamic medial network of actomyosin that shows a pulsatile increase in intensity correlated with a decrease in apical surface area of individual cells. Such pulsatile actomyosin behavior is similar to what has been observed in the fly mesoderm prior to its invagination (Martin et al., 2009, 2010; Mason et al., 2013) and in the constricting flat sheet of amnioserosa cells during dorsal closure in the fly embryo (Blanchard et al., 2010). The average cycle length of myosin oscillations observed in the salivary gland placode of ∼120–180 s is comparable to the cycle length of ∼147 s (±43.5 s) observed for medial actomyosin oscillations during germband extension (Fernandez-Gonzalez and Zallen, 2011; Sawyer et al., 2011), whereas the faster process of mesoderm bending and internalization has a higher frequency of 82.8 s (±48 s) (Martin et al., 2009), and amnioserosa oscillations reduce from more than 4 min to 2 min as net tissue contraction commences during dorsal closure (Gorfinkiel and Blanchard, 2011; Gorfinkiel et al., 2009; Solon et al., 2009). The disruption of MTs using Spastin expression in the salivary gland placode decreases the proportion of time that cells show myosin oscillations, and strongly decreases the frequency of oscillation. In the absence of MTs, the majority of oscillations were in a time regime where they were unlikely to drive productive apical area decrease, consistent with previous results (Gorfinkiel and Blanchard, 2011).

There have been some previous hints that interplay between the MT cytoskeleton and actomyosin might also regulate morphogenesis in vertebrates. During *Xenopus* neurulation, the protein Shroom3 is necessary for the reorganization and accumulation of apical γ-tubulin and the assembly of an apical array of MTs (Lee et al., 2007). Shroom proteins, including Shroom3, bind Rho kinase to activate contractile actomyosin networks (Mohan et al., 2012). Thus, during cell-shape changes in the forming *Xenopus* neural tube, Shroom appears to be a potential linker between MTs and actomyosin. A *Drosophila* Shroom protein has only recently been identified, and so far mutant analysis suggests a role in recruitment of cortical myosin (Bolinger et al., 2010; Simões et al., 2014). A topologically related process in *Xenopus*, the apical constriction of bottle cells during gastrulation, depends on both intact actomyosin and microtubule networks, revealed when constriction upon treatment with specific chemical inhibitors was analyzed (Lee and Harland, 2007). Furthermore, tube formation in mammals, where live analysis is technically very challenging, also depends on apical constriction (Bush et al., 1990). Thus, data obtained on tubulogenesis in more accessible models will be a valuable guide to further studies in mammals.

Concomitant with medial actomyosin-driven apical constriction, the MT cytoskeleton organized into a noncentrosomal stabilized array combined with a topological rearrangement. MTs in most epithelia are acentrosomally nucleated, a feature conserved from flies to humans (Bartolini and Gundersen, 2006). Our analyses, and further examples from *Drosophila*, highlight the importance of a reorganization or changes in stability of the MT cytoskeleton to permit and/or drive morphogenetic processes: during dorsal closure, the MT cytoskeleton forms an apical parallel array important for zippering (Jankovics and Brunner, 2006); during cell flattening and elongation of the early amnioserosa cells, the MT cytoskeleton undergoes a perpendicular rotation to drive the observed “rotary cell elongation” (Pope and Harris, 2008); during formation of the tracheal system, noncentrosomal microtubules are formed that are important for the establishment of the correct branching pattern (Brodu et al., 2010); and during morphogenetic furrow progression in the eye, imaginal disc MTs appear apically stabilized (Corrigall et al., 2007). Thus, dynamic MT arrays susceptible to reorganization appear to form an important basis of several morphogenetic processes.

We show that the cytolinker Shot is the likely linker between the longitudinal MT bundles and the medial actomyosin network. When the relocalization of Shot to the medial apical domain during stage 11 is reduced, placodal cells often fail to invaginate properly, apical constriction is affected, and medial F-actin (likely together with medial myosin) is reduced. In its medial position, Shot could provide a physical coupling between MT bundles and apical actomyosin (MT bundle ends themselves show dynamic behavior within the apical domain, similar to myosin), but Shot could also recruit further factors crucial for actomyosin network assembly and function. The nature of these remains to be determined. The fact that the rearranged MT bundles show acetylation marks near the apical surface, suggesting their stabilization over time, also indicates that Shot might provide a stable, albeit not necessarily static, link between microtubule ends and apical medial actin.

Apical constriction based on medial myosin fluctuations is one of several important mechanisms that seem to operate to ensure proper tube invagination in the salivary glands, suggesting a complex “belt-and-braces” arrangement. We have shown previously that a multicellular actomyosin cable at the boundary of the salivary gland placode is under tension during invagination (Röper, 2012). This cable likely collaborates with medial actomyosin to provide a stabilizing tissue-sized ratchet. How all these mechanisms are coordinated will be important to determine in the future.

The MT cytoskeleton is emerging as a crucial player in morphogenesis, both through previously characterized functions such as regulation of adherens junction stability and the positioning, delivery, and turnover of membrane components, and now through a direct role in stabilizing the dynamic apical medial actomyosin network. This network in turn is crucial for the effective change in apical surface area and shape and for tube formation. Further studies will reveal whether the interplay between MTs and actomyosin revealed here is conserved during other invagination processes in invertebrates and vertebrates.

## Experimental Procedures

### Fly Stocks and Husbandry

For a full list of fly stocks and crosses, see Supplemental Experimental Procedures.

### Embryo Immunofluorescence Labeling and Confocal and Live Analysis

Embryos were fixed, stained, and imaged using standard procedures; for details, please refer to Supplemental Experimental Procedures.

### Automated Cell Segmentation, Tracking, and Myosin Quantification

For the analysis of apical cell area and neighbor analysis, images of fixed embryos of late stage 11 placodes (judged by the extent of tracheal development) were labeled with DE-cadherin to highlight cell membranes and with dCrebA or *fkhGal4*-driven GFP expression to mark salivary gland fate. For analyzing cell-contraction rates and myosin fluctuations, nine wild-type and three MT-depleted (Spastin) embryos labeled with *sqhGFP* and *Gap43-Cherry* were imaged live for between 7 and 28 min within a 50 min window at mid to late stage 11. Image stacks through apices of cells in the salivary placode were taken for both channels approximately every 20 s at 1-μm-depth intervals.

Cells were segmented in image stacks of fixed embryos, and cells were tracked and medial myosin fluorescence was quantified in movies with cell-tracking and analysis software as used previously (Blanchard et al., 2009, 2010; Butler et al., 2009; Gorfinkiel and Blanchard, 2011; Gorfinkiel et al., 2009). Briefly, the shape of the curved placode surface was identified in each z stack as a contiguous “blanket” spread over the cortical signal. Quasi-2D images for cell tracking containing clear cell cortices were extracted as a maximum-intensity projection of the 1- or 1.5-μm-thick layer of tissue below the blanket. These images were segmented using an adaptive watershedding algorithm, and in parallel cells were linked in time. Manual correction was used to perfect cell outlines for fixed embryos and to improve cell tracking in movies where the *GAP43-mCherry* fluorescence was sometimes faint. We imposed a coordinate system on each placode with the center of the pit at the origin, with anterior distance from the pit to the left and ventral distance down. Only cells of the salivary placode were used in subsequent analyses. These were distinguished from dCrebA staining in fixed embryos and from the cable surrounding the placode in the myosin channel of movies.

For the analyses of apical cell area in control versus MT-depleted fixed embryos, the clustering of cells of similar sizes was calculated for every cell within the placode as the average difference in apical area between a cell and all of its neighbors.

For the comparison of contraction rates and medial myosin behavior between control and MT-depleted embryos, tracked cells were first subjected to quality control. Cells were filtered using rules for inappropriate cell size, relative speed to neighbors, and rate of change in cell area. Incomplete cells at the edges of the field of view and those with short lineages were also removed from further analysis. Instantaneous tissue strain rates were calculated as previously for the remaining valid cells (Blanchard et al., 2009). Average medial myosin fluorescence intensity for each cell was calculated from the *sqhGFP* channel and myosin fluctuation analysis was performed as before (Blanchard et al., 2010), taking care to quantify myosin in the most apical layer(s) under the placode surface. Detrended cell-radius and myosin fluorescence intensity measures were calculated as the raw time series minus a smoothed time series trend (using a boxcar smoothing window of 6 min, larger than the maximum expected fluctuation cycle length).

Fluctuation analysis yielded cycle length, amplitude, and phase measures for each full cycle (defined for myosin as trough to trough and for cell radius as peak to peak). Periods of time for each cell that were not fluctuating were identified where amplitude multiplied by frequency was below a threshold that we established visually as being a good compromise between including real periodic behavior and excluding nonperiodic noise. The threshold for myosin fluctuation was 0.5, and 0.005 for cell-radius fluctuations. We then calculated the proportion of tracked cell time for which cells exhibited either myosin or cell-radius fluctuations.

### Quantification of Colocalization and Fluorescence Intensity

Colocalization analysis and fluorescence intensity quantifications were performed using standard procedures. For details, please refer to Supplemental Experimental Procedures.

### Statistical Analysis

Significance was determined using two-tailed Student’s t test, Kolmogorov-Smirnov test, or G test of independence. Results were considered significant when p < 0.05. Specific tests, test statistics, degrees of freedom, and p values can be found in Table S1.

## Supplemental Information

Document S1. Supplemental Experimental Procedures, Figures S1–S6, and Table S1

Movie S1. MT Dynamics in Invaginating Cells, Related to Figure 1J Time-lapse analysis of a group of cells undergoing invagination using *UAS-GFPClip170* under *fkhGal4* control, viewed in a section view, with the apical surface down. Frames are 3.87 s apart; the image shown is 32.5 × 25.5 μm. The green box in frame 1 indicates the position of the kymograph shown in Figure 1J.

Movie S2. Medial Myosin II Fluctuations in a Constricting Placodal Cell, Related to Figures 5B and S4A Time-lapse analysis of a single cell in the placode showing correlated myosin II fluctuations (visualized by *sqhGFP*), shown in stills in Figure 5B, and position in gland, shown in Figure S5A. Frames are 18 s apart; the image shown is 7.5 × 7.5 μm.

Movie S3. Apical Microtubule Bundle Ends and Membrane Marker in a Wild-Type Placode, Related to Figure S4D Representative time-lapse analysis of a wild-type placode labeled MT using *UASGFP-Clip170* under *fkhGal4* control (green, bottom panel) and membrane using *ECadherin-Tomato* (red, right panel). Shown is a single confocal slice grazing the apical surface of the central cells (peripheral cells are seen in section views). Frames are 7.74 s apart; the image shown is 31.8 × 29.9 μm; scale bar is 10 μm.

Movie S4. Medial Myosin II and Membrane Fluctuations in a Wild-Type Placode, Related to Figures 5E–5K Representative time-lapse analysis of a wild-type placode labeled using *sqhGFP* (green) and *GAP43mCherry* (red). Shown is a maximum-intensity projection of a stack covering the whole surface of the placode; frames are 14.5 s apart; the image shown is 54 × 54 μm.

Movie S5. Medial Myosin II and Membrane Fluctuations in a Microtubule-Depleted Placode, Related to Figures 5E–5K Representative time-lapse analysis of an MT-depleted placode (using *UAS-Spastin*) labeled using *sqhGFP* (green) and *GAP43mCherry* (red). Shown is a maximum-intensity projection of a stack covering the whole surface of the placode; frames are 14.5 s apart; the image shown is 59 × 59 μm.

Movie S6. Myosin and Membrane Analysis in a Placode with *sqhGFP* Targeted for Degradation by the Proteasome, Related to Figure S4I Representative time-lapse analysis of a placode of an embryo of the genotype *sqh*^*AX3*^*; sqh::sqhGFP42/UASdeGradFP; fkhGal4/GAP43mCherry*, where *sqhGFP* is targeted for destruction. *sqhGFP* is in green (right panel); *GAP43mCherry* is in red (middle panel). Shown is a maximum-intensity projection of a stack covering the whole surface of the placode; frames are 19.56 s apart; the image shown is 85 × 85 μm.

Movie S7. Cell-Shape Analysis in a Placode with *sqhGFP* Targeted for Degradation by the Proteasome, Related to Figure S4J Representative time-lapse analysis of a placode of an embryo of the genotype *sqh*^*AX3*^*; sqh::sqhGFP42/UASdeGradFP; fkhGal4/GAP43mCherry*, where *sqhGFP* is targeted for destruction. *sqhGFP* is in green (middle panel); *GAP43mCherry* is in red (right panel). Shown is a maximum-intensity projection of a stack covering part of the surface of the placode; frames are 7.46 s apart; the image shown is 22.7 × 44.8 μm.

Document S2. Article plus Supplemental Information
