# Supplementary material for: A Dynamic Microtubule Cytoskeleton Directs Medial Actomyosin Function during Tube Formation
Source: Dev Cell. 2014 Jun 9;29(5):562–76. doi: 10.1016/j.devcel.2014.03.023 (PMC4064686; doi:10.1016/j.devcel.2014.03.023)
Supplement: Document S1. Supplemental Experimental Procedures, Figures S1–S6, and Table S1 [file mmc1.pdf]

Developmental Cell, Volume *29*

Supplemental Information

A Dynamic Microtubule Cytoskeleton Directs Medial Actomyosin Function during Tube Formation

Alexander J.R. Booth, Guy B. Blanchard, Richard J. Adams, and Katja Röper

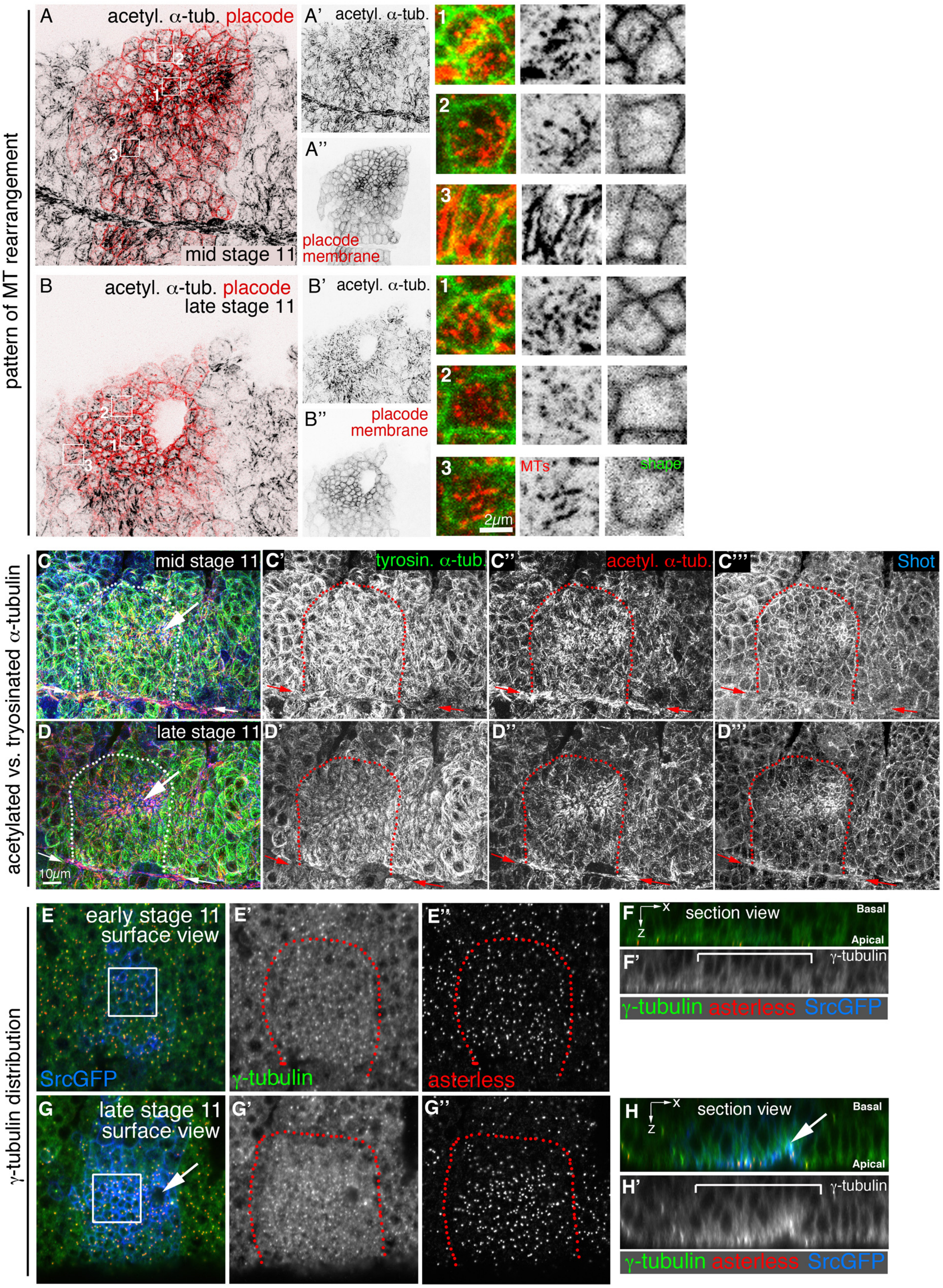
pattern of MT rearrangement
A
acetyl. α-tub. placode
mid stage 11
A'
acetyl. α-tub.
A''
placode membrane
B
acetyl. α-tub. placode
late stage 11
B'
acetyl. α-tub.
B''
placode membrane
MTs
shape
2μm
acetylated vs. tryosinated α-tubulin
C
mid stage 11
C'
tyrosin. α-tub.
C''
acetyl. α-tub.
C'''
Shot
D
late stage 11
D'
D''
D'''
10μm
γ-tubulin distribution
E
early stage 11 surface view
SrcGFP
E'
γ-tubulin
E''
asterless
F
section view
Basal
Apical
F'
γ-tubulin
γ-tubulin asterless SrcGFP
G
late stage 11 surface view
G'
G''
H
section view
Basal
Apical
H'
γ-tubulin
γ-tubulin asterless SrcGFP

Supplemental Figure S1 (related to Fig.1 and 2). Microtubule analysis during early gland invagination.

A-B'' The pattern of MT rearrangement across the placode during stage 11 mirrors the pattern of constriction, starting with the forming pit in the dorsal-posterior comer and moving anterior and ventral. MTs (marked by acetylated α-tubulin; black in **A**,**B** single in **A'**,**B'**, red in small panels) are shown in comparison to cell outlines (membranes of placodal cells marked by srcGFP; red in **A**,**B**, single in **A''**,**B''**, green in small panels). Small panels show examples of cells across the placode. Note that constricted and unconstricted cells with rearranged longitudinal MTs can be found (1 vs 2), but parallel apical bundles are usually only seen in unconstricted cells (3).

C-D''' Comparison of acetylated α-tubulin (red in **C**,**D**, single in **C''**, **D''**) and tyrosinated α-tubulin (green in **C**,**D**, single in **C''**, **D''**) in mid and late stage 11 placodes. Note that tyrosinated α-tubulin levels appear fairly even across epidermis and placode, whereas acetylated α-tubulin levels appear increased in constricting placodal cells. Placodes marked by dotted lines, small arrows point to ventral midline, larger arrows point to the forming pit. Shot (blue) marks cell outlines.

E-H' γ-tubulin distribution during early gland invagination Comparison of the centrosomal marker asterless (red) to γ-tubulin (green) labeling at early stage 11 (**E, F**) and late stage 11 (**G,H**). **E** and **G** show stacks of confocal images grazing the surface of the placode (surface views), whereas **F** and **H** are section views in z. The white boxes in **E, G** indicate the area shown in Fig. 2G,H; the red dotted lines in surface views and the white brackets in section views indicate the area of the placode (identified by *srcGFP* labeling using *fkhGal4*; blue). Arrows point to the invaginating pit at late stage 11.

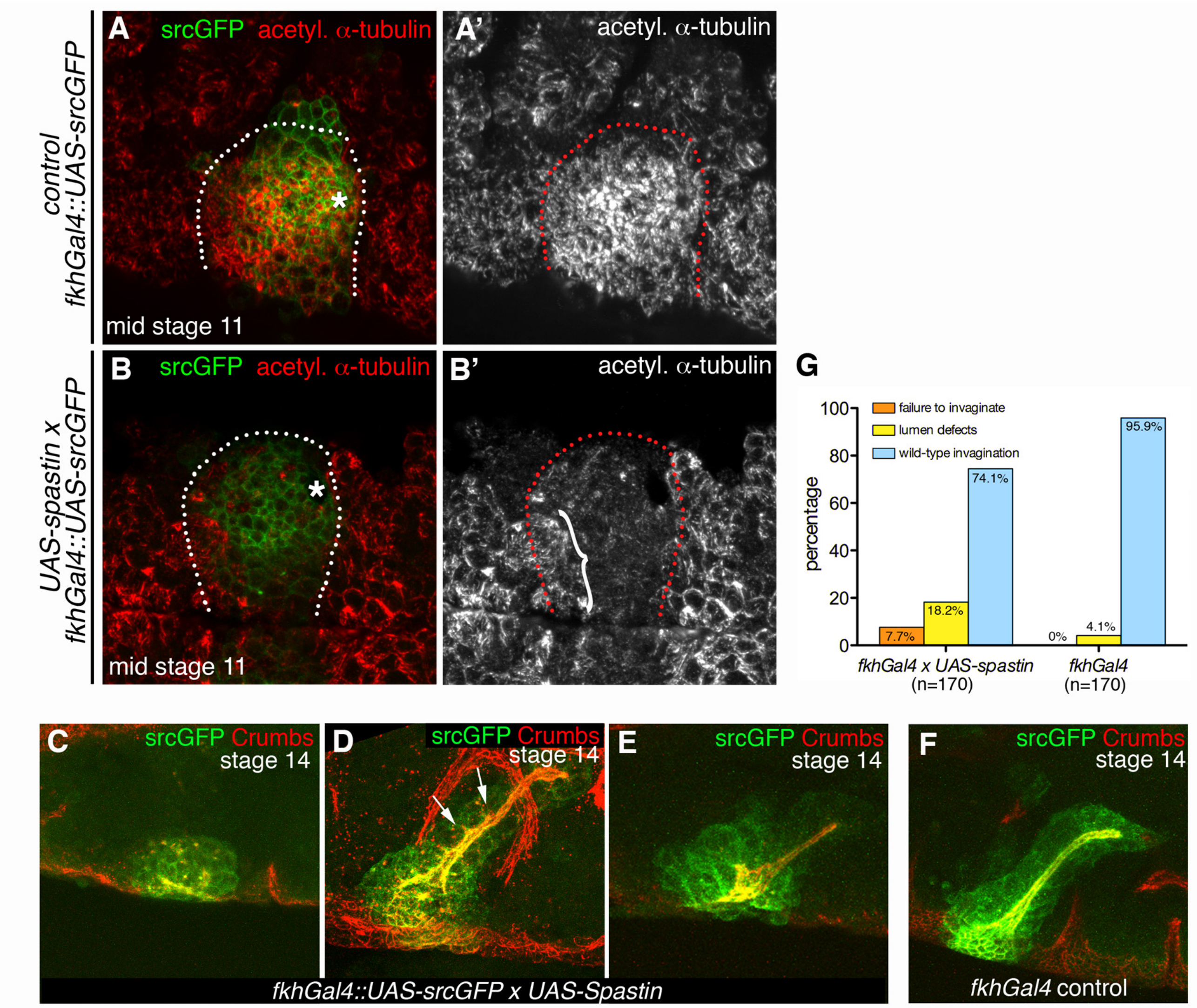

Supplemental Figure S2 (related to Fig.3). Depletion of microtubules in the salivary gland placode and resulting late embryonic phenotypes.

Expression of *UAS-Spastin* under *fkhGal4* control was used to deplete MTs specifically from the salivary gland placode.

Control placodes (**A**; placode area marked by *srcGFP*, green) show dense MTs (red) within the placode, whereas expression of UAS-Spastin under control of *fkhGal4* within the placode (**B**) leads to depletion of MTs (red) in 36% of placodes at stage 11. The dotted lines mark the boundary of the placode, the asterisks mark the invagination point. Note the loss of strong acetylated α-tubulin staining in the MT-

depleted placode. White bracket indicates placodal area where MT-depletion is incomplete, due to reduced levels of *fkhGal4* expression in this area.

C-F Analysis of MT-depleted placodes at stage 14. In contrast to wild-type placodes that have nearly completely invaginated at stage 14 (**F**), MT-depleted placodes show a range of phenotypes from complete failure to invaginate (**C**) to ectopic branching (**D**; arrows) and irregular invagination (**E**). Apical membrane is marked by Crumbs (red), and placode marked by *srcGFP* (green).

G Quantification of phenotypes at stage 14 of embryos expressing *UAS-Spastin* driven by *fkhGal4* or control embryos with only *fkhGal4* (n=170 glands for each genotype). Note that similar to stage 11, only a fraction of glands show depleted MTs when Spastin is expressed at this stage, and these MT-depleted glands show the phenotypes illustrated in **C-E**.

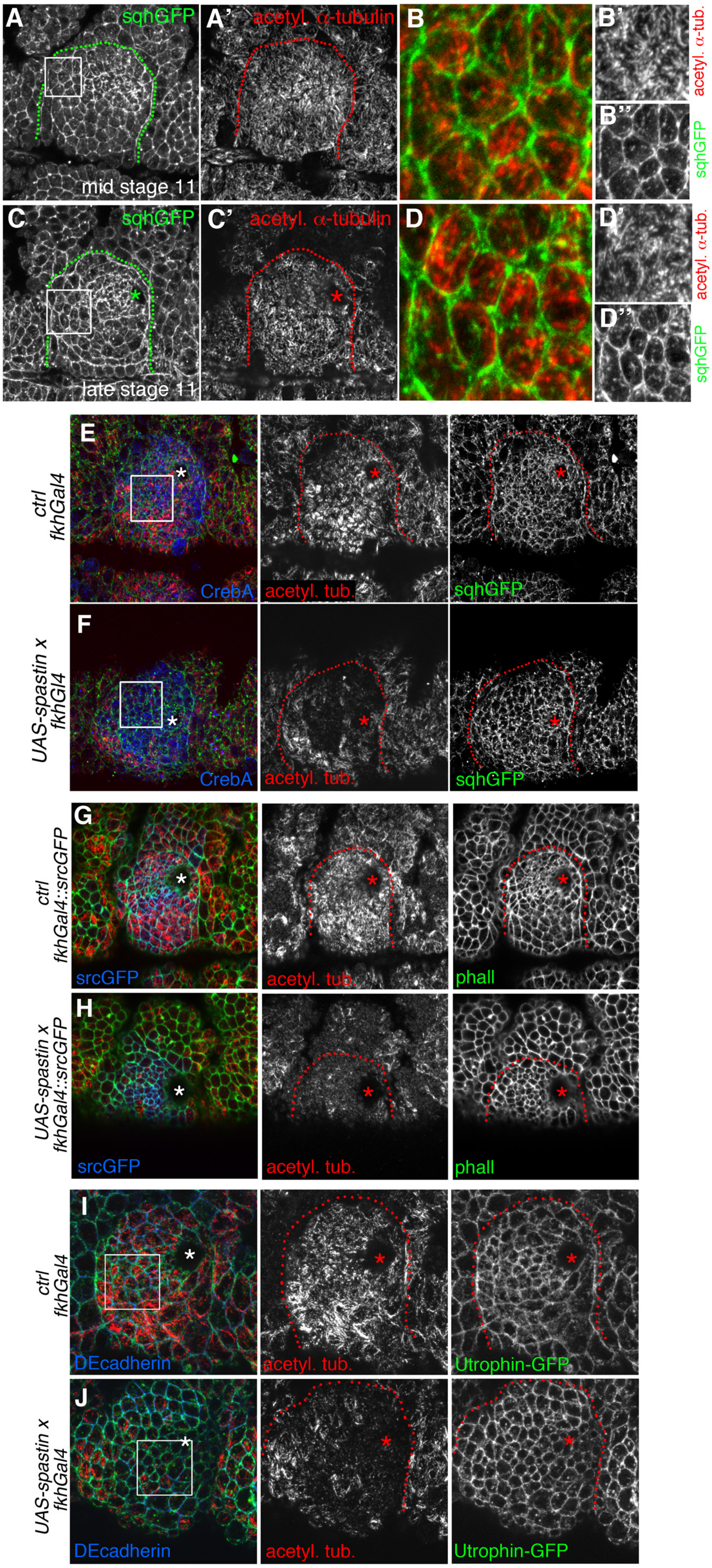

A
sqhGFP
mid stage 11
A'
acetyl. α-tubulin
B
B'
acetyl. α-tub.
B''
sqhGFP
C
sqhGFP
late stage 11
C'
acetyl. α-tubulin
D
D'
acetyl. α-tub.
D''
sqhGFP
ctrl fkhGal4
E
CrebA
acetyl. tub.
sqhGFP
UAS-spastin x fkhGl4
F
CrebA
acetyl. tub.
sqhGFP
ctrl fkhGal4::srcGFP
G
srcGFP
acetyl. tub.
phall
UAS-spastin x fkhGal4::srcGFP
H
srcGFP
acetyl. tub.
phall
ctrl fkhGal4
I
DEcadherin
acetyl. tub.
Utrophin-GFP
UAS-spastin x fkhGal4
J
DEcadherin
acetyl. tub.
Utrophin-GFP

Supplemental Figure S3 (related to Fig.4). Analysis of actomyosin in control and microtubule-depleted placodes.

A-D'' Apical medial myosin localisation in non-constricted cells at mid (**A**,**B**) and late (**C**,**D**) stage 11 in the anterior region of the placode. *sqhGFP* (**A**,**C**, green in **B**, **D** and **B''**,**D''**), acetylated α-tubulin (**A'**,**C'**, red in **B**, **D** and **B'**,**D'**). White boxes in **A**, **C** indicate the magnified areas in **B** and **D**. Note that in these unconstricted cells with mostly apical parallel MT bundles, MTs and medial myosin do not colocalise.

E-J Effect of microtubule-depletion on actin and myosin in the placode.

E,F Analysis of *sqhGFP* (green) in control placodes (**E**; *fkhGal4*) and MT-depleted placodes (**F**; *UAS-Spastin x fkhGal4*); placode area labeled by CrebA (blue); acetylated α-tubulin (red). The white boxes indicate the areas shown in higher magnification in Fig. 4E,G.

G-J Analysis of actin (green; using phalloidin [**G,H**] or *utrophinGFP* [**I,J**) in control placodes (**G,I**; *fkhGal4*) and MT-depleted placodes (**H,J**; *UAS-Spastin x fkhGal4*); placode area labeled by *srcGFP* (blue); acetylated α-tubulin (red); DE-Cadherin (blue). The white boxes in **I, J** indicate the areas shown in higher magnification in Fig. 4F,H.

Asterisks mark the invagination point, dotted lines mark the circumference of the placode.

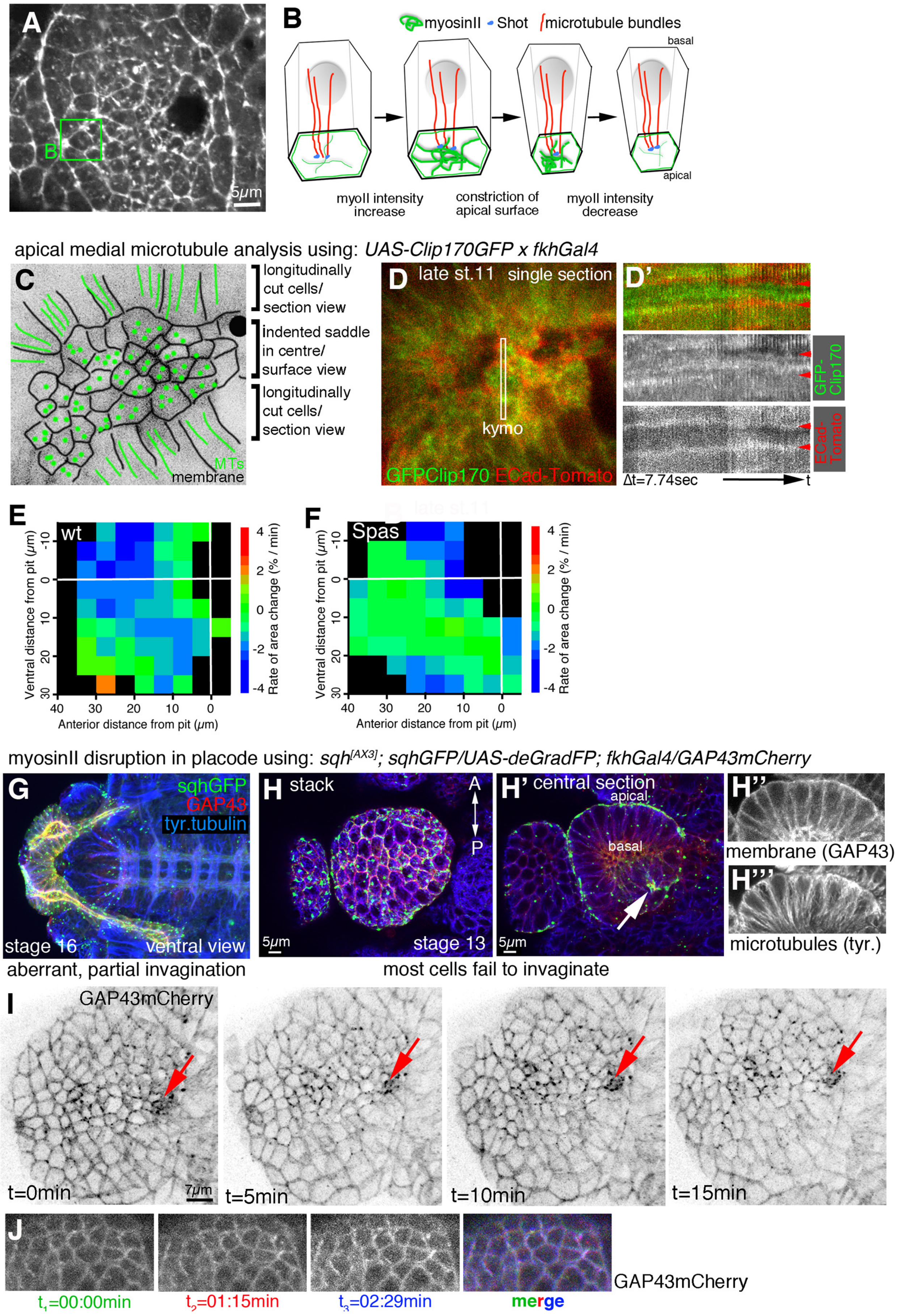
A
B
5μm
B
myosinII
Shot
microtubule bundles
basal
apical
myoII intensity increase
constriction of apical surface
myoII intensity decrease
apical medial microtubule analysis using: UAS-Clip170GFP x fkhGal4
C
longitudinally cut cells/ section view
indented saddle in centre/ surface view
longitudinally cut cells/ section view
MTs
membrane
D
late st.11
single section
kymo
GFPClip170
ECad-Tomato
D'
GFP-Clip170
ECad-Tomato
Δt=7.74sec
t
E
wt
Ventral distance from pit (μm)
Anterior distance from pit (μm)
Rate of area change (% / min)
F
Spas
Ventral distance from pit (μm)
Anterior distance from pit (μm)
Rate of area change (% / min)
myosinII disruption in placode using: sqh[AX3]; sqhGFP/UAS-deGradFP; fkhGal4/GAP43mCherry
G
sqhGFP
GAP43
tyr.tubulin
stage 16
ventral view
aberrant, partial invagination
H
stack
A
P
5μm
stage 13
H'
central section
apical
basal
5μm
most cells fail to invaginate
H''
membrane (GAP43)
H'''
microtubules (tyr.)
I
GAP43mCherry
t=0min
7μm
t=5min
t=10min
t=15min
J
t1=00:00min
t2=01:15min
t3=02:29min
merge
GAP43mCherry

Supplemental Figure S4 (related to Fig.5). Placodal cells show a contractile medial actomyosin network crucial for apical constriction and gland invagination.

A Overview of a placode of a *sqh*^{AX3}*; sqh::sqhGFP42* embryo used for time-lapse analysis. The green box indicates the area shown as stills in Fig.5B.

B Schematic model of apical medial actomyosin function in placodal cells: cycles of increase of medial myosin lead to apical surface constriction, probably through constriction of the whole network and through pulling of actomyosin fibres on associated junctional complexes. The medial actomyosin network is stabilised by longitudinal MT bundles, with the cytolinker Shot providing the link between MTs and actin.

C-D' Live analysis of medial MT end dynamics in placodal cells. MTs were labeled using *GFP-Clip170*. **C** is a schematic of the area shown in the still image in **D** and in Supplemental Movie 3 (MTs in green and cell boundaries in black), with only the cells marked in the middle being visible in a surface view, and the more peripheral cells being viewed in section views, due to the saddle-like shape of the tissue at this stage. **D** Still image of Suppl.Movie 3; the white box is indicating the area that is shown in the kymograph in **D'**. Frames are 7.74 sec apart, the last frame is at 379.26 sec, cell junctions are marked by E-Cadherin-Tomato (red); arrowheads in **D'** indicate the positions of two cell boundaries.

E,**F** Average rates of change of tissue area across the salivary gland placode. Anterior is left, ventral is down, with pit located at [0,0] where white lines cross. Data pooled from cell tracks sampled approx. every 20 seconds covering 50 minutes of mid to late stage 11. Grid squares with fewer than N = 20 cell instances were discarded. **E** Data pooled from 9 control embryos (total N cell instances = 4895). **F** Data pooled from three MT-depleted embryos (total N cell instances = 5126).

The same data are also depicted in different form in Fig. 5K.

G-H''' Targeting placodal myosin II for degradation impairs apical constriction and gland invagination. In flies of the genotype *sqh*^{AX3}*; sqh::sqhGFP42/UAS-deGradFP; fkhGal4/GAP43mCherry sqhGFP* is the only source of myosin II and is targeted for degradation by the proteasome specifically in the salivary glands. These embryos show glands that are only partially invaginated at stage 16 (**G**) or glands where most cells failed to constrict apically and invaginate, leaving two bulges on the outside of the embryo (**H**; in this panel anterior is up; arrow in **H'** points to the lumen of the only invaginated portion, the early pit). *sqhGFP* (green); *GAP43mCherry* (red); tyrosinated α-tubulin (blue). Note that *sqhGFP*, when targeted by *deGradFP*, accumulates in bright foci throughout the cells that appear to be non-functional as noted previously (Caussinus et al., 2012). Longitudinal MTs (**H'''**; compare to membrane marker in **H''**), similar to controls, can be seen in unconstricted cells in these embryos.

I,J Time-lapse analysis of glands in embryos of the genotype *sqh*^{AX3}*; sqh::sqhGFP42/UASdeGradFP; fkhGal4/GAP43mCherry* shows that (apart from the very early pit) placodal cells do not significantly constrict their apices. Panels in **I** show four time-points over 15 min of Suppl.Movie 6 (GAP43mCherry). **J** shows three individual time-points and a color-coded overlay of Suppl.Movie 7.

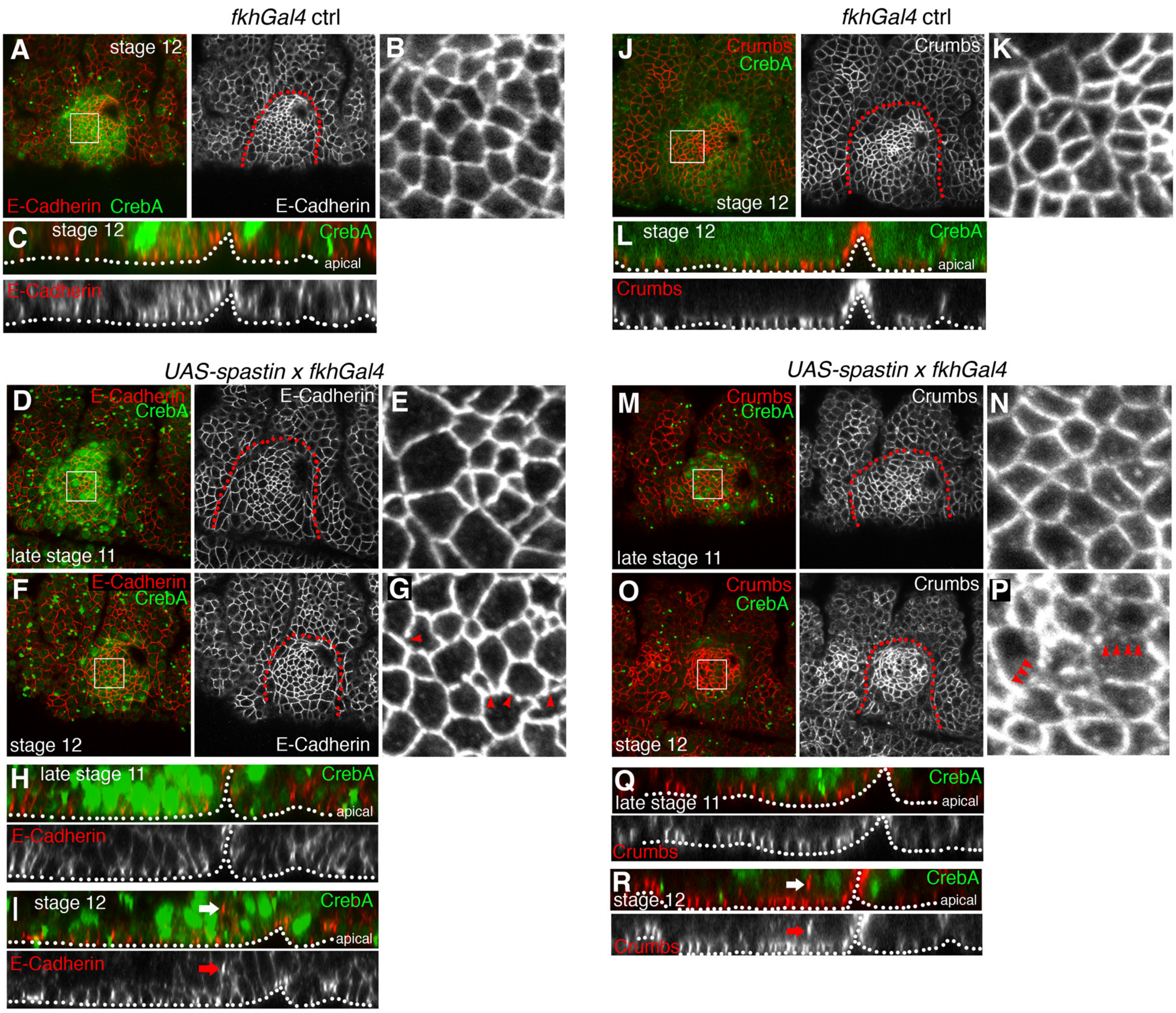

Supplemental Figure S5 (related to Fig.5). Effect of microtubule-depletion on E-Cadherin and Crumbs localisation in the placode.

A-C Control placodes (*fkhGal4*) labeled for E-Cadherin at stage 12. Shown are surface views (**A,B**) and section views (**C**) for CrebA (green), E-Cadherin (red). **B** shows a higher magnification of E-Cadherin of the area indicted by the box in **A**.

D-I MT-depleted placodes (*UAS-Spastin x fkhGal4*) at late stage 11 (**D**,**E** and **H**) and at stage 12 (**F**,**G** and **I**). Shown are surface views (**D-G**) and section views (**H,I**) for CrebA (green), E-Cadherin (red). Whereas E-Cadherin staining is indistinguishable from control placodes at late stage 11, at stage 12 discontinuities (arrowheads in **G**)

and internal foci (arrows in **I**) can be observed at times. Dotted lines mark the boundary of the placode (deduced from CrebA staining); dotted lines in section views mark the apical surface.

J-L Control placodes (*fkhGal4*) labeled for Crumbs at stage 12. Shown are surface views (**J,K**) and section views (**L**) for CrebA (green), Crumbs (red). **K** shows a higher magnification of E-Cadherin of the area indicted by the box in **J**.

M-R MT-depleted placodes (*UAS-Spastin x fkhGal4*) at late stage 11 (**M**,**N** and **Q**) and at stage 12 (**O**, **P** and **R**). Shown are surface views (**M-P**) and section views (**Q,R**) for CrebA (green), Crumbs (red). Whereas Crumbs staining is indistinguishable from control placodes at late stage 11, at stage 12 discontinuities (arrowheads) and internal foci (arrows) can be observed at times. Dotted lines mark the boundary of the placode (deduced from CrebA staining); dotted lines in section views mark the apical surface.

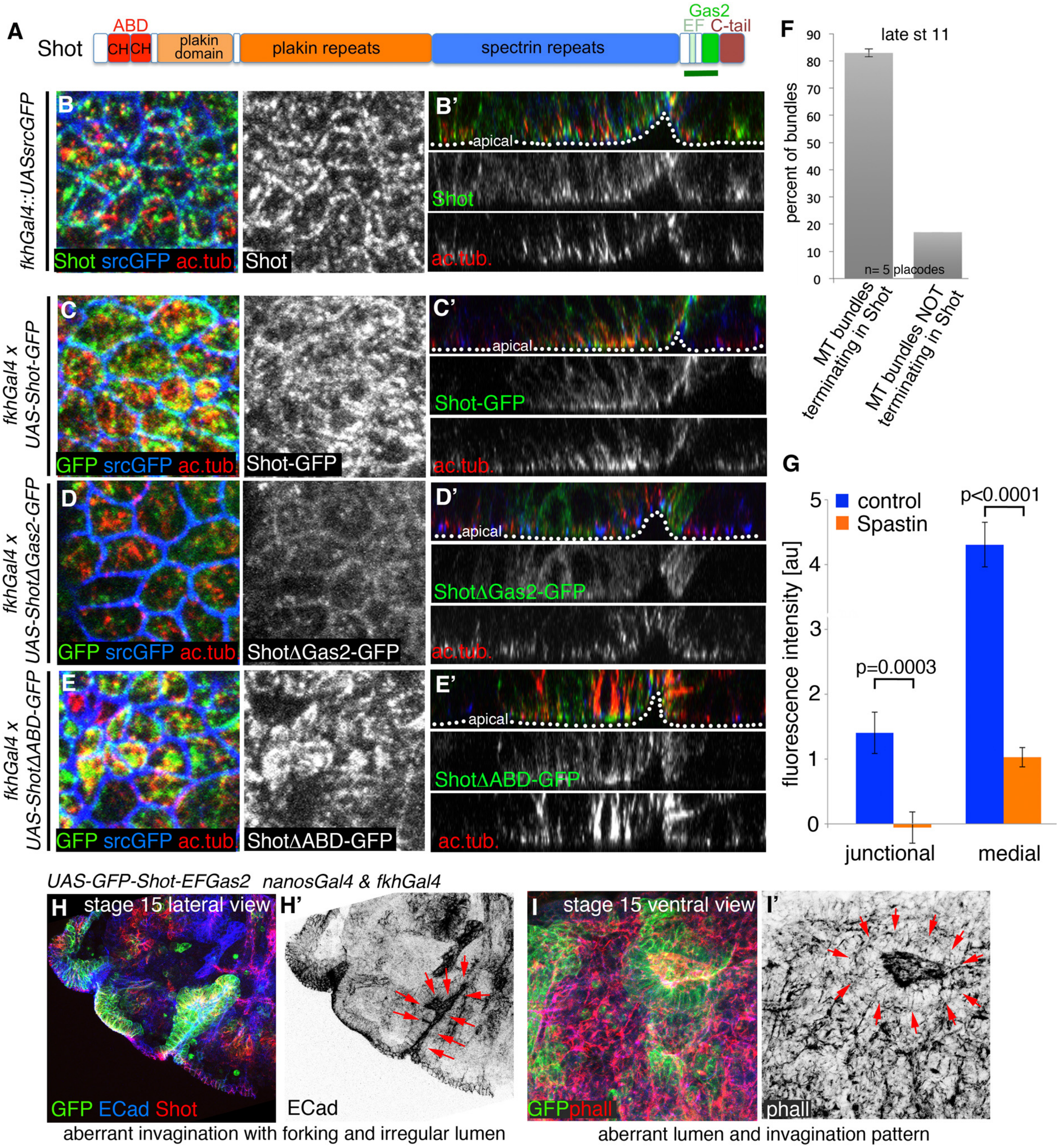

Supplemental Figure S6 (related to Figs 6 and 7). Analysis of Shot function in the placode.

A Schematic of Shot protein domain composition, with an actin-binding domain (ABD) composed of two calponin-homology (CH) domains at the N-terminus, and a microtubule-binding C-terminus including EF-hands and a Gas2-domain. The green

bar below the protein schematic indicates the region contained in Shot-EFGas2-GFP.

B-E' Shot requires its Gas2 domain to localise to the apical ends of microtubule bundles in the placode. At late stage 11, antibody labeling for Shot (green, **B,B'**) is indistinguishable from localisation of a GFP-tagged full length Shot protein (*UAS-Shot-GFP* under *fkhGal4* control; green, **C,C'**), both strongly label the apical (-) ends of MTs (red) within the placode. In contrast, a GFP-tagged version of Shot lacking the Gas2 domain (green, **D,D'**) fails to localise to MT apical (-) ends, whereas the GFP-tagged version of Shot lacking the ABD but containing the Gas2 domain (green, **E,E'**) strongly localised along the whole length of MTs, bundled them and stabilised them as evident from increased acetylated α-tubulin staining. Shown are surface (**B-E**) and section views (**B'-E'**). The apical surface is indicated by white dotted lines in the section views. Placodal membranes are marked with *srcGFP* (blue).

F 83% of MT bundles terminate in an apical focus of Shot. Shown are mean +/- SEM of bundles analysed in 5 placodes.

G Quantification of the effect of MT-depletion (using *UAS-Spastin* and *fkhGal4*) on Shot localisation within the placode at late stage 11 (shown are mean +/- SEM of placodal fluorescence intensity above epidermal base level; junctional: $p=0.0003$, medial $p<0.0001$ using Student's t-test, see Table 1). Upon MT-depletion, junctional Shot in the placode is reduced to levels of junctional Shot outside the placode, whereas medial Shot is strongly reduced.

H-I' Late gland phenotypes observed at stage 15 when endogenous Shot function was disrupted by expression of *UAS-GFP-Shot-EFGas2* under *nanosGal4* and *fkhGal4* control. Glands can show aberrant invagination with forking and irregular lumina (**H,H', I, I'**). *UAS-GFPShot-EFGas2* (green); endogenous Shot (red in **H**); E-Cadherin (blue in **H** and inverse in **H'**); phalloidin (red in **I** and inverse in **I'**). Red arrows point to the irregular lumen and gland shapes in both examples.

Figure and Panel:	Test Statistic	Sample Size / Degrees of Freedom	P-Value
Fig. 2D, centrosomal-acentrosomal MT ends	Unpaired Student's t-test t=8.854	df=10, n(early st11) = 326 MT bundles, 6 placodes n(late st11) = 384 MT bundles, 6 placodes	P<0.0001
Fig. 2J, acentrosomal γ-tubulin	Unpaired Student's t-test t=3.4	df=10, n(stage 10) = 6 placodes n(late stage 11) = 6 placodes	P=0.0069
Fig. 3E, cell area percentage of cells	K-S test. Dmax=0.1303 Dcrit for P<0.001=0.0810	n(control) = 1198 cells n(Spas) = 1122 cells, 10 placodes	P<<0.001
Fig. 3H, neighbour area difference percentage	K-S test. Dmax=0.0965 Dcrit for P<0.001=0.0810	n(control) = 1148 cells n(Spas) = 1117 cells, 10 placodes	P<<0.001
Fig. 4I, junctional myosin, ctrl vs MT-depleted	Unpaired Student's t-test t=1.912	df=58 n(control) = 30 cells, 3 placodes n(Spas) = 30 cells, 3 placodes	P=0.0609
Fig. 4I, medial myosin, ctrl vs MT-depleted	Unpaired Student's t-test t=6.556	df=58 n(control) = 30 cells, 3 placodes n(Spas) = 30 cells, 3 placodes	P<0.0001
Fig. 4J, junctional utrophin/actin, ctrl vs MT-depleted	Unpaired Student's t-test t=0.4676	df=58 n(control) = 30 cells, 3 placodes n(Spas) = 30 cells, 3 placodes	P=0.6418
Fig. 4J, medial utrophin/actin, ctrl vs MT-depleted	Unpaired Student's t-test t=4.214	df=58 n(control) = 30 cells, 3 placodes n(Spas) = 30 cells, 3 placodes	P<0.0001
Fig. 5G, Is percentage of cell time with myosin cycles the same for wt as for Spas?	G-Test of independence G=7747	n(control) = 2887 cells, 9 placodes n(Spas) = 3711 cells, 3 placodes	P<<0.001
Fig. 5H, length of myosin cycles	K-S test. Dmax=0.233 Dcrit for P<0.001=0.137	n(control) = 207 cells, 9 placodes n(Spas) = 154 cells, 3 placodes	P<<0.001
Fig. 5I, Is percentage of myosin cycling time that also has cell radius cycles the same for wt as for Spas?	G-Test of independence G=6410	n(control) = 2778 cells, 9 placodes n(Spas) = 2690 cells, 3 placodes	P<<0.001
Suppl.Fig. S6G, junctional Shot, ctrl vs MT-depleted	Unpaired Student's t-test t=3.729	df=88 n(control) = 40 cells, 4 placodes, n(Spas) = 50 cells, 5 placodes	P=0.0003
Suppl.Fig. S6G, medial Shot, ctrl vs MT-depleted	Unpaired Student's t-test t=9.333	df=88 n(control) = 40 cells, 4 placodes, n(Spas) = 50 cells, 5 placodes	P<0.0001
Fig. 7M, junctional Shot, ctrl vs GFP-Shot-EFGas2 expressing	Unpaired Student's t-test t=1.061	df=58 n(control) = 20 cells, 2 placodes n(Spas) = 40 cells, 4 placodes	P=0.2932

Fig. 7M, medial Shot, ctrl vs GFP-Shot-EFGas2 expressing	Unpaired Student's t-test t=2.613	df=58 n(control) = 20 cells, 2 placodes n(Spas) = 40 cells, 4 placodes	P=0.0114
Fig. 7N, junctional phall/actin, ctrl vs GFP-Shot-EFGas2 expressing	Unpaired Student's t-test t=1.248	df=198 n(control) = 100 cells, 6 placodes n(Spas) = 100 cells, 6 placodes	P=0.2134
Fig. 7M, medial phall/actin, ctrl vs GFP-Shot-EFGas2 expressing	Unpaired Student's t-test t=4.553	df=198 n(control) = 100 cells, 6 placodes n(Spas) =100 cells, 6 placodes	P<0.0001
Fig. 7Q, cell area percentage of cells	K-S test. Dmax=0.1315 Dcrit for P<0.01=0.1244	n(control) = 339 cells, 3 placodes n(Spas) = 348 cells, 4 placodes	P<<0.01

Supplemental Table S1 (related to Figs 2, 3, 4, 5, 7 and S6). Statistical analyses.

Significance was determined using two-tailed Student's *t*-test, Kolmogorov-Smirnov (K-S) test or G-test of independence. Results were considered significant when P<0.05. Specific tests, test statistics, degrees of freedom and p-values are all indicated above.

Supplemental Experimental Procedures

Fly stocks and husbandry

The following transgenes were used: *sqh^{AX3}; sqh::sqhGFP42*, and *yw; sqh::sqhGFP42; sqh::sqhGFP40* (Royou et al., 2004): *sqh^{AX3}; sqh::sqhGFP42; GAP43-mCherry*, *sqh::sqhmCherry* (Martin et al., 2009), *fkhGal4* (Henderson and Andrew, 2000; Zhou et al., 2001)[kind gift of Debbie Andrew]; *fkhGal4::srcGFP* (Maybeck and Röper, 2009), *UAS-Spastin* on X (Sherwood et al., 2004); *UAS-Katanin* (Christian Dahmann lab); *UAS-deGradFP II* (Caussinus et al., 2012); *UAS-GFP-Clip170* (Stramer et al., 2010); *sqh::UtrophinGFP* (Rauzi et al., 2010); *UASp-GFP-Shot-EFGas2* (Maybeck and Röper, 2009).

The following fly stocks were obtained from Bloomington and are described in FlyBase: *enGal4; nanosGal4VP16*.

To deplete MTs, virgins of *fkhGal4* were crossed to males of *UAS-Spastin (X)*. Using anti-tubulin immunofluorescence, successful MT-depletion was observed in 36% (n=105) of the resulting offspring of the genotype *UAS-Spastin (X)/+ or Y;+; fkhGal4/+*. The timing of *fkhGal4* expression, with expression starting only at the end of stage 10, is the likely reason that only 36% of placodes showed successful depletion.

To analyse myosin II localisation in MT-depleted placodes, virgins of *yw/+; sqh::sqhGFP42/+; sqh::sqhGFP40/fkhGal4* were crossed to males of *UAS-Spastin (X)/Y; sqh::sqhGFP42/+; sqh::sqhGFP40/+*. To induce degradation of *sqhGFP* specifically in the placode, males of *UAS-deGradFP/+; fkhGal4/+* were crossed to virgins of *sqh^{AX3}; sqh::sqhGFP42; GAP43mCherry*, leading to all male embryos being mutant for *sqh*. Embryos with *sqhGFP* targeted for degradation could be identified by the large aggregates that *sqhGFP* forms within the placode under these conditions (Caussinus et al., 2012). To affect endogenous Shot by expression of *UASp-GFP-Shot-EFGas2*, virgins of *nanosGal4VP16* (III) were crossed to males of

fkhGal4::UASp-GFP-Shot-EFGas2 (III), and the resulting offspring (F1) was backcrossed and the F2 embryos analysed. Note that expression of *UASp-GFP-Shot-EFGas2* by *fkhGal4* alone does not produce any dominant-negative effect (Maybeck and Röper, 2009).

In order to be able to compare equivalent developmental time points when salivary gland placode invagination was disrupted by expression of transgenes under *fkhGal4* control, we used the developmental state of the tracheal pits and their invagination as a timing control.

Embryo Immunofluorescence Labelling, Confocal, and Live Analysis

Embryos were fixed, stained and imaged using standard procedures, for details please refer to the Supplemental Experimental Procedures.

Embryos were collected on grape- or apple juice-juice plates and processed for immunofluorescence using standard procedures. Briefly, embryos were dechorionated in 50% bleach, fixed in 4% formaldehyde, and stained with phalloidin or primary and secondary antibodies in PBT (PBS plus 0.5% bovine serum albumin and 0.3% Triton X-100). anti-Crumbs and anti-E-Cadherin antibodies were obtained from the Developmental Studies Hybridoma Bank at the University of Iowa; the anti-dCrebA antibody was a kind gift from Deborah Andrew (Andrew et al., 1997); the anti Shot spectrin repeats was made in our lab (Röper and Brown, 2003); anti tyrosinated α-tubulin (YL1/2; Millipore); anti acetylated α-tubulin (clone 6-11B-1; Sigma); anti γ-tubulin (clone GTU-88; Sigma); anti asterless was a kind gift from Jordan Raff (Stevens et al., 2009). Secondary antibodies used were Alexa Fluor 488/Fluor 549/Fluor 649 coupled (Molecular Probes) and Cy3 and Cy5 coupled (Jackson ImmunoResearch Laboratories), and rhodamine-phalloidin was from Molecular Probes. Samples were embedded in Citifluor (Citifluor Ltd.) or Vectashield (Vectorlabs).

Images of fixed samples were acquired on an Olympus FluoView 1000/1200 or a Zeiss 780 Confocal Laser scanning system as z-stacks to cover the whole apical surface of cells in the placode. Z-stack projections were assembled in ImageJ or Imaris (Bitplane), 3D rendering was performed in Imaris.

For live time-lapse analysis of *sqh^{AX3}; sqh::sqhGFP42; GAP43-mCherry* embryos (alone or in combination with *fkhGal4 UAS-Spastin*), of embryos of the genotype *sqh^{AX3}; sqh::sqhGFP42/deGradFP; fkhGal4/GAP43-mCherry*, and of embryos of the genotype *GFP-Clip170/ECad-Tomato; fkhGal4*, the embryos were dechorionated in bleach, rinsed in water and attached to a coverslip with the ventral side up using heptane glue and covered with Halocarbon Oil 270 (saturated with water). Embryos harbouring *fkhGal4 UAS-Spastin* were identified by the loss of apical constriction due to MT-depletion as identified in the static analysis. Time-lapse sequences were acquired on a Zeiss 780 Laser scanning system as z-stacks. Z-stack projections to generate movies were assembled in ImageJ or Imaris.

Quantification of colocalisation

<u>*Microtubules versus myosin, centrosomes or Shot:*</u> Overlap of signal of either MT-labeling and medial myosin, MT-labeling and centrosomes, or MT-labeling and Shot localisation within the placode was counted in ImageJ using the 'Cell Counter' plugin (Kurt De Vos, Institute for Translational Neuroscience, University of Sheffield), using either images of fixed *sqh^{AX3};sqhGFP42* and *yw;sqhGFP42;sqhGFP40* stained for acetylated α-tubulin or *fkhGal4 x srcGFP* embryos stained for acetylated α-tubulin and asterless, or acetylated α-tubulin and Shot. The placode area was identified from the position of the circumferential myosin cable (Röper, 2012) or the extent of *srcGFP* expression. Colocalisation was confirmed by visually scanning through the apical-most 2 μm (5 confocal sections at 0.5 μm apart).

Fluorescence intensity quantifications

Non-centrosomal γ-tubulin: Images were taken of salivary gland placodes (marked with dCrebA or *fkh-Gal4::srcGFP*) and surrounding tissue at early stage 11 and at late stage 11. Any γ -tubulin signal that colocalised with centrosomes (marked with anti-asterless antibody) was masked using Imaris software (Bitplane). Maximum intensity projections of the apical surface of placodal cells were generated using 5 optical section separated by 0.5 μm each in z. For each embryos analysed, fluorescence measurements were made in a 10x10 μm box inside the placode (three measurements) and in the surrounding tissue (four measurements). The background fluorescence was determined by taking three fluorescence intensity measurements in areas of varying sizes outside the embryo. Corrected total cell fluorescence (CTCF) for γ -tubulin inside and outside the placode was calculated as follows:

CTCF = IntegratedDensity – (area x mean background fluorescence).

Mean fluorescence intensity per μm was calculated by dividing the sum of the CTCF by the sum of area measurements, and the ratio of [mean fluorescence intensity – inside placode]/[mean fluorescence intensity- outside placode] was generated for each projection. Six embryos were analysed at both stages.

Medial versus junctional myosin II, actin and Shot in control and MT-depleted placodes, or control and GFP-Shot-EFGas2-expressing placodes :

Images were taken of salivary gland placodes (identified by the surrounding myosin cable or by *srcGFP*) at late stage 11.

Fluorescence intensity per μm^2 was determined in ImageJ. Measurements were taken for the junctional pool and medial pool of myosin (using *sqhGFP*), actin (using *utrophinGFP*) or Shot from ≥10 cells outside the placode (i.e. outside the circumferential placodal actomyosin cable), giving a mean value for extraplacodal junctional actin/myosin/Shot and extraplacodal medial actin/myosin/Shot. Cells were then randomly selected from the placode (within the circumferential placodal

actomyosin cable; those selected in MT depleted placodes were also selected to be completely MT free; numbers of cells and placodes are listed in Table 1) and fluorescence intensity per μm^2 was determined for junctional and medial actin/myosin/Shot. A 7 pixel wide (0.728 μm) line was drawn around the junction (marked by DE-Cadherin staining) and used to measure the intensity of fluorescence at junctions. A second reading was taken over the corresponding medial area (now marked to be inside the 7 pixel line) to measure medial fluorescence.

For each placodal cell, the extraplacodal junctional and extraplacodal medial means were subtracted from the placodal junctional and medial measurements, respectively, to show how much the placodal junctional and medial fluorescence was raised above the mean junctional and mean medial fluorescence of the surrounding tissue.

Supplemental References

Andrew, D.J., Baig, A., Bhanot, P., Smolik, S.M., and Henderson, K.D. (1997). The Drosophila dCREB-A gene is required for dorsal/ventral patterning of the larval cuticle. Development *124*, 181-193.

Caussinus, E., Kanca, O., and Affolter, M. (2012). Fluorescent fusion protein knockout mediated by anti-GFP nanobody. Nature Struct. & Mol. Biol. *19*, 117-121.

Henderson, K.D., and Andrew, D.J. (2000). Regulation and function of Scr, exd, and hth in the Drosophila salivary gland. Dev. Biol. *217*, 362-374.

Martin, A.C., Kaschube, M., and Wieschaus, E.F. (2009). Pulsed contractions of an actin-myosin network drive apical constriction. Nature *457*, 495-499.

Maybeck, V., and Röper, K. (2009). A targeted gain-of-function screen identifies genes affecting salivary gland morphogenesis/tubulogenesis in Drosophila. Genetics *181*, 543-565.

Rauzi, M., Lenne, P.F., and Lecuit, T. (2010). Planar polarized actomyosin contractile flows control epithelial junction remodelling. Nature *468*, 1110-1114.

Röper, K. (2012). Anisotropy of Crumbs and aPKC Drives Myosin Cable Assembly during Tube Formation. Dev. Cell *23*, 939-953.

Röper, K., and Brown, N.H. (2003). Maintaining epithelial integrity: a function for gigantic spectraplakin isoforms in adherens junctions. J. Cell Biol. *162*, 1305-1315.

Royou, A., Field, C., Sisson, J.C., Sullivan, W., and Karess, R. (2004). Reassessing the role and dynamics of nonmuscle myosin II during furrow formation in early Drosophila embryos. Mol. Biol. Cell *15*, 838-850.

Sherwood, N.T., Sun, Q., Xue, M., Zhang, B., and Zinn, K. (2004). Drosophila spastin regulates synaptic microtubule networks and is required for normal motor function. PLoS Biol. *2*, e429.

Stevens, N.R., Dobbelaere, J., Wainman, A., Gergely, F., and Raff, J.W. (2009). Ana3 is a conserved protein required for the structural integrity of centrioles and basal bodies. J. Cell Biol. *187*, 355-363.

Stramer, B., Moreira, S., Millard, T., Evans, I., Huang, C.Y., Sabet, O., Milner, M., Dunn, G., Martin, P., and Wood, W. (2010). Clasp-mediated microtubule bundling regulates persistent motility and contact repulsion in Drosophila macrophages in vivo. J. Cell Biol. *189*, 681-689.

Zhou, B., Bagri, A., and Beckendorf, S.K. (2001). Salivary gland determination in Drosophila: a salivary-specific, fork head enhancer integrates spatial pattern and allows fork head autoregulation. Dev. Biol. *237*, 54-67.
