# Supplementary figures and images for: A Dynamic Microtubule Cytoskeleton Directs Medial Actomyosin Function during Tube Formation
Source: Dev Cell. 2014 Jun 9;29(5):562–76. doi: 10.1016/j.devcel.2014.03.023 (PMC4064686; doi:10.1016/j.devcel.2014.03.023)

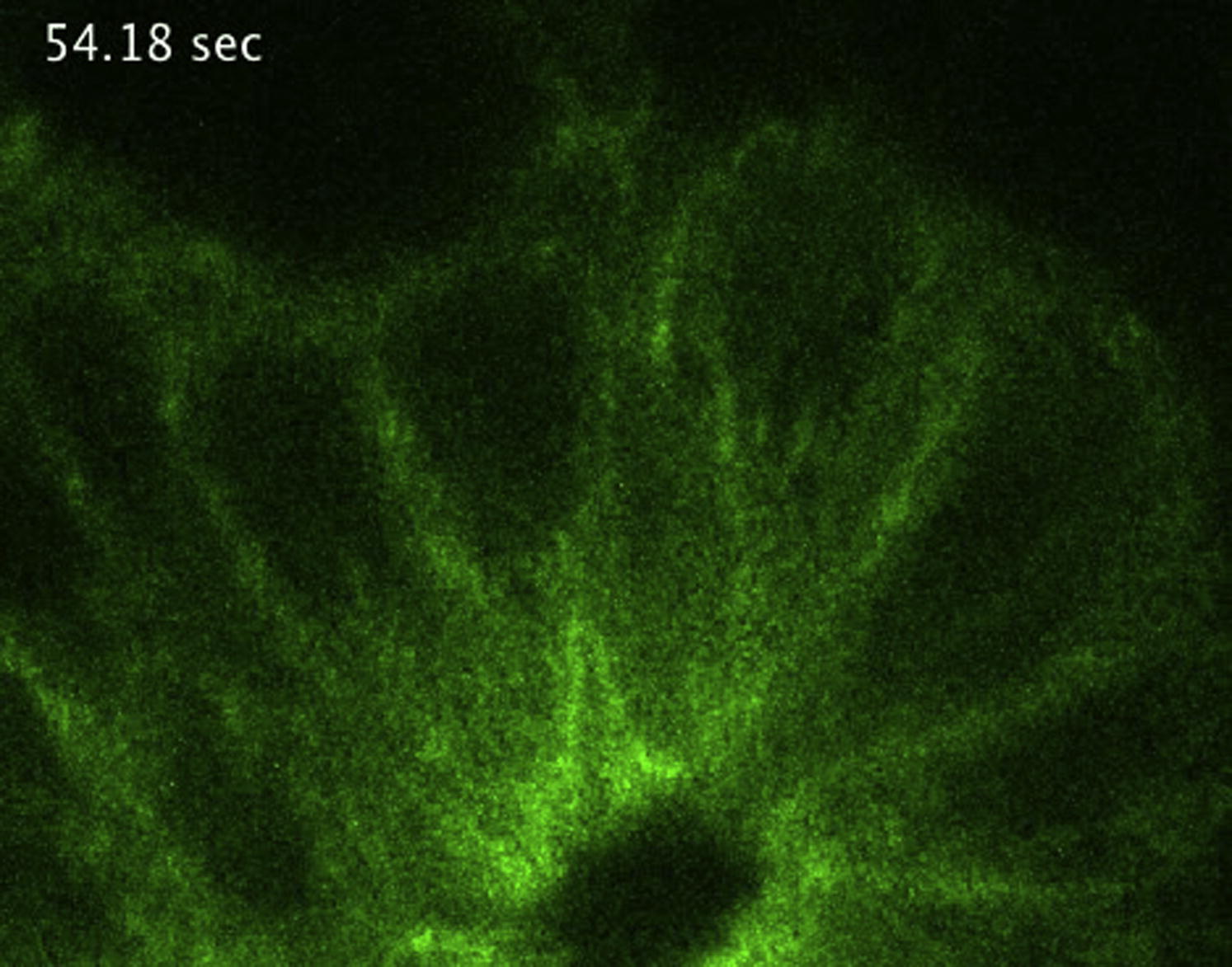

Supplement: Movie S1. MT Dynamics in Invaginating Cells, Related to Figure 1J — Time-lapse analysis of a group of cells undergoing invagination using UAS-GFPClip170 under fkhGal4 control, viewed in a section view, with the apical surface down. Frames are 3.87 s apart; the image shown is 32.5 × 25.5 μm. The green box in frame 1 indicates the position of the kymograph shown in Figure 1J. [file mmc2.jpg]

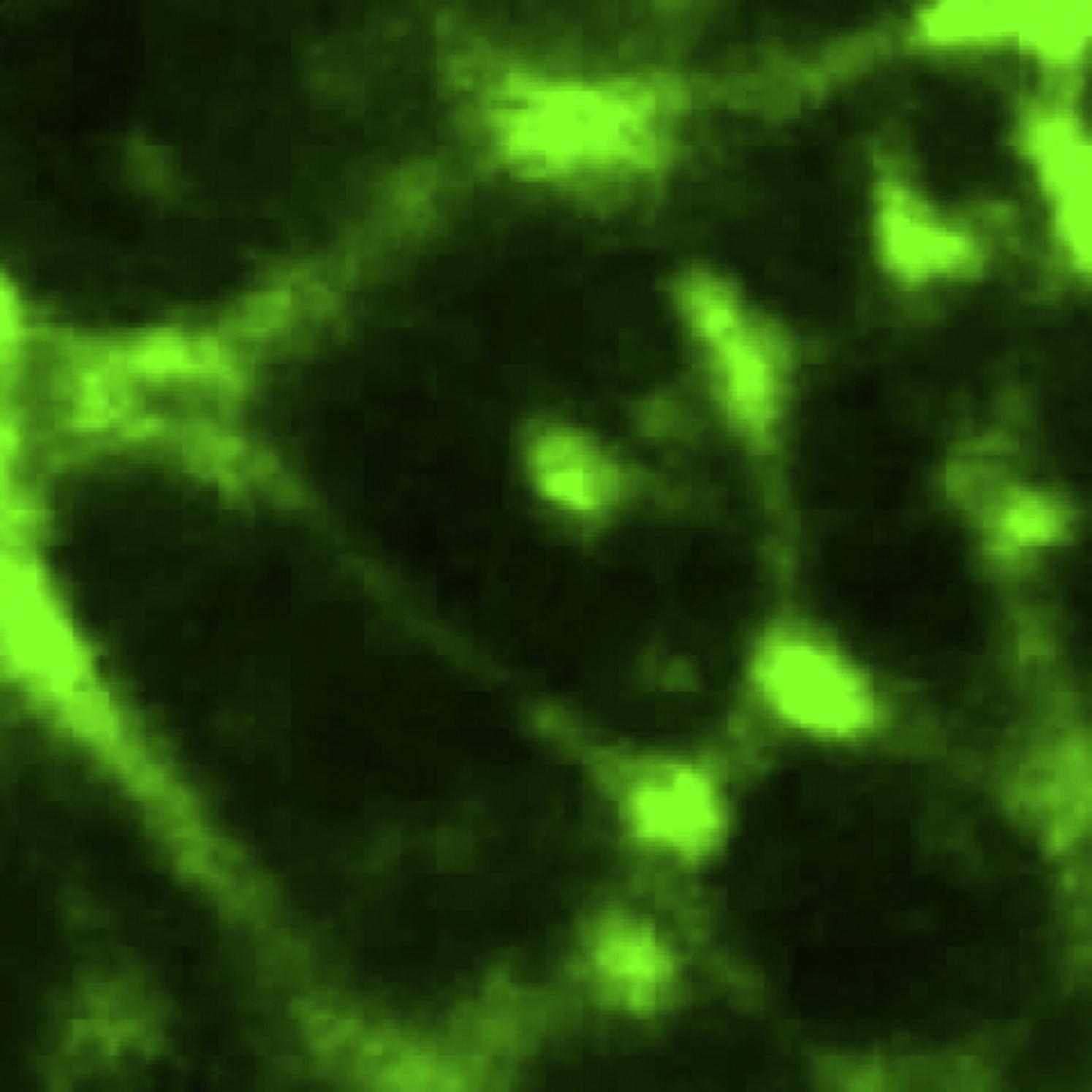

Supplement: Movie S2. Medial Myosin II Fluctuations in a Constricting Placodal Cell, Related to Figures 5B and S4A — Time-lapse analysis of a single cell in the placode showing correlated myosin II fluctuations (visualized by sqhGFP), shown in stills in Figure 5B, and position in gland, shown in Figure S5A. Frames are 18 s apart; the image shown is 7.5 × 7.5 μm. [file mmc3.jpg]

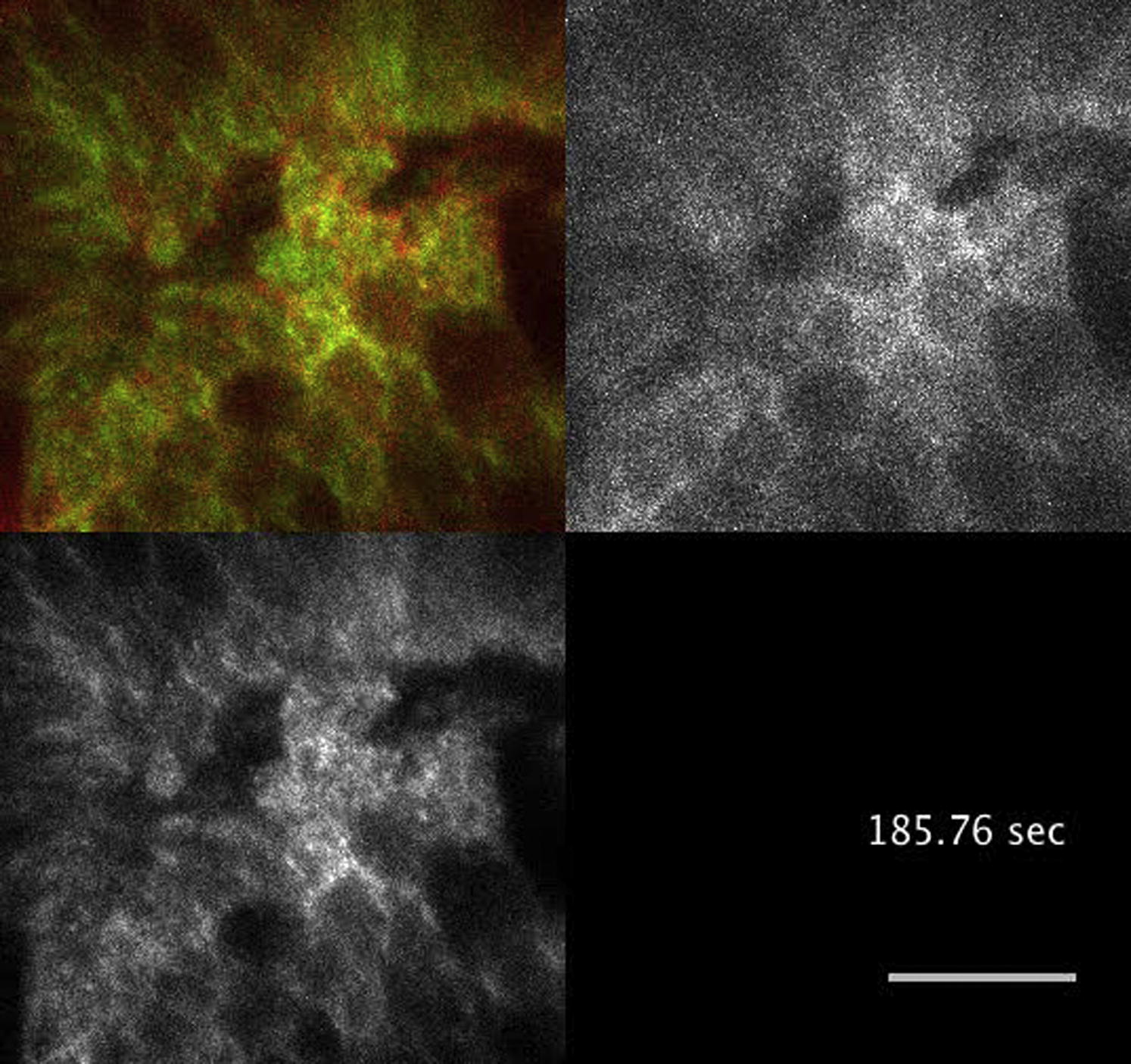

Supplement: Movie S3. Apical Microtubule Bundle Ends and Membrane Marker in a Wild-Type Placode, Related to Figure S4D — Representative time-lapse analysis of a wild-type placode labeled MT using UASGFP-Clip170 under fkhGal4 control (green, bottom panel) and membrane using ECadherin-Tomato (red, right panel). Shown is a single confocal slice grazing the apical surface of the central cells (peripheral cells are seen in section views). Frames are 7.74 s apart; the image shown is 31.8 × 29.9 μm; scale bar is 10 μm. [file mmc4.jpg]

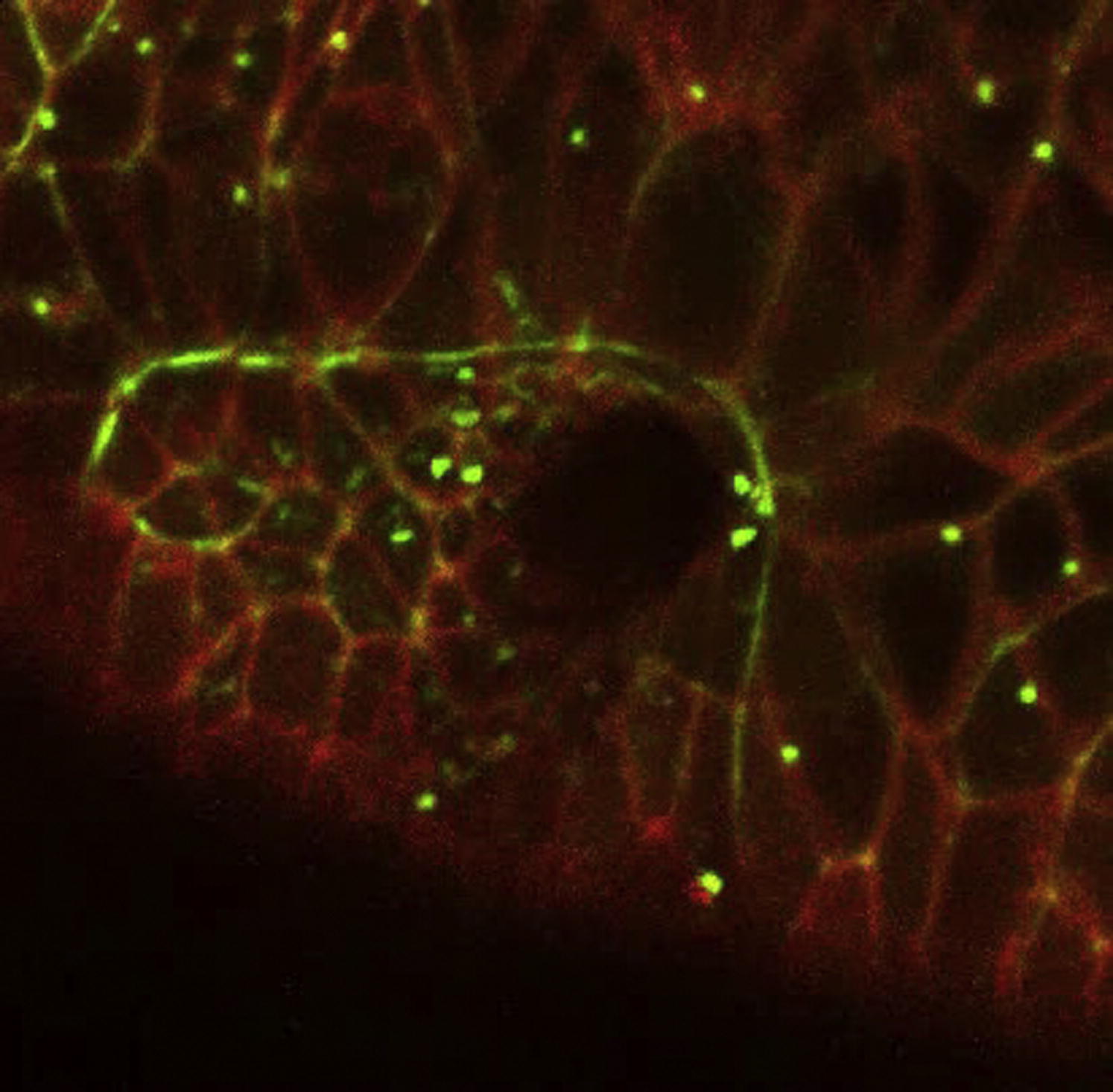

Supplement: Movie S4. Medial Myosin II and Membrane Fluctuations in a Wild-Type Placode, Related to Figures 5E–5K — Representative time-lapse analysis of a wild-type placode labeled using sqhGFP (green) and GAP43mCherry (red). Shown is a maximum-intensity projection of a stack covering the whole surface of the placode; frames are 14.5 s apart; the image shown is 54 × 54 μm. [file mmc5.jpg]

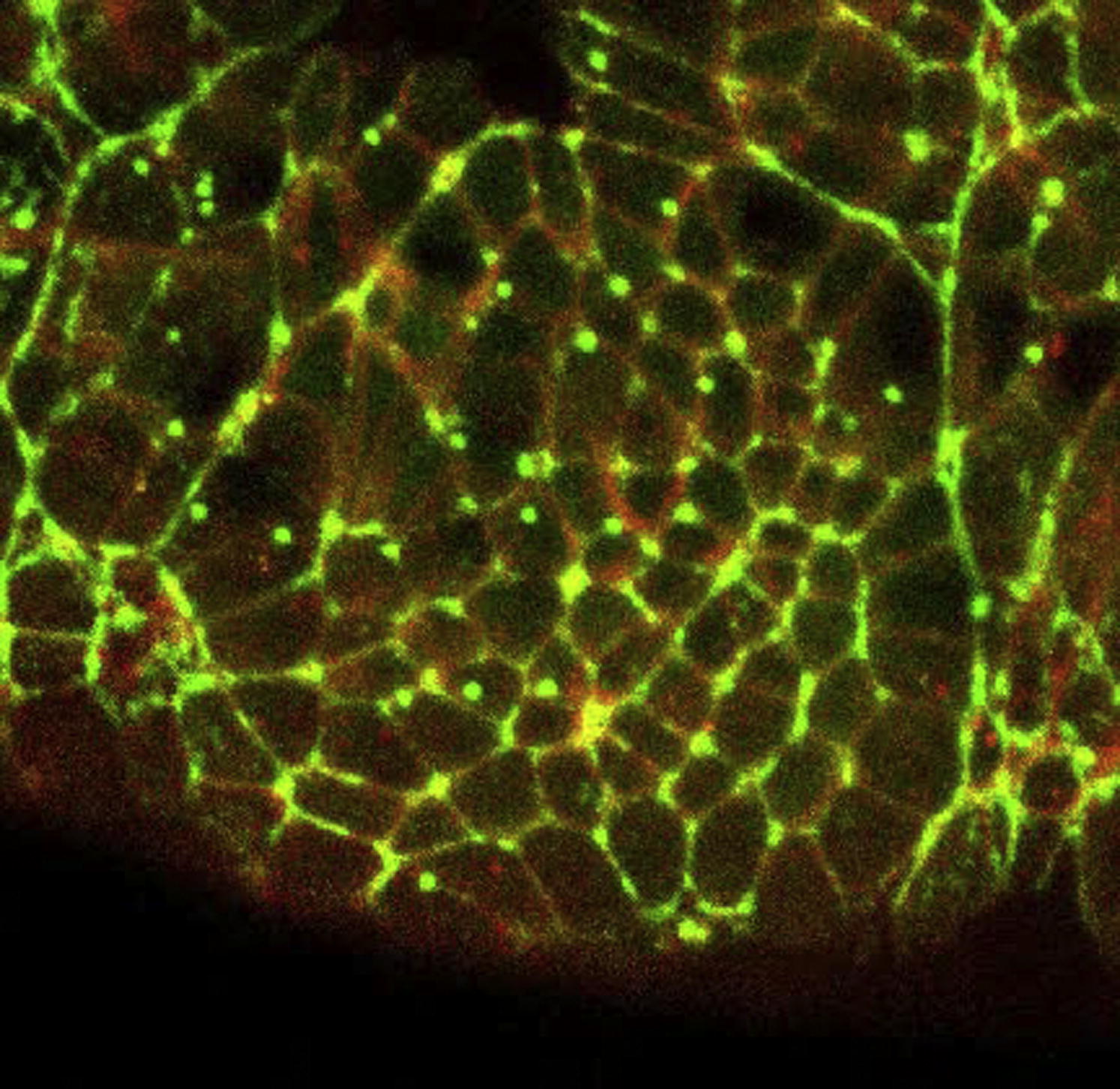

Supplement: Movie S5. Medial Myosin II and Membrane Fluctuations in a Microtubule-Depleted Placode, Related to Figures 5E–5K — Representative time-lapse analysis of an MT-depleted placode (using UAS-Spastin) labeled using sqhGFP (green) and GAP43mCherry (red). Shown is a maximum-intensity projection of a stack covering the whole surface of the placode; frames are 14.5 s apart; the image shown is 59 × 59 μm. [file mmc6.jpg]

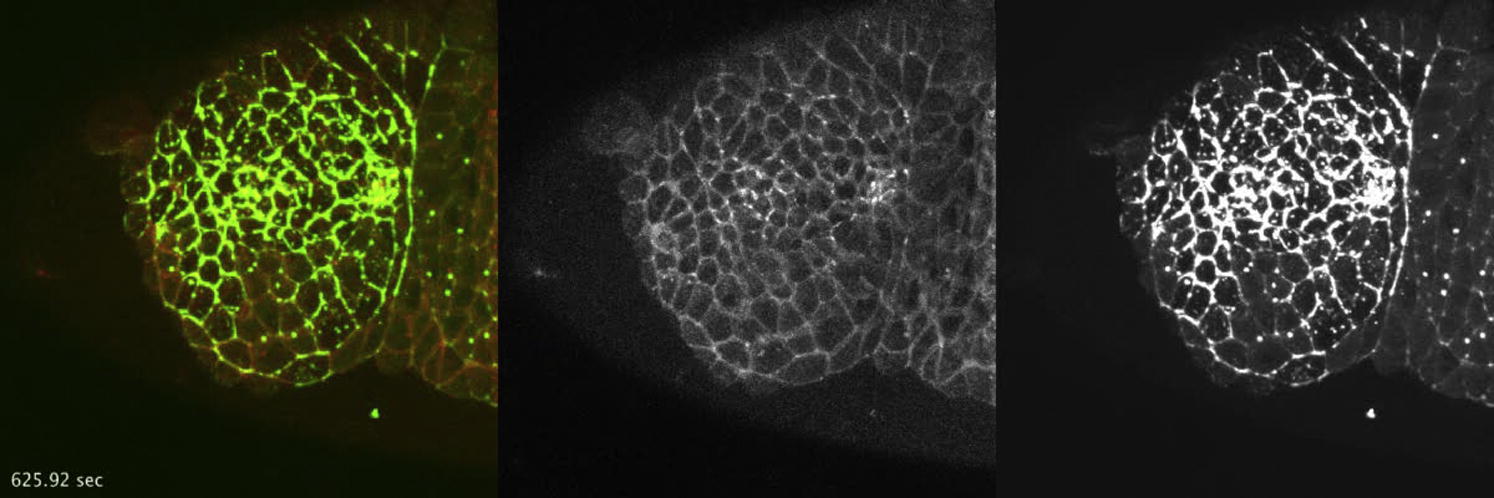

Supplement: Movie S6. Myosin and Membrane Analysis in a Placode with sqhGFP Targeted for Degradation by the Proteasome, Related to Figure S4I — Representative time-lapse analysis of a placode of an embryo of the genotype sqhAX3; sqh::sqhGFP42/UASdeGradFP; fkhGal4/GAP43mCherry, where sqhGFP is targeted for destruction. sqhGFP is in green (right panel); GAP43mCherry is in red (middle panel). Shown is a maximum-intensity projection of a stack covering the whole surface of the placode; frames are 19.56 s apart; the image shown is 85 × 85 μm. [file mmc7.jpg]

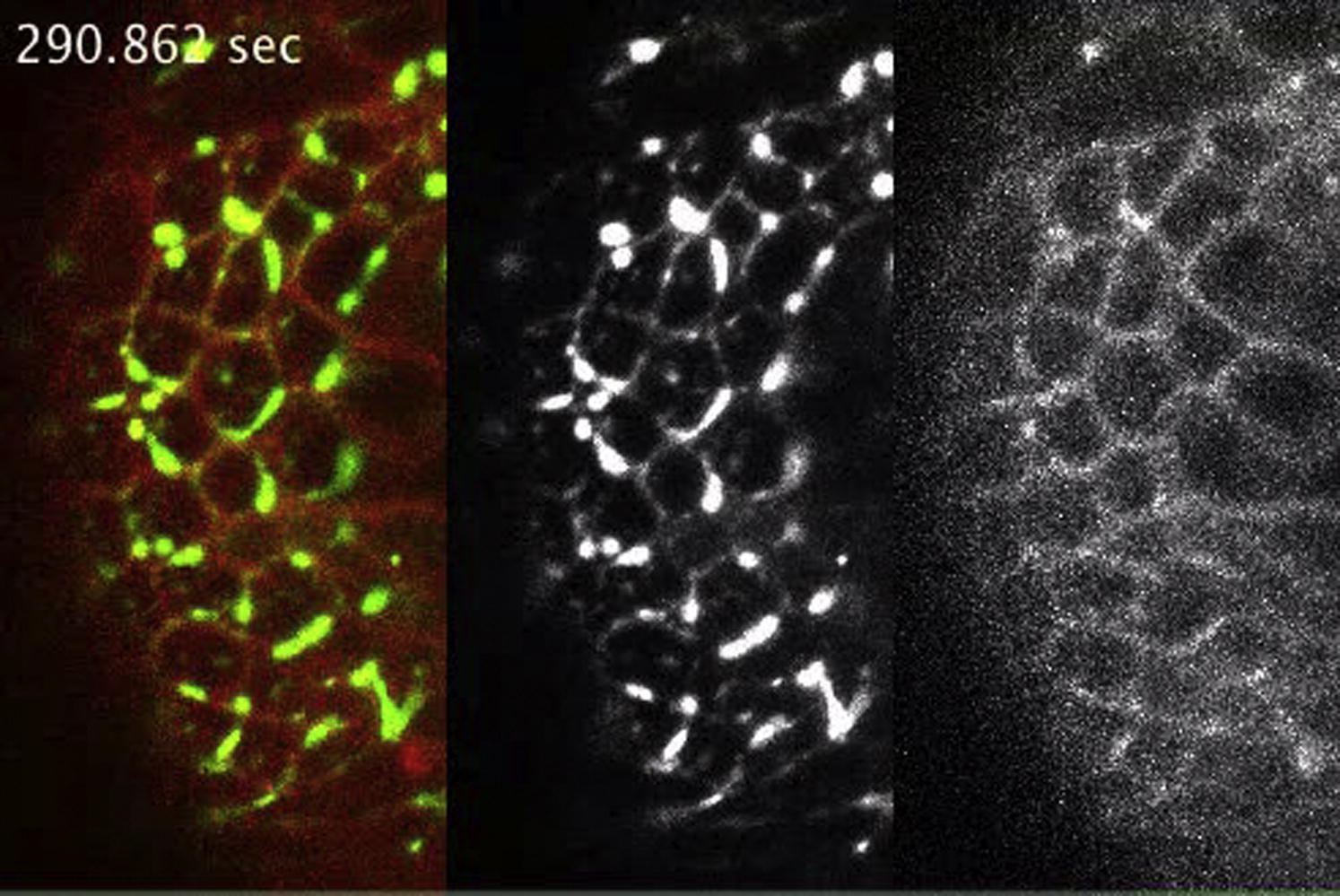

Supplement: Movie S7. Cell-Shape Analysis in a Placode with sqhGFP Targeted for Degradation by the Proteasome, Related to Figure S4J — Representative time-lapse analysis of a placode of an embryo of the genotype sqhAX3; sqh::sqhGFP42/UASdeGradFP; fkhGal4/GAP43mCherry, where sqhGFP is targeted for destruction. sqhGFP is in green (middle panel); GAP43mCherry is in red (right panel). Shown is a maximum-intensity projection of a stack covering part of the surface of the placode; frames are 7.46 s apart; the image shown is 22.7 × 44.8 μm. [file mmc8.jpg]
