# Supplementary material for: A Dynamic Microtubule Cytoskeleton Directs Medial Actomyosin Function during Tube Formation
Source: Dev Cell. 2014 Jun 9;29(5):562–76. doi: 10.1016/j.devcel.2014.03.023 (PMC4064686; doi:10.1016/j.devcel.2014.03.023)
Supplement: Document S2. Article plus Supplemental Information [file mmc9.pdf]

Developmental Cell
Article

A Dynamic Microtubule Cytoskeleton Directs Medial Actomyosin Function during Tube Formation

Alexander J.R. Booth,[1,3] Guy B. Blanchard,[2] Richard J. Adams,[2] and Katja Röper[1,*]
[1]MRC Laboratory of Molecular Biology, Cambridge Biomedical Campus, Francis Crick Avenue, Cambridge CB2 0QH, UK
[2]Department of Physiology, Development and Neuroscience, University of Cambridge, Downing Street, Cambridge CB2 3DY, UK
[3]Present address: College of Life Sciences, University of Dundee, Dow Street, Dundee DD1 5EH, UK
*Correspondence: kroeper@mrc-lmb.cam.ac.uk
http://dx.doi.org/10.1016/j.devcel.2014.03.023
This is an open access article under the CC BY license (http://creativecommons.org/licenses/by/3.0/).

SUMMARY

The cytoskeleton is a major determinant of cell-shape changes that drive the formation of complex tissues during development. Important roles for actomyosin during tissue morphogenesis have been identified, but the role of the microtubule cytoskeleton is less clear. Here, we show that during tubulogenesis of the salivary glands in the fly embryo, the microtubule cytoskeleton undergoes major rearrangements, including a 90° change in alignment relative to the apicobasal axis, loss of centrosomal attachment, and apical stabilization. Disruption of the microtubule cytoskeleton leads to failure of apical constriction in placodal cells fated to invaginate. We show that this failure is due to loss of an apical medial actomyosin network whose pulsatile behavior in wild-type embryos drives the apical constriction of the cells. The medial actomyosin network interacts with the minus ends of acentrosomal microtubule bundles through the cytolinker protein Shot, and disruption of Shot also impairs apical constriction.

INTRODUCTION

Tissue formation during embryogenesis is largely driven by cell-shape changes and cell rearrangements. Cell shape itself is determined intracellularly by the cytoskeleton as well as by cell-extrinsic forces. Over the last decade, the importance of the actin cytoskeleton in the determination of cell shape has been shown for many tissues (Pollard and Cooper, 2009). Actin, with myosin, forms contractile arrays that are key constituents of different morphogenetic processes ranging from epithelial folding to cell intercalation and tissue convergence (Bertet et al., 2004; Martin et al., 2009; Simões et al., 2010). Important functions for different actomyosin structures have emerged, and a distinct population of apical medial actomyosin forming an interlinked network across many cell diameters may be crucial for apical cell constriction and the size of apical cell-cell junctions (Martin et al., 2009; Mason et al., 2013; Rauzi et al., 2010). However, relatively little is known of the roles of microtubules (MTs) during morphogenesis and cell-shape changes.

MTs serve as major tracks for cellular transport, including an important role in membrane uptake and delivery. They are also important for the turnover of adhesion receptors through endo- and exocytosis during cell growth and cell-shape changes (Akhmanova et al., 2009; Mimori-Kiyosue, 2011). However, whereas actin and actomyosin have roles in directly driving cell-shape changes, defined roles for MTs during these processes are scarcer. Examples of roles of MTs in *Drosophila* morphogenesis include roles in cell flattening during amnioserosa elongation (Pope and Harris, 2008), during the zippering stages of dorsal closure (Jankovics and Brunner, 2006), and in the establishment of the correct tracheal branching pattern in embryos (Brodu et al., 2010).

It remains to be elucidated how the actin and MT cytoskeletons interact during cell-shape changes and morphogenesis, even though we know that such crosstalk must be important (Bosher et al., 2003; Hetherington et al., 2011; Lee and Kolodziej, 2002; Röper and Brown, 2003). The clearest example for crosstalk is between aster MTs and the contractile actomyosin ring during cell division (D'Avino et al., 2008; Somers and Saint, 2003; Vale et al., 2009). Additionally, during cell migration and also growth cone steering, close interplay between actin and MTs is important (Basu and Chang, 2007; Broussard et al., 2008; Schaefer et al., 2008).

We have used a model process of tube formation to address the role of the MT cytoskeleton during tissue morphogenesis. The tubes of the salivary gland in the *Drosophila* embryo form from two epithelial placodes through a process of highly coordinated apical cell constriction and invagination (Figure 1A) (Andrew and Ewald, 2010; Myat and Andrew, 2000). Once the cells of the placode have been specified, no further cell division or death occurs here, leaving cell-shape changes and rearrangements as the driving forces of the invagination and making this an ideal system to study how cell-shape changes and rearrangements drive tube invagination. Topologically similar processes of tube formation or budding in mammals can be found during early lung morphogenesis or the elaboration of kidney tubules (Costantini and Kopan, 2010; Warburton et al., 2010). We have previously shown that actomyosin plays an important role during morphogenesis of the glands and identified specific subpools of actomyosin present in the gland placode, in particular a dense junctional and apical medial actomyosin network as well as a circumferential actomyosin cable (Röper, 2012).

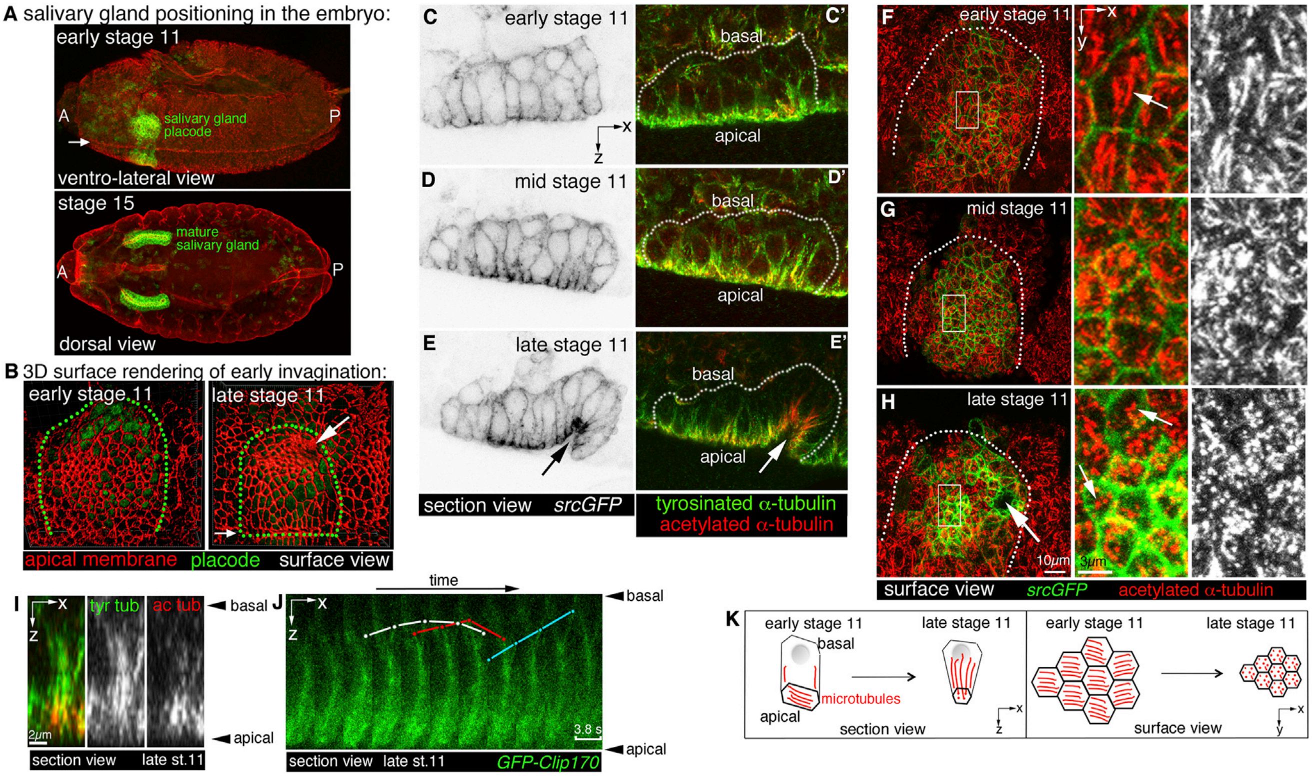

Figure 1. Microtubule Rearrangements during Early Salivary Gland Invagination

(A) Confocal stacks illustrating the position of the salivary gland placode at early stage 11 (top panel; green) and of the fully invaginated glands at stage 15 (lower panel; green). A, anterior; P, posterior.

(B) 3D rendering of apical cortices marked by Crumbs illustrates the early invagination of placodal cells (green), processing from a flat epithelial sheet at early stage 11 (left) to an early invaginated pit at late stage 11 (right). Small arrows in (A) and (B) point to the ventral midline; large arrow in (B) points to the forming pit; green dotted lines in (B) mark the placode area.

(C–E′) Lateral section views of placodal cells at early stage 11 before apical constriction (C), midstage 11 during constriction but before invagination (D), and late stage 11 after initial invagination (E). *srcGFP* (under control of *fkhGal4*) is shown in inverse panels (C)–(E) to outline membranes of placodal cells. Labeling for tyrosinated α-tubulin (green in C′–E′) and acetylated α-tubulin (red in C′–E′) reveals that the number of MTs projecting from the apical surface into the cells increases during early constriction and invagination. The arrows in (E) and (E′) point to the invaginating pit.

(F–H) Surface views of placodes showing that MTs undergo a 90° rearrangement during cell constriction. At early stage 11, labeling for acetylated α-tubulin (red) shows a dense MT network lying parallel to the apical surface of the cells (F). During midstage 11, these MT bundles change orientation (G) to run perpendicular to the apical surface as longitudinal bundles by late stage 11 (H). Constricting apices are marked by *srcGFP* in green. White boxes indicate areas magnified in the center and right panels; small arrows point to apical parallel MTs (F) and the end foci of longitudinal bundles (H); large arrow points to the invaginating pit; white dotted lines mark the placode area.

(I) z section of an MT bundle, with the level of acetylation of MTs being greater nearer the apical surface. Acetylated α-tubulin, red; tyrosinated α-tubulin, green.

(J) Kymograph of a z section time-lapse analysis of microtubule dynamics in an invaginating cell at late stage 11; MTs are visualized using *GFP-Clip170*; frames are 3.87 s apart (see Movie S1). White, blue, and red dots mark individual MTs emanating from the bundle.

(K) Schematic of MT rearrangements in the placode during stage 11.

See also Figure S1 and Movie S1.

We demonstrate that during early steps of tube formation, the placodal MT cytoskeleton undergoes a radical 90° rearrangement with respect to the apicobasal axis, leading to a network of acentrosomal, longitudinal (parallel to the apicobasal axis) MT bundles that abut the apical medial myosin network. Depletion of MTs in the placode leads to a failure of placodal cells to constrict apically, due to a loss of the pulsatile apical medial myosin II network. This medial myosin II network is required to drive contractions of the apical surface of cells in wild-type placodes, and interference with MTs, similar to interfering directly with myosin, affects these dynamic contractions. We show that the cytolinker protein Short Stop (Shot) is localized between apical medial actomyosin and MT (−) ends to mediate a functional interaction between the two networks and that interfering with Shot also impairs apical constriction and tube formation.

RESULTS

Apical Microtubules Rearrange into a Longitudinal Network Concomitant with the Onset of Apical Constriction

To investigate MT organization during salivary gland formation, we used embryos expressing *fkhGal4* and *UAS-srcGFP*, which

highlighted salivary gland cells with membrane-targeted GFP (Maybeck and Röper, 2009). In these embryos, we analyzed the distribution of MTs by labeling tyrosinated α-tubulin, a marker of dynamic or newly polymerized MTs, and acetylated α-tubulin, a marker of stable and longer-lived MTs (Westermann and Weber, 2003). We focused on the early stages of gland formation, where cells prior to invagination begin to elongate along their apicobasal axis and begin to constrict apically (early to midstage 11; Figures 1B–1D), eventually leading to the formation of a dimple that is the early invaginating tube (late stage 11; Figures 1B and 1E).

At early stage 11, MTs in most placodal cells and in the surrounding epidermis were arranged in a dense apical array, lying just underneath the apical plasma membrane, with few MTs extending into the cell interior (Figures 1C′ and 1F). From midstage 11 onward, MTs within the placode changed their major direction of orientation by 90° to align along the apicobasal cell axis (termed longitudinal MTs; Figures 1D′, 1E′, 1G, and 1H). This was particularly obvious in apically grazing sections (Figures 1F–1H and 2A–2C). The rearrangement was tightly coupled in time to the onset of constriction, in that it began in the dorsal-posterior corner of the placode, moving further ventral and anterior as the wave of apical constriction swept across the placode. Reorientation generally appeared to take place prior to apical constriction (Figures S1A–S1B″ available online). Longitudinal MT bundles showed strong acetylation of α-tubulin, a sign of stability, initiating from the apical surface (Figure 1I). Overall, acetylation of MTs was increased within the placode, especially within the highly constricting cells, in comparison to labeling of tyrosinated MTs (Figures S1C–S1D‴). MT bundles labeled by *Clip170-GFP*, a (+) TIP-binding protein (Stramer et al., 2010), that emanated from the apical surface showed dynamic behavior and growth of more basal plus ends (Figure 1J; Movie S1). A striking rearrangement of the MT cytoskeleton thus occurred concomitant with the earliest constriction of placodal cells (Figure 1K).

Longitudinal Microtubule Arrays Emanate from the Apical Region in a Noncentrosomal Manner

Prior to onset of apical constriction and invagination, before midstage 11, most apical MTs were in close contact with apical centrosomes (Figure 2A), suggesting centrosomal nucleation. In contrast, in the rearranged MT network at late stage 11, most apical foci of tyrosinated and acetylated α-tubulin labeling did not colocalize with centrosomes (Figures 2B–2D; data not shown for acetylated α-tubulin). The apical foci of tubulin labeling at stage 11 corresponded to minus ends of MTs, as revealed through analysis of expression of *UAS-Nod-LacZ* and *UAS-Kin-LacZ*, two motor proteins that move toward and thus label the minus and plus ends of MTs, respectively (Figures 2E and 2F) (Clark et al., 1997), consistent with a similar orientation previously observed in fully invaginated gland cells at the end of embryogenesis (Myat and Andrew, 2002). Acentrosomal MTs in postmitotic epithelial cells, such as the placodal cells analyzed here, could be nucleated apically from γ-tubulin complexes not associated with the centrosomes (Bartolini and Gundersen, 2006; Feldman and Priess, 2012; Mogensen, 1999). We thus analyzed γ-tubulin distribution and found a small but significant increase in the amount of apical γ-tubulin that was not associated with the centrosomes labeled by the centrosomal protein asterless after MT rearrangement (Figures 2G and 2H; quantified in Figure 2J; Figures S1E–S1H′), suggesting that this noncentrosomal apical γ-tubulin might be involved in nucleating the longitudinal MTs. In addition, weaker foci of asterless were visible that colocalized with the ends of the rearranged longitudinal MT bundles (Figure 2I, arrows, MTs labeled using acetylated α-tubulin staining), suggesting that both γ-tubulin and asterless could form constituents of this apical noncentrosomal MT-organizing center.

Microtubule Loss Affects Early Apical Constriction in the Placode

In order to analyze whether the MT cytoskeleton in the salivary gland placode was important for early invagination steps, we made use of the MT-severing protein Spastin (Roll-Mecak and Vale, 2008). We overexpressed Spastin specifically in the placode, using *fkhGal4* and *UAS-Spastin*, and analyzed the effect on the MT cytoskeleton and cell behavior. Although the driver appeared not strong enough to affect MTs in all placodes, Spastin expression led to a clear reduction of MTs in 36% of placodes at early stage 11 of embryos of the genotype *fkhGal4 x UAS-Spastin* (n = 105; see also Experimental Procedures; Figure S2). We analyzed cell shapes using E-cadherin labeling of the apical circumference combined with microtubule labeling to identify affected placodes. We segmented apical cell outlines in control and MT-depleted placodes at mid to late stage 11, and from this could calculate both apical surface areas of placodal cells and also the dispersion of cells of different sizes (defined as the average difference in area between each cell and all of its neighbors; see Experimental Procedures). All placodes with a depleted MT cytoskeleton appeared strikingly different from the control (compare Figures 3A and 3B). Whereas in the control, cells began to constrict their apical surfaces in a highly ordered fashion starting from the dorsal-posterior corner (Figures 3A and 3C; Myat and Andrew, 2000), cells in MT-depleted placodes showed significantly fewer constricted apices (Figures 3B, 3D, and 3E). Those cells that did constrict were dispersed throughout the placode, unlike the wild-type cells, where constriction was clustered in the dorsal-posterior corner of the placode, where the invagination of the early tube initiates (Figures 3F–3H). When Spastin-expressing salivary glands were analyzed at stage 14 of embryogenesis, when most of the secretory part of the gland had invaginated in the wild-type (Figure S2F), glands with depleted MTs showed a spectrum of phenotypes consistent with aberrant invagination, from lumen defects to a complete failure to invaginate (Figures S2C–S2E). Similar effects on the MT cytoskeleton and gland invagination were also observed when another MT-severing AAA-ATPase, Katanin (Zhang et al., 2011), was expressed in the salivary gland placode (data not shown).

Thus, the MT organization that we observed concomitant with onset of apical constriction and tissue bending appeared to be an important functional part of the tube formation program.

An Apical Medial Actomyosin Network Is Disrupted in the Absence of Microtubules

What could be the role of longitudinal MTs during the early steps of gland invagination in the wild-type? Apical constriction during

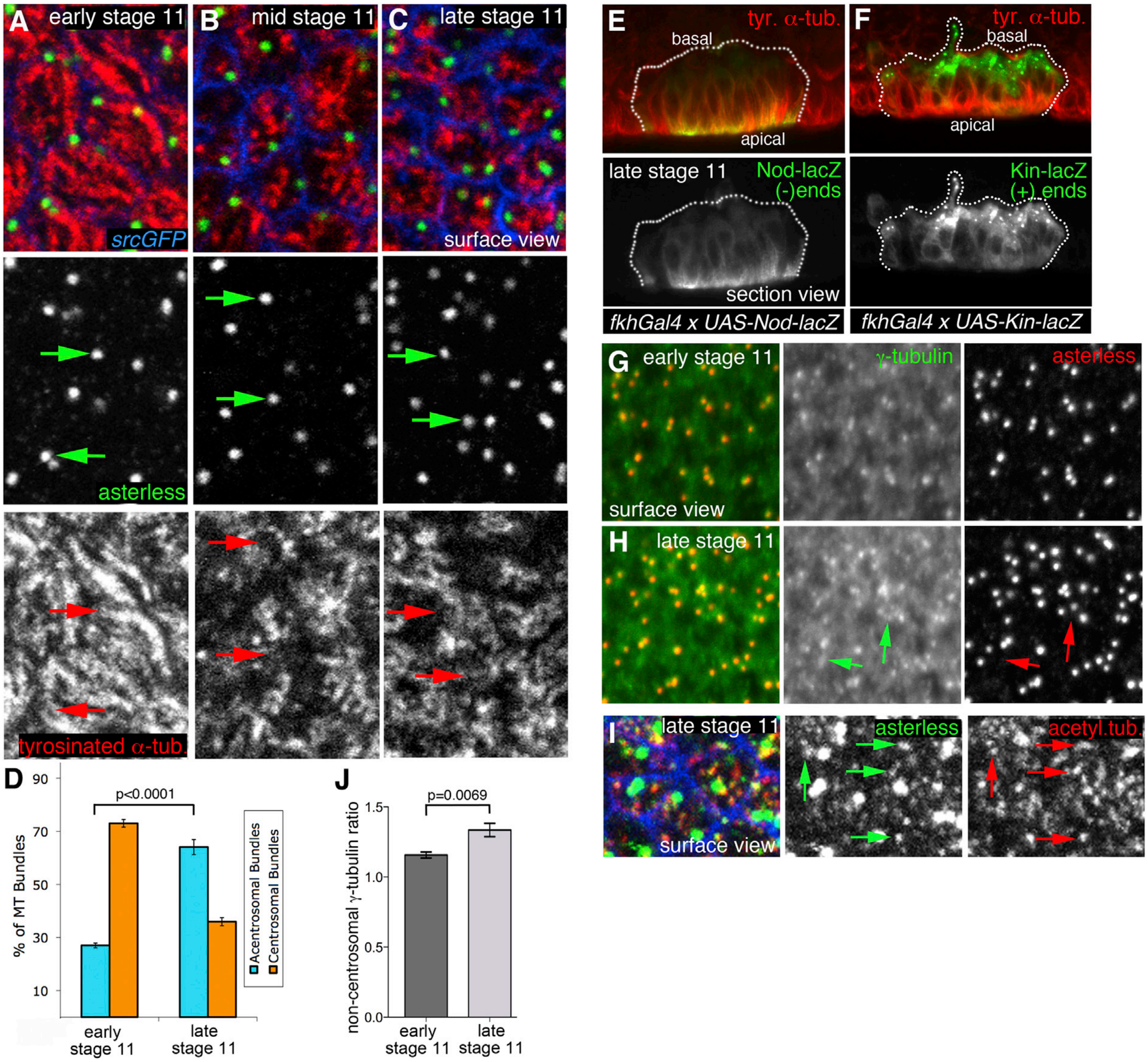

Figure 2. Microtubules Change from Centrosomal to Acentrosomal Nucleation/Anchoring during Early Invagination

(A–C) Surface projections show that at early stage 11, the ends of many apical MTs colocalize with the centrosomal protein asterless (A), but through midstage 11 this changes (B) so that by late stage 11, centrosomes labeled by asterless less frequently colocalize with MT foci (C). Asterless, green; tyrosinated α-tubulin, red; *srcGFP*, blue. Arrows point to centrosomes and the matching positions in the MT channel.

(D) Quantification of colocalization of MT bundle ends and centrosomes at early and late stage 11 (350 MT bundles from six different placodes for each stage; shown are mean ± SEM, $p < 0.0001$ using Student's t test; see Table S1).

(E and F) Section views of late stage 11 placodes: *Nod-LacZ*, a marker of MT (–) ends (E, green), accumulates apically in a flat region of a placode, indicating that MT (–) ends are located apically, whereas an MT (+) end marker, *Kin-LacZ*, is found basally (F, green). Tyrosinated α-tubulin, red. The white dotted lines mark placodal cells.

(G and H) In surface projections of placodal cells, γ-tubulin becomes less tightly centrosome associated from early stage 11 to late stage 11. At early stage 11, the brightest γ-tubulin foci (green) colocalize with centrosomes (red) labeled by asterless (G). At late stage 11, in addition to centrosome foci, further noncentrosomal densities of γ-tubulin labeling have appeared within the placode (H, green arrows), not colocalizing with centrosomes marked by asterless. Panels are higher magnifications of boxes in Figures S1E and S1G.

(I) When asterless labeling (green) at late stage 11 is analyzed at higher laser power, it shows, in addition to strong labeling of centrosomes, many fainter acentrosomal foci that colocalize with apical MT foci (arrows). Acetylated α-tubulin, red; *srcGFP*, blue.

(J) Quantification of the mean noncentrosomal γ-tubulin fluorescence inside versus outside the placode (six placodes were analyzed for each stage; shown are mean ± SEM, $p = 0.0069$ using Student's t test; see Table S1).

See also Figure S1.

epithelial morphogenesis is often mediated by apical actomyosin, located not only at the level of the adherens junctions as an actomyosin belt but also as a medial network of actomyosin underlying the apices of the cells (Blanchard et al., 2010; David et al., 2010; Martin et al., 2009, 2010). The salivary gland placode shows a strong increase in myosin II levels at the start of

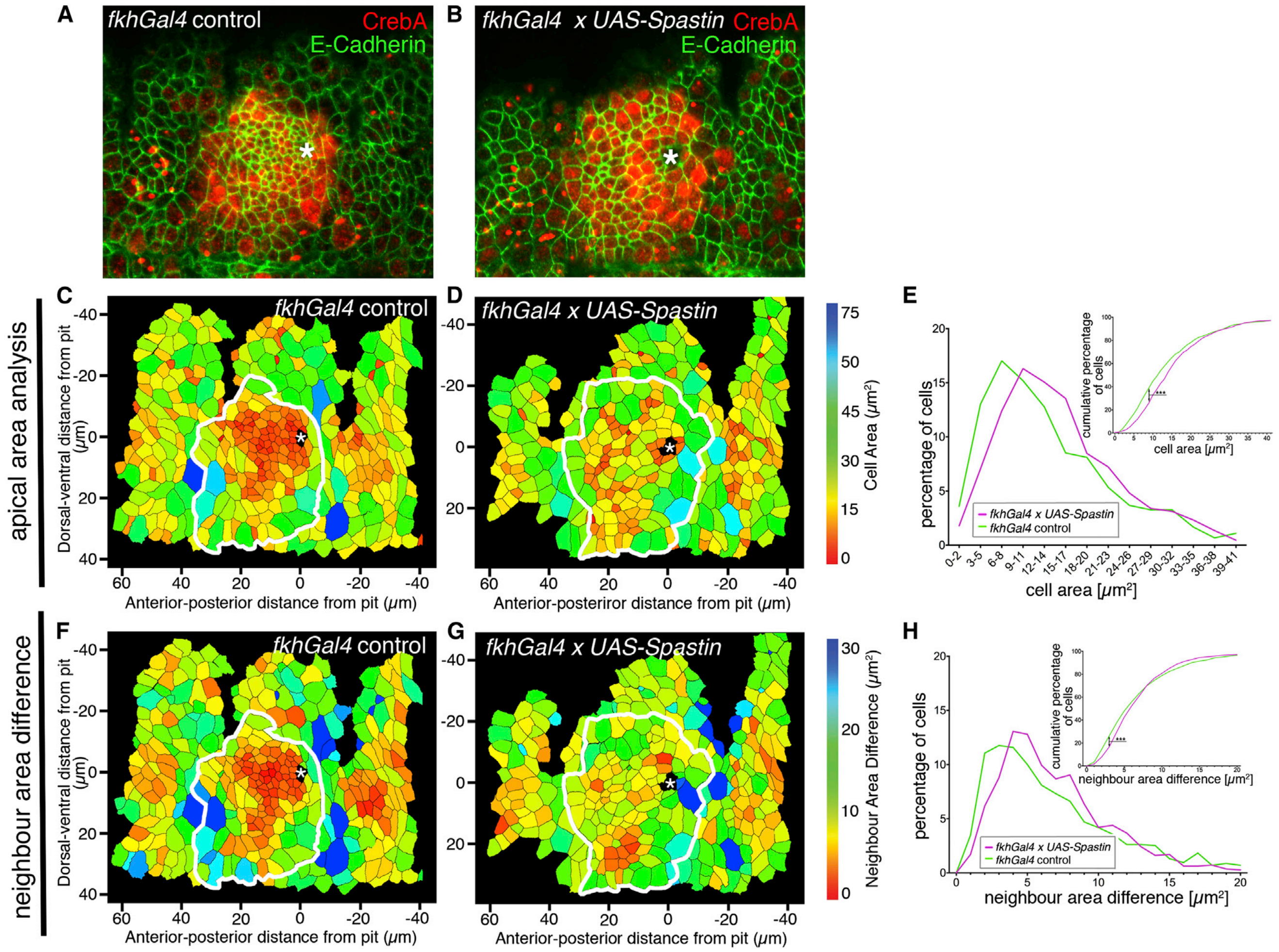

Figure 3. Depletion of the Microtubule Network Disrupts Apical Area Constriction in the Placode

(A and B) The MT cytoskeleton was depleted using expression of *UAS-Spastin* under *fkhGal4* control. Representative surface view images of control (A) and MT-depleted (B) placodes at late stage 11, with E-cadherin (green) labeling cell outlines and CrebA (red) marking the cells of the placode. Asterisks denote the invagination point.

(C and D) Heat maps corresponding to (A) and (B), respectively, indicating apical surface area size determined through automated tracing of E-cadherin-labeled cell boundaries. White lines denote the border of the placode (determined from CrebA labeling).

(E) Quantification of apical area size in MT-depleted (*fkhGal4 x UAS-Spastin*) and control (*fkhGal4*) placodes at late stage 11, showing both the percentage of cells in different-size bins (large graph) and the cumulative percentage of cells relative to apical area size (inset: ***p << 0.001 using Kolmogorov-Smirnov two-sample test; see Table S1). Ten placodes were segmented and analyzed for each condition; the total number of cells traced was N(*fkhGal4*) = 1,198 and N(*fkhGal4 x UAS-Spastin*) = 1,122.

(F and G) Heat maps corresponding to (A) and (B), respectively, indicating the difference in apical surface area size between any given cell and its direct neighbors.

(H) Quantification of (F) and (G) as for apical area differences above (N(*fkhGal4*) = 1,148, N(*fkhGal4 x UAS-Spastin*) = 1,117; inset: ***p << 0.001 using Kolmogorov-Smirnov two-sample test; see Table S1).

See also Figure S2.

morphogenesis, consisting of apical junctional myosin as well as a prominent apical medial myosin II network (Röper, 2012), and myosin II is important for wild-type invagination (Blake et al., 1998). We thus investigated whether the MT cytoskeleton displayed any functional interactions with the apical actomyosin network, using a GFP-tagged transgene of nonmuscle myosin II regulatory light chain (MRLC; termed *sqhGFP* in flies) as a readout. Some medial myosin II could already be observed in all placodal cells prior to constriction starting within the placode, and also in placodal cells farther away from the invaginating pit that had not yet started to contract (Figures S3A and S3C). Very little overlap between apical MT bundles and medial myosin II could be found in these cells (Figures S3B–S3B″ and S3D–S3D″). Once the placodal MTs had undergone their 90° reorientation, however, the apically localized minus ends of longitudinal MT bundles were in close contact and just abutting apical medial myosin accumulations in 90% of cells analyzed (n = 350 cells, six embryos; Figure 4A). This was especially clear in confocal z sections (Figure 4B). The same close apposition could also be observed between minus ends of MTs and apical medial actin

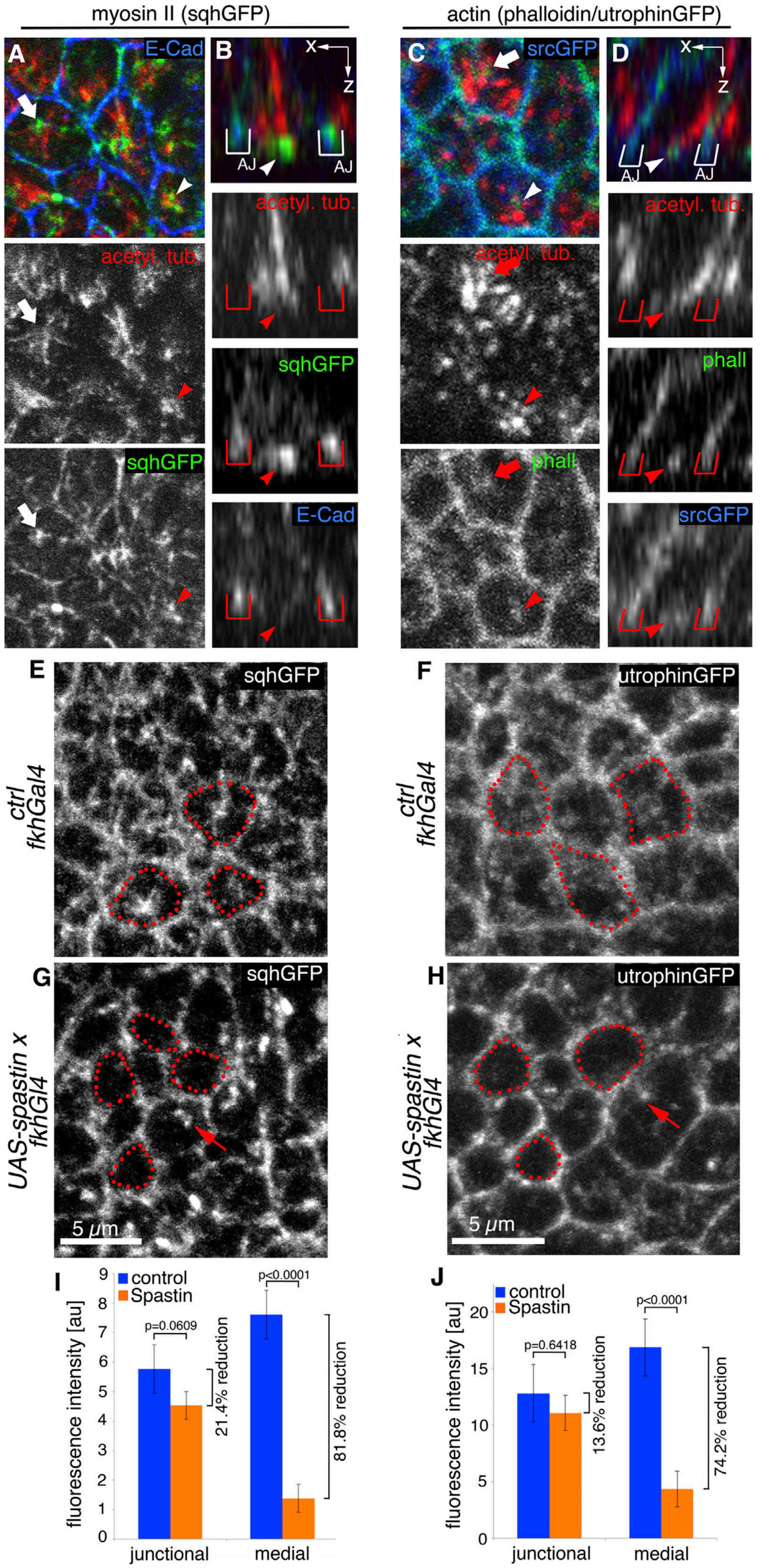

Figure 4. Loss of Microtubules Leads to Loss of the Apical Medial Actomyosin Network

(A–D) Longitudinal MT bundles at late stage 11 terminate apically at foci of medial myosin (surface view, A; z section, B) and F-actin (surface view, C; z section, D). Acetylated α-tubulin, red; *sqhGFP*, green; phalloidin, green; E-cadherin, blue (A and B); *srcGFP*, blue (C and D). The arrows and arrowheads point to colocalization of MTs and medial actomyosin; the arrowheads in (A) and (C) point to the bundle that is displayed in the z sections in (B) and (D). Red brackets indicate positions of adherens junctions (AJ).

(E–H) MT depletion using *UAS-Spastin* and *fkhGal4* disrupts the apical medial actomyosin network. Comparison of *sqhGFP* and *utrophinGFP* (to label actin) in control (E and F) and MT-depleted (G and H) placodes (the panels are higher magnifications of the boxes indicated in Figure S3). Red dotted lines highlight medial apical domains; arrows point to a myosin or actin remnant upon MT depletion.

(I and J) Quantification of the effect of MT depletion on junctional and medial myosin (I), using *sqhGFP*, and actin (J), using *utrophinGFP*. Shown are mean ± SEM of placodal fluorescence intensity above epidermal base level; difference for junctional myosin is $p = 0.0609$ and for medial myosin is $p < 0.0001$, and for junctional actin is $p = 0.6418$ and for medial actin is $p < 0.0001$ using Student's t test (see Table S1).

See also Figure S3.

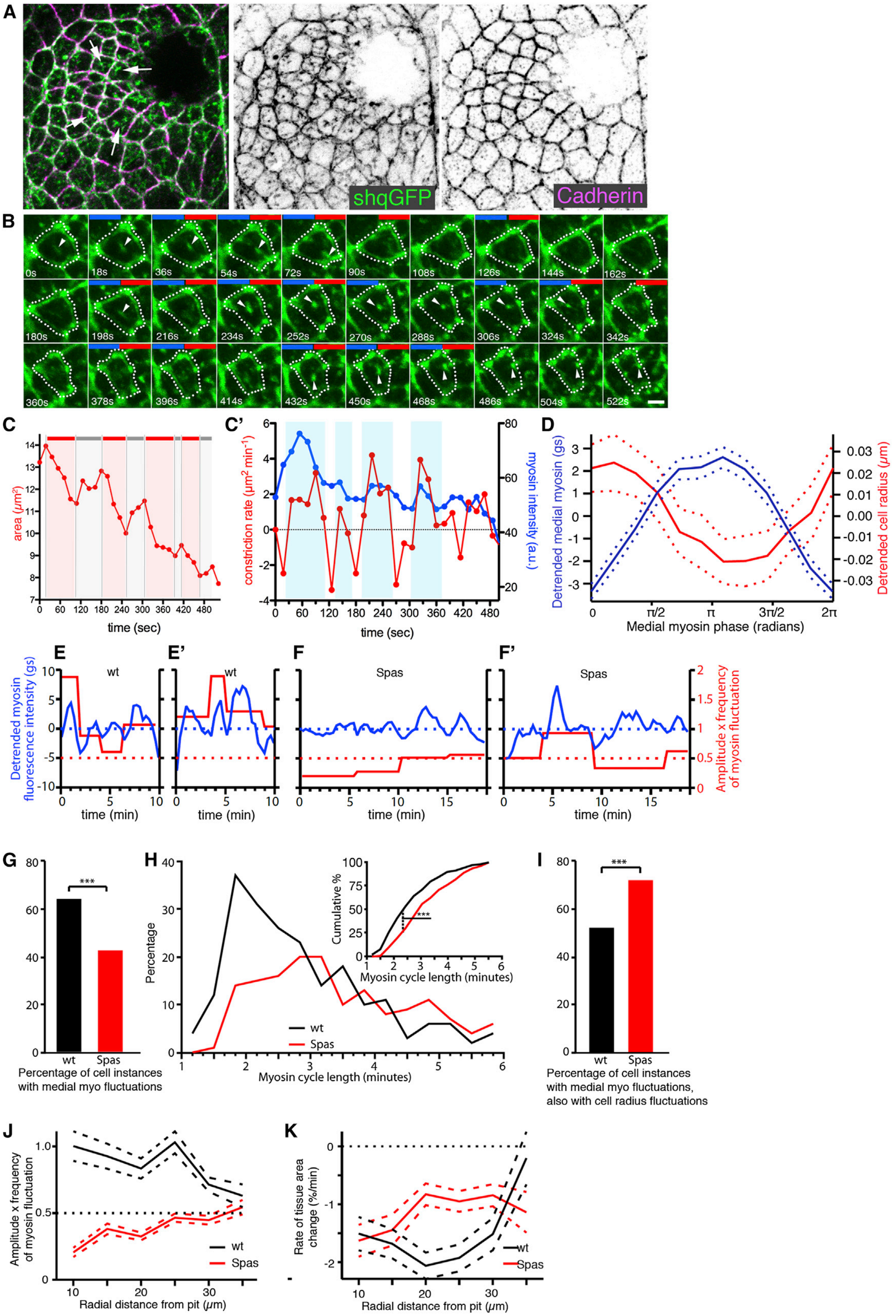

(legend on next page)

(Figures 4C and 4D). No close apposition between junctional actomyosin and MTs could be observed. When MTs were depleted using *UAS-Spastin* and *fkhGal4*, the MT-depleted cells not only displayed larger apical surface areas (as shown above) but also showed a significant decrease in the apical medial pool of myosin, an overall reduction of 81.8% ($p < 0.0001$; Figure 4E versus Figure 4G; quantified in Figure 4I; Figures S3E and S3F). A similar reduction of 74.2% was also observed for the apical medial actin ($p < 0.0001$; Figure 4F versus Figure 4H; quantified in Figure 4J; Figures S3G–S3J). In contrast, the junctional actomyosin located at the level of the adherens junctions was much less affected by MT depletion, with junctional myosin being reduced by only 21.4% ($p = 0.0609$; Figure 4I) and junctional actin decreased by only 13.6% ($p = 0.6418$; Figure 4J).

An Apical Medial Actomyosin Network Drives Apical Constriction during Normal Tube Formation

The apical medial myosin network in placodal cells was highly dynamic and showed pulsatile behavior, characterized by periodic increases and decreases in intensity combined with flows across the apical surface (Figures 5A and 5B; Figures S4A and S4B; Movie S2). In many cells, apical constriction occurred in a stepwise fashion, with pulses of constriction followed by periods of stabilization (example in Figure 5C). The periodic increase in myosin fluorescence intensity in individual cells correlated with an increase in the rate of apical constriction (Figures 5B and 5C′). This suggested that in the salivary gland placode, apical medial myosin was important for apical constriction and tube invagination. Apical MT foci also showed dynamic behavior (Figures S4C–S4D′; Movie S3), reminiscent of medial myosin foci.

To be able to assess the effect that MT depletion had on apical medial actomyosin dynamics and tissue contraction, we simultaneously visualized myosin (using *sqhGFP*) and cell membranes (using *GAP43-mCherry*) in wild-type and MT-depleted embryos. We tracked cell shapes in these movies and quantified medial myosin fluorescence (see Experimental Procedures; Movies S4 and S5). In the wild-type, the phase of average apical medial myosin density was consistently ahead of the phase of the inverse of the apical cell radius by approximately one-eighth of a cycle (Figure 5D; 212 full cycles analyzed from nine embryo movies), suggesting that myosin drove cell-shape changes. We classified each “cell instance” (meaning a movie frame through which a cell was tracked, sampled every ~20 s) as being with or without myosin fluctuation and with or without apical area fluctuation (see Experimental Procedures). Example traces for wild-type and MT-depleted cells show clear differences in myosin fluctuations and strength of myosin activity (Figures 5E–5F′). We used a threshold myosin activity (frequency of fluctuation multiplied by amplitude) of 0.5, above which cells were considered to be fluctuating. This threshold value was set to exclude low-amplitude and/or low-frequency behavior that we would have visually classified as nonperiodic. Pooling cell instances, wild-type placodal cells spent a significantly greater proportion of tracked cell time with measurable myosin fluctuations (64% of tracked cell instances) compared to those in MT-depleted placodes (43%; Figure 5G). Furthermore, the cycle lengths of the myosin fluctuations were also significantly different. Wild-type cells had shorter cycle lengths, with relatively more cycles of less than 3 min in particular (Figure 5H), which in other tissues has been found to be the threshold below which productive

Figure 5. An Apical Medial Actomyosin Network Involved in Apical Constriction during Tubulogenesis

(A) Myosin II (*sqhGFP*, green) is organized into an apical junctional and apical medial network across the salivary gland placode (Röper, 2012). The junctional myosin colocalizes with cadherin (magenta), and the medial myosin forms a network-like arrangement across many neighboring cells (arrows).

(B) Still frames of a time-lapse movie of a *sqh*^{AX3}*; sqh::sqhGFP42* embryo (see Figure S4A; Movie S2). The still frames show the fluctuations of medial myosin and the position of the cell cortex (dotted lines) of an exemplary placodal cell; arrowheads point to dynamic, pulsatile concentrations of myosin; blue bars indicate increased myosin II; red bars indicate increased constriction (see C′). The scale bar represents 2 μm.

(C) Apical cell area (μm^2) decreases in discrete pulses (red bars) followed by a period of relaxation and stabilization (gray bars).

(C′) Quantification of the constriction rate (μm^2 min^{-1}; red) in comparison to medial myosin II intensity (blue) for a single exemplary cell in a placode. An increase in medial myosin II intensity is closely correlated with an increase in constriction rate.

(D) Average medial myosin fluorescence (with trends removed; blue line; gs, grayscale) and cell radius (with trends removed; red line) plotted against phase of medial myosin fluctuation cycle. Two hundred and twelve full cycles of myosin (trough to trough) were pooled and averaged from nine wild-type embryo movies. Dotted lines show 95% confidence intervals.

(E and F) Myosin fluorescence intensity (with trends removed, blue lines) and strength of myosin fluctuation (expressed as amplitude × frequency; red lines) for sample cells. Dotted red lines show the threshold value above which the strength of myosin activity was defined as being periodic.

(E and E′) Two sample wild-type cells (WT).

(F and F′) Two sample MT-depleted cells (Spas). Longer traces are shown for MT-depleted cells because of their longer cycle lengths.

(G–K) Comparison of the average behavior of nine control (wild-type; black) and three MT-depleted embryos (Spas; red). For control embryo data, the number of tracked cell instances (see text) for which it could be established whether a cell was fluctuating or not was 2,877, of which 1,849 exhibited myosin fluctuations. Of the latter, apical radius fluctuated in 929. For MT-depleted embryo data, the number of cell instances was 3,711, 1,584, and 1,106, respectively. See Table S1 for details of statistical analysis.

(G) Percentage of tracked placode cell instances for which medial myosin fluctuations could be detected (see also Movies S3 and S4). ***$p << 0.0001$ using G test of independence.

(H) Distribution of cycle lengths of cells showing myosin fluctuations. Inset: cumulative histograms indicating that cycle lengths of cells still showing fluctuations upon MT depletion were significantly increased. ***$p << 0.0001$ using Kolmogorov-Smirnov test.

(I) Percentage of cell instances with medial myosin fluctuations for which cell-radius fluctuations could also be detected. ***$p << 0.0001$ using G test of independence.

(J) Average strength of myosin fluctuation versus radial coordinate relative to the pit center. Dashed lines are 95% confidence intervals for pooled cell data. Dotted line at 0.5 amplitude × frequency marks the threshold below which cells were not considered to be periodic.

(K) Average rate of change of tissue area versus radial coordinate relative to the pit center (same data as shown in Figures S4E and S4F). Dashed lines show respective 95% confidence intervals.

See also Figures S4 and S5 and Movies S2, S4, and S5.

tissue contraction, as opposed to unproductive area fluctuation, is achieved (Gorfinkiel and Blanchard, 2011). MT-depleted placodes probably retained some myosin fluctuations and apical area constriction due to an incomplete depletion of MTs across all placodal cells due to inhomogeneous *fkhGal4* expression (Figures S2B, S2B′, S3F, S3H, and S3J). Considering only cell instances with measurable myosin fluctuation, the percentage of these cell instances that also displayed area fluctuations was greater in MT-depleted placodes (72% versus 52% in wild-type; Figure 5I), suggesting that in this treatment, contractile myosin pulses were being less effectively harnessed to generate tissue contractile tension, probably a consequence of the increased myosin cycle length. The above differences between MT-depleted and wild-type placodes are the likely cause of the observed defects in apical constriction upon MT depletion.

We expected myosin contractile activity to lead to some combination of net contraction and/or tissue tension. We therefore investigated further the relationship between myosin activity and net contraction. Whereas there was a trend for increasing myosin activity toward the pit in the wild-type (Figure 5J), the strongest rate of apical area decrease, in addition to the early invaginating pit, was observed in a radial band 20–25 μm anterior-ventral to the pit (Figure 5K; Figure S4E). Thus, the pattern of myosin activity did not completely mirror the pattern of net area decrease in wild-type placodes. This was likely due to tissue tension, and hence resistance to contraction, varying in a radial pattern from the pit as the placode started to invaginate to form a 3D structure. In MT-depleted placodes, the rate of area change within the placode was reduced to less than half of wild-type rates within the 20–25 μm radial band, and myosin activity decreased toward the pit and was overall below the threshold set (Figure 5K; Figure S4F). Altogether, these data strongly suggest that in the wild-type, placode tension and constriction were driven to a large extent by an active medial actomyosin network (Figure S4B). MT depletion led to a reduction in both actomyosin fluctuation and fluctuation-driven productive area changes.

To further analyze whether the loss of dynamic apical constrictions of placodal cells upon MT depletion was due to effects on myosin II and not on other factors downstream of MTs, we disrupted myosin II function specifically in placodal cells. In *sqh*-null mutant embryos expressing *sqhGFP* under its endogenous promoter as the only source of functional MRLC (Royou et al., 2004), we specifically targeted *sqhGFP* for destruction by the proteasome only in placodal cells (using *UAS-deGradFP* and *fkhGal4*; see Experimental Procedures for the full genotype). Loss of *sqhGFP* function in placodal cells consistently led to aberrant glands with strong invagination defects at later stages of embryogenesis (Figures S4G–S4H′). Often, cells at stage 13 remained on the surface of the embryo with wide apical surfaces (Figures S4H–S4H″) but still showed a rearranged longitudinal MT network (Figure S4H‴). Time-lapse analysis of a membrane marker in these embryos revealed that cells did not efficiently constrict apically (Figures S4I and S4J; Movies S6 and S7). Thus, myosin II was crucial for the dynamic apical contractility of placodal cells.

To further exclude that the effect on medial actomyosin was an indirect consequence of a general disruption of cells, we analyzed markers of junctional integrity and apicobasal polarity in control placodes and those that had been depleted of MTs. In a wild-type placode at late stage 11, much MT reorientation had occurred and apical constriction had significantly progressed. In contrast, in MT-depleted placodes at late stage 11, about 1–2 hr after the usual start of cell constriction, only reduced apical constriction had occurred as described above. These MT-depleted placodes showed wild-type levels and localization of E-cadherin and Crumbs (Figure S5). This indicates that the loss of apical medial actomyosin and loss of correct apical constriction observed upon MT depletion were not secondary consequences of loss of junctional integrity or apicobasal polarity prior to this stage. Only at later stages (stage 12 and beyond) did MT-depleted placodes show occasional mislocalization of E-cadherin and Crumbs (Figures S5F, S5G, S5I, S5O, S5P, and S5R), possibly due to effects of prolonged MT depletion on either vesicle delivery or recycling.

Thus, the constriction of apical surfaces that initiated the invagination of the epithelial sheet depended on a dynamic apical medial actomyosin network, which in turn depended on the MT cytoskeleton for its assembly and/or maintenance.

The Spectraplakin Shot Functionally Bridges Apical MT Ends and Medial Actomyosin

What links the apical actomyosin and MT networks? The spectraplakin family of cytolinkers, containing both actin- and MT-binding domains, is a prime candidate to mediate such interaction (Röper et al., 2002; Suozzi et al., 2012). The sole fly spectraplakin is Shot, a large protein containing two N-terminal actin-binding calponin homology (CH) domains and an MT-binding Gas2 domain and C terminus (Figure S6A) (Röper et al., 2002). Shot was strongly expressed within the salivary gland placode during early invagination (Figures 6A and 6E for an overview). Shot was localized in a mostly cortical position at the apical surface of placodal cells at early stage 11, similar to its localization within the surrounding epidermal cells (Figures 6A and 6E). However, from midstage 11 onward, Shot lost much of its cortical concentration within the placode, appearing instead in large apical foci that often colocalized with the apical MT foci (Figures 6B–6E). In fact, 83% of MT bundles terminated in foci of Shot labeling at late stage 11 (Figure S6F). Shot colocalization with apical MT minus ends depended on the Gas2 domain and C terminus but not on its actin-binding CH domains (Figures S6C–S6E′). Shot foci also colocalized with the highest accumulation of apical medial myosin (Figures 7A and 7B), thus making it a prime candidate to bridge longitudinal MT bundles and apical medial actomyosin. MT depletion using UAS-Spastin blocked the Shot rearrangement: Shot remained localized to cortices and did not become concentrated in apical foci (Figure 7C versus Figure 7D; Figure S6G).

Shot is essential for oogenesis and egg formation (Röper and Brown, 2004), and therefore embryos lacking both zygotic and maternal pools of Shot during embryogenesis cannot be generated and analyzed. In embryos zygotically mutant for the null allele *shot*^{3}, we could still observe residual protein and could not identify any phenotype in gland invagination (data not shown). To interfere with Shot function, we expressed the EF-hand and Gas2 domain of Shot (Figure S6A, green line), fused to GFP under UAS control (*UAS-Shot-EFGas2*; Maybeck and Röper, 2009) using a strong maternal driver, *nanosGal4VP16*,

CellPress

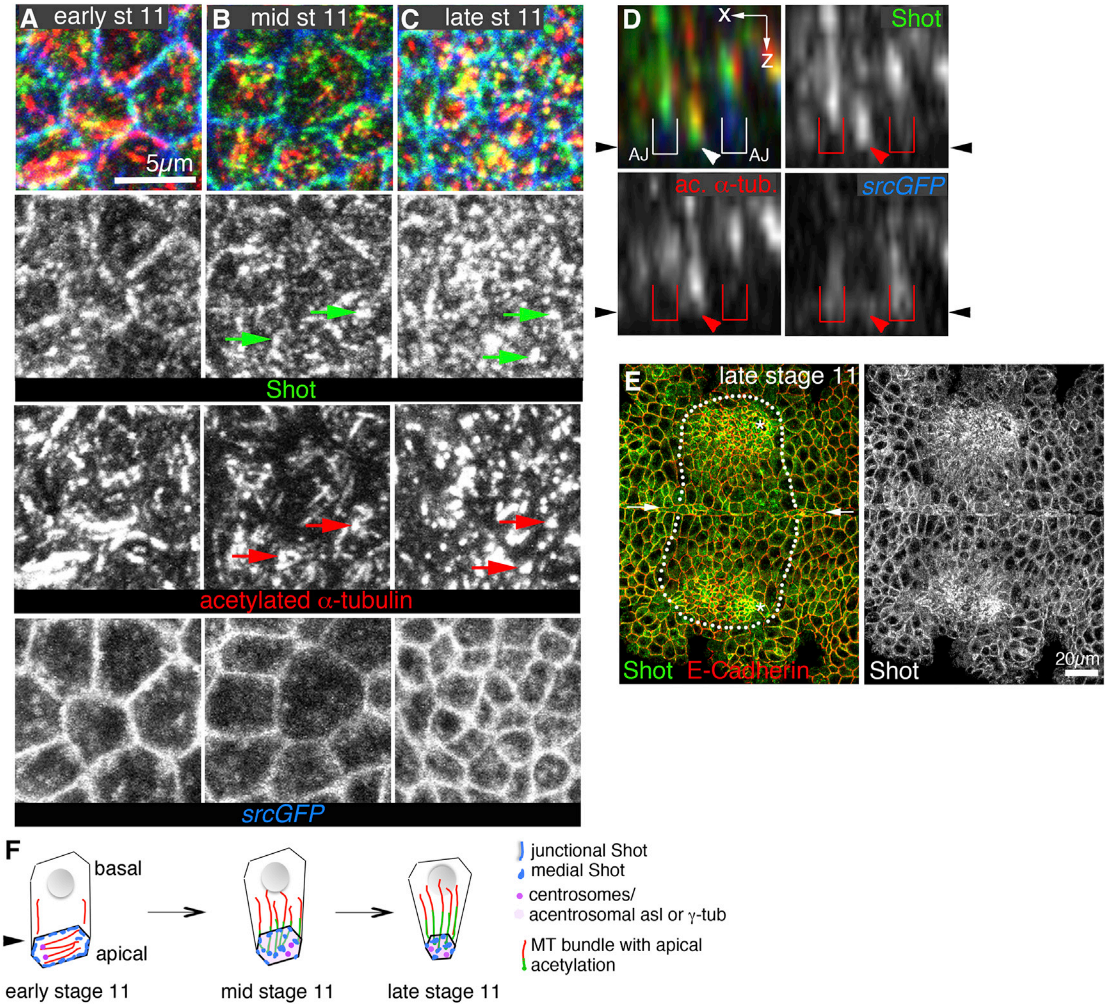

Figure 6. The Cytolinker Shot Relocalizes from the Cell Junctions to the Apical Ends of Microtubule Bundles during Early Invagination

(A–C) The spectraplakin Shot contains actin-binding and MT-binding domains (see Figure S6A). At early stage 11, the majority of Shot (green) localizes to the cell cortex as described in other epithelia (A) (Röper and Brown, 2003), but during midstage 11, Shot relocalizes (B) to then colocalize with the ends of longitudinal MT bundles (red) by late stage 11 (C); shown are surface views. Arrows in (B) and (C) point to colocalization between Shot and MT foci.

(D) z sections show Shot (green) localized at the end of an MT bundle (red). Eighty-three percent of MT bundles terminate in an apical focus of Shot (see Figure S6F). The arrowheads indicate the end of a microtubule bundle; the brackets indicate the positions of adherens junctions.

(E) Overview surface scan at late stage 11 clearly shows the change in Shot localization (green) within the constricting secretory part of the placodes (marked by white dotted lines) compared to junctional Shot outside that placode that colocalizes with E-cadherin (red). Small arrows indicate the ventral midline.

(F) Schematic of coordinated MT and Shot reorganization during early constriction. Black arrowheads in (D) and (F) indicate position of the apical domain.

See also Figure S6.

combined with *fkhGal4*. When *UAS-Shot-EFGas2* was expressed using *fkhGal4* alone, no perturbation of gland invagination could be observed (Maybeck and Röper, 2009). In combination with a strong maternal expression, placodal expression of this transgene was able to cause aberrant invagination with variable penetrance, with many invaginating cells not constricting in a wild-type pattern (Figures 7E, 7G, 7I, and 7K; exemplary area heat maps in Figures 7O and 7P; quantification in Figure 7Q; Figures S7H and S7I). Overexpression of *GFP-Shot-EFGas2* did not seem to affect MT rearrangement into longitudinal bundles (Figure 7G versus Figure 7H), but instead appeared to have a dominant-negative effect on endogenous Shot. Much of endogenous Shot remained cortically localized and did not move into medial apical foci (Figure 7I versus Figure 7J and insets; quantification in Figure 7M). Medial F-actin accumulation as a readout for apical medial actomyosin was reduced in a similar manner (Figure 7K versus Figure 7L; quantification in Figure 7N).

Thus, Shot localization to the minus ends of MT bundles within the apical medial domain, where it colocalized with the apical medial myosin network, was important for normal apical constriction during tube formation.

DISCUSSION

Our analysis shows that MTs play a crucial part in stabilizing and maintaining the medial actomyosin network driving the formation

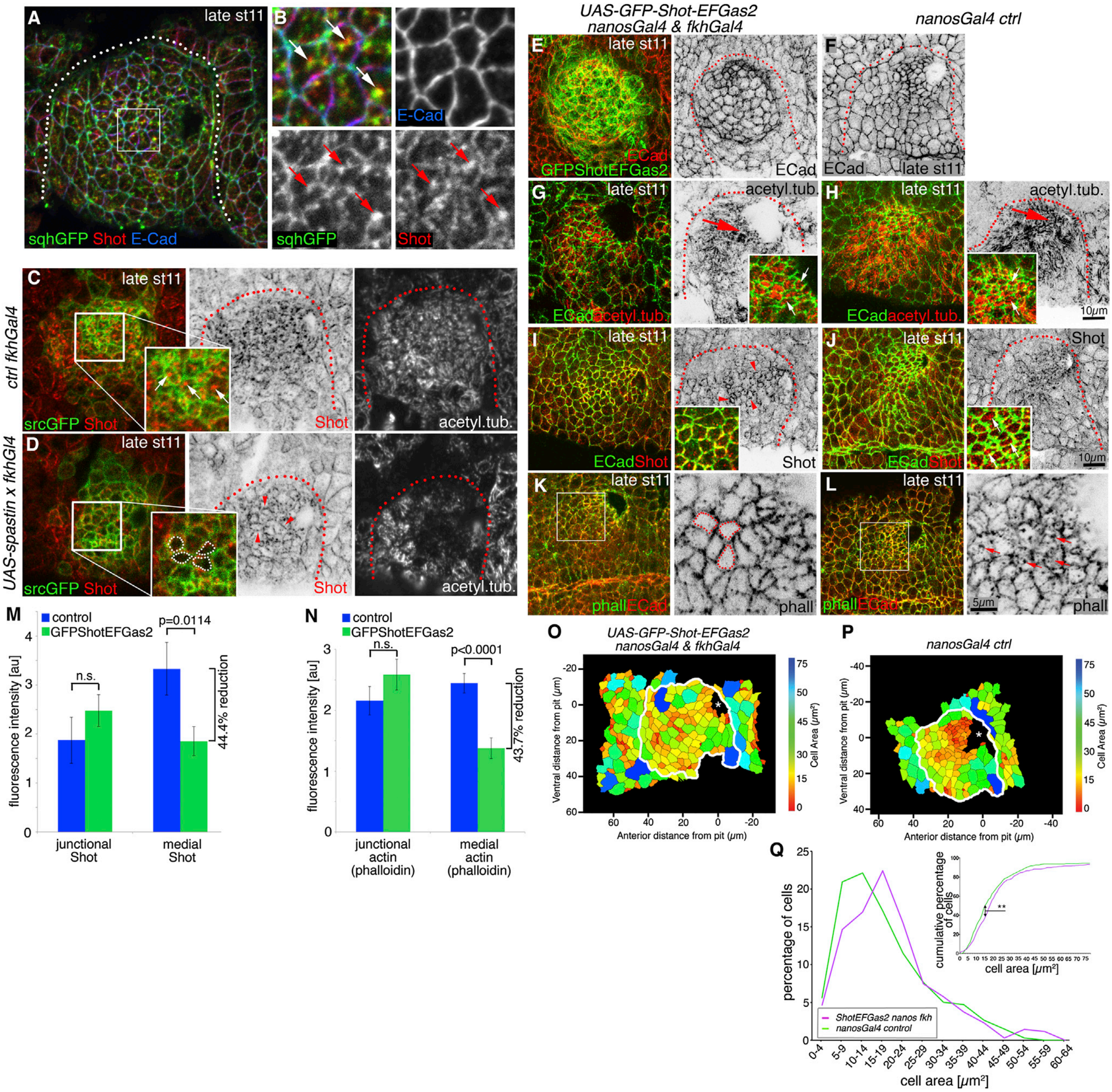

Figure 7. Shot Links Microtubules to Medial Actomyosin, and Medial Shot Is Required for Apical Constriction

(A and B) At late stage 11, medial Shot (red) colocalizes with medial myosin (*sqhGFP*, green). The area marked by the white box in (A) is shown enlarged in (B). Arrows point to *sqhGFP*-Shot colocalization.

(C and D) Depletion of MTs in the placode leads to a failure to relocalize Shot from the junctions to the apical medial region of placodal cells. In contrast to a control placode (C), where Shot (red) is localized to the apical medial region (see arrows in the inset), when MTs (acetylated α-tubulin) are depleted using *UAS-Spastin* and *fkhGal4* (D), Shot remains associated with the junctional area (green, *srcGFP*) in the placodal cells (inset: dotted lines mark medial regions of cells; arrowheads point to junctional Shot; quantified in Figure S7G).

(E and F) Overexpression of *GFP-Shot-EFGas2* using *nanosGal4* and *fkhGal4* interferes with apical constriction (E) compared to the control (F). E-cadherin, red and as a single channel in (E) and (F); *GFP-Shot-EFGas2*, green in (E).

(F) The single E-cadherin channel of the panel shown as a composite in (L).

(G and H) MTs rearrange and appear stabilized when *GFP-Shot-EFGas2* is overexpressed (G) as in the control (H). Insets: MT bundle ends marked by acetylated α-tubulin (red; arrows) in between cell cortices marked by E-cadherin (green).

(I and J) Endogenous Shot (red) remains cortical when *GFP-Shot-EFGas2* is overexpressed (I; red arrowheads) in contrast to the control, where Shot relocalizes to medial MT ends (J). Insets: Shot colocalizing with junctional E-cadherin (green) upon *GFP-Shot-EFGas2* expression (I), in contrast to medial Shot accumulations in the control (J; arrows). Shot levels are quantified in (M).

(legend continued on next page)

of a tube in fly embryos. We observe a very dynamic medial network of actomyosin that shows a pulsatile increase in intensity correlated with a decrease in apical surface area of individual cells. Such pulsatile actomyosin behavior is similar to what has been observed in the fly mesoderm prior to its invagination (Martin et al., 2009, 2010; Mason et al., 2013) and in the constricting flat sheet of amnioserosa cells during dorsal closure in the fly embryo (Blanchard et al., 2010). The average cycle length of myosin oscillations observed in the salivary gland placode of ~120–180 s is comparable to the cycle length of ~147 s (±43.5 s) observed for medial actomyosin oscillations during germband extension (Fernandez-Gonzalez and Zallen, 2011; Sawyer et al., 2011), whereas the faster process of mesoderm bending and internalization has a higher frequency of 82.8 s (±48 s) (Martin et al., 2009), and amnioserosa oscillations reduce from more than 4 min to 2 min as net tissue contraction commences during dorsal closure (Gorfinkiel and Blanchard, 2011; Gorfinkiel et al., 2009; Solon et al., 2009). The disruption of MTs using Spastin expression in the salivary gland placode decreases the proportion of time that cells show myosin oscillations, and strongly decreases the frequency of oscillation. In the absence of MTs, the majority of oscillations were in a time regime where they were unlikely to drive productive apical area decrease, consistent with previous results (Gorfinkiel and Blanchard, 2011).

There have been some previous hints that interplay between the MT cytoskeleton and actomyosin might also regulate morphogenesis in vertebrates. During *Xenopus* neurulation, the protein Shroom3 is necessary for the reorganization and accumulation of apical γ-tubulin and the assembly of an apical array of MTs (Lee et al., 2007). Shroom proteins, including Shroom3, bind Rho kinase to activate contractile actomyosin networks (Mohan et al., 2012). Thus, during cell-shape changes in the forming *Xenopus* neural tube, Shroom appears to be a potential linker between MTs and actomyosin. A *Drosophila* Shroom protein has only recently been identified, and so far mutant analysis suggests a role in recruitment of cortical myosin (Bolinger et al., 2010; Simões et al., 2014). A topologically related process in *Xenopus*, the apical constriction of bottle cells during gastrulation, depends on both intact actomyosin and microtubule networks, revealed when constriction upon treatment with specific chemical inhibitors was analyzed (Lee and Harland, 2007). Furthermore, tube formation in mammals, where live analysis is technically very challenging, also depends on apical constriction (Bush et al., 1990). Thus, data obtained on tubulogenesis in more accessible models will be a valuable guide to further studies in mammals.

Concomitant with medial actomyosin-driven apical constriction, the MT cytoskeleton organized into a noncentrosomal stabilized array combined with a topological rearrangement. MTs in most epithelia are acentrosomally nucleated, a feature conserved from flies to humans (Bartolini and Gundersen, 2006). Our analyses, and further examples from *Drosophila*, highlight the importance of a reorganization or changes in stability of the MT cytoskeleton to permit and/or drive morphogenetic processes: during dorsal closure, the MT cytoskeleton forms an apical parallel array important for zippering (Jankovics and Brunner, 2006); during cell flattening and elongation of the early amnioserosa cells, the MT cytoskeleton undergoes a perpendicular rotation to drive the observed "rotary cell elongation" (Pope and Harris, 2008); during formation of the tracheal system, noncentrosomal microtubules are formed that are important for the establishment of the correct branching pattern (Brodu et al., 2010); and during morphogenetic furrow progression in the eye, imaginal disc MTs appear apically stabilized (Corrigall et al., 2007). Thus, dynamic MT arrays susceptible to reorganization appear to form an important basis of several morphogenetic processes.

We show that the cytolinker Shot is the likely linker between the longitudinal MT bundles and the medial actomyosin network. When the relocalization of Shot to the medial apical domain during stage 11 is reduced, placodal cells often fail to invaginate properly, apical constriction is affected, and medial F-actin (likely together with medial myosin) is reduced. In its medial position, Shot could provide a physical coupling between MT bundles and apical actomyosin (MT bundle ends themselves show dynamic behavior within the apical domain, similar to myosin), but Shot could also recruit further factors crucial for actomyosin network assembly and function. The nature of these remains to be determined. The fact that the rearranged MT bundles show acetylation marks near the apical surface, suggesting their stabilization over time, also indicates that Shot might provide a stable, albeit not necessarily static, link between microtubule ends and apical medial actin.

Apical constriction based on medial myosin fluctuations is one of several important mechanisms that seem to operate to ensure proper tube invagination in the salivary glands, suggesting a complex "belt-and-braces" arrangement. We have shown previously that a multicellular actomyosin cable at the boundary of

Dotted lines in (A)–(J) mark the area of the placode, unless indicated otherwise.

(K and L) Placodes overexpressing *GFP-Shot-EFGas2* often show reduced medial F-actin (K) compared to controls (L). Phalloidin (labeling F-actin; red in K and L and as a single channel in inverse panels); E-cadherin, green; white boxes mark areas magnified in inverse panels; dotted lines mark cells with no medial F-actin; arrows point to medial F-actin in the control. F-actin is quantified in (N).

(M and N) Quantification of the effect of *GFP-Shot-EFGas2* overexpression on junctional and medial Shot (M) and actin (N; using phalloidin). Shown are mean ± SEM of placodal fluorescence intensity above epidermal base level; difference for junctional Shot is $p = 0.2932$ and for medial Shot is $p = 0.0114$, and for junctional actin is $p = 0.2134$ and for medial actin is $p < 0.0001$, using Student's t test; n.s., nonsignificant (see Table S1).

(O and P) Exemplary heat maps indicating the apical surface area size of *UAS-GFP-Shot-EFGas2*-expressing embryos (*nanosGal4* and *fkhGal4* control; M) and of control embryos (N) determined through automated segmentation of E-cadherin-labeled cell boundaries. The white lines denote the border of the placode. Asterisks denote the invagination point.

(Q) Quantification of the apical area size in *GFP-Shot-EFGas2*-expressing (using *fkhGal4* and *nanosGal4VP16*) and control (*nanosGal4VP16*) placodes at late stage 11, showing both percentage of cells in different-size bins (large graph) as well as the cumulative percentage of cells relative to apical area size (inset: Kolmogorov-Smirnov two-sample test, $^{**}p << 0.01$; see Table S1). For GFP-Shot-EFGas2 expression, four placodes, and for the control, three placodes were segmented and analyzed, and the total number of cells traced was N(*nanosGal4*) = 339 and N(*UAS-GFP-Shot-EFGas2 nanosGal4 & fkhGal4*) = 348.
See also Figure S6.

the salivary gland placode is under tension during invagination (Röper, 2012). This cable likely collaborates with medial actomyosin to provide a stabilizing tissue-sized ratchet. How all these mechanisms are coordinated will be important to determine in the future.

The MT cytoskeleton is emerging as a crucial player in morphogenesis, both through previously characterized functions such as regulation of adherens junction stability and the positioning, delivery, and turnover of membrane components, and now through a direct role in stabilizing the dynamic apical medial actomyosin network. This network in turn is crucial for the effective change in apical surface area and shape and for tube formation. Further studies will reveal whether the interplay between MTs and actomyosin revealed here is conserved during other invagination processes in invertebrates and vertebrates.

EXPERIMENTAL PROCEDURES

Fly Stocks and Husbandry

For a full list of fly stocks and crosses, see Supplemental Experimental Procedures.

Embryo Immunofluorescence Labeling and Confocal and Live Analysis

Embryos were fixed, stained, and imaged using standard procedures; for details, please refer to Supplemental Experimental Procedures.

Automated Cell Segmentation, Tracking, and Myosin Quantification

For the analysis of apical cell area and neighbor analysis, images of fixed embryos of late stage 11 placodes (judged by the extent of tracheal development) were labeled with DE-cadherin to highlight cell membranes and with dCrebA or *fkhGal4*-driven GFP expression to mark salivary gland fate. For analyzing cell-contraction rates and myosin fluctuations, nine wild-type and three MT-depleted (Spastin) embryos labeled with *sqhGFP* and *Gap43-Cherry* were imaged live for between 7 and 28 min within a 50 min window at mid to late stage 11. Image stacks through apices of cells in the salivary placode were taken for both channels approximately every 20 s at 1-μm-depth intervals.

Cells were segmented in image stacks of fixed embryos, and cells were tracked and medial myosin fluorescence was quantified in movies with cell-tracking and analysis software as used previously (Blanchard et al., 2009, 2010; Butler et al., 2009; Gorfinkiel and Blanchard, 2011; Gorfinkiel et al., 2009). Briefly, the shape of the curved placode surface was identified in each z stack as a contiguous "blanket" spread over the cortical signal. Quasi-2D images for cell tracking containing clear cell cortices were extracted as a maximum-intensity projection of the 1- or 1.5-μm-thick layer of tissue below the blanket. These images were segmented using an adaptive watershedding algorithm, and in parallel cells were linked in time. Manual correction was used to perfect cell outlines for fixed embryos and to improve cell tracking in movies where the *GAP43-mCherry* fluorescence was sometimes faint. We imposed a coordinate system on each placode with the center of the pit at the origin, with anterior distance from the pit to the left and ventral distance down. Only cells of the salivary placode were used in subsequent analyses. These were distinguished from dCrebA staining in fixed embryos and from the cable surrounding the placode in the myosin channel of movies.

For the analyses of apical cell area in control versus MT-depleted fixed embryos, the clustering of cells of similar sizes was calculated for every cell within the placode as the average difference in apical area between a cell and all of its neighbors.

For the comparison of contraction rates and medial myosin behavior between control and MT-depleted embryos, tracked cells were first subjected to quality control. Cells were filtered using rules for inappropriate cell size, relative speed to neighbors, and rate of change in cell area. Incomplete cells at the edges of the field of view and those with short lineages were also removed from further analysis. Instantaneous tissue strain rates were calculated as previously for the remaining valid cells (Blanchard et al., 2009). Average medial myosin fluorescence intensity for each cell was calculated from the *sqhGFP* channel and myosin fluctuation analysis was performed as before (Blanchard et al., 2010), taking care to quantify myosin in the most apical layer(s) under the placode surface. Detrended cell-radius and myosin fluorescence intensity measures were calculated as the raw time series minus a smoothed time series trend (using a boxcar smoothing window of 6 min, larger than the maximum expected fluctuation cycle length).

Fluctuation analysis yielded cycle length, amplitude, and phase measures for each full cycle (defined for myosin as trough to trough and for cell radius as peak to peak). Periods of time for each cell that were not fluctuating were identified where amplitude multiplied by frequency was below a threshold that we established visually as being a good compromise between including real periodic behavior and excluding nonperiodic noise. The threshold for myosin fluctuation was 0.5, and 0.005 for cell-radius fluctuations. We then calculated the proportion of tracked cell time for which cells exhibited either myosin or cell-radius fluctuations.

Quantification of Colocalization and Fluorescence Intensity

Colocalization analysis and fluorescence intensity quantifications were performed using standard procedures. For details, please refer to Supplemental Experimental Procedures.

Statistical Analysis

Significance was determined using two-tailed Student's t test, Kolmogorov-Smirnov test, or G test of independence. Results were considered significant when $p < 0.05$. Specific tests, test statistics, degrees of freedom, and p values can be found in Table S1.

SUPPLEMENTAL INFORMATION

Supplemental Information includes Supplemental Experimental Procedures, six figures, one table, and seven movies and can be found with this article online at http://dx.doi.org/10.1016/j.devcel.2014.03.023.

ACKNOWLEDGMENTS

The authors would like to thank Debbie Andrew, Christian Dahmann, and the Developmental Studies Hybridoma Bank at the University of Iowa for providing reagents; Nick Brown for the use of his confocal microscope; and Sean Munro, Melina Schuh, Bénédicte Sanson, and members of the Röper laboratory for useful comments on the manuscript. This work was supported by a Biotechnology and Biological Sciences Research Council (BBSRC) doctoral studentship (to A.J.R.B.) and grants from the BBSRC (BB/B501798/1), Wellcome Trust (WT090279MA), and Medical Research Council (U105178780) (to K.R.). G.B.B. was supported by a BBSRC grant (BB/J010278/1) to R.J.A. and Bénédicte Sanson.

Received: October 29, 2013
Revised: February 28, 2014
Accepted: March 27, 2014
Published: June 9, 2014

REFERENCES

Akhmanova, A., Stehbens, S.J., and Yap, A.S. (2009). Touch, grasp, deliver and control: functional cross-talk between microtubules and cell adhesions. Traffic *10*, 268–274.

Andrew, D.J., and Ewald, A.J. (2010). Morphogenesis of epithelial tubes: Insights into tube formation, elongation, and elaboration. Dev. Biol. *341*, 34–55.

Bartolini, F., and Gundersen, G.G. (2006). Generation of noncentrosomal microtubule arrays. J. Cell Sci. *119*, 4155–4163.

Basu, R., and Chang, F. (2007). Shaping the actin cytoskeleton using microtubule tips. Curr. Opin. Cell Biol. *19*, 88–94.

CellPress

Bertet, C., Sulak, L., and Lecuit, T. (2004). Myosin-dependent junction remodelling controls planar cell intercalation and axis elongation. Nature *429*, 667–671.

Blake, K.J., Myette, G., and Jack, J. (1998). The products of ribbon and raw are necessary for proper cell shape and cellular localization of nonmuscle myosin in Drosophila. Dev. Biol. *203*, 177–188.

Blanchard, G.B., Kabla, A.J., Schultz, N.L., Butler, L.C., Sanson, B., Gorfinkiel, N., Mahadevan, L., and Adams, R.J. (2009). Tissue tectonics: morphogenetic strain rates, cell shape change and intercalation. Nat. Methods *6*, 458–464.

Blanchard, G.B., Murugesu, S., Adams, R.J., Martinez-Arias, A., and Gorfinkiel, N. (2010). Cytoskeletal dynamics and supracellular organisation of cell shape fluctuations during dorsal closure. Development *137*, 2743–2752.

Bolinger, C., Zasadil, L., Rizaldy, R., and Hildebrand, J.D. (2010). Specific isoforms of Drosophila shroom define spatial requirements for the induction of apical constriction. Dev. Dyn. *239*, 2078–2093.

Bosher, J.M., Hahn, B.S., Legouis, R., Sookhareea, S., Weimer, R.M., Gansmuller, A., Chisholm, A.D., Rose, A.M., Bessereau, J.L., and Labouesse, M. (2003). The Caenorhabditis elegans vab-10 spectraplakin isoforms protect the epidermis against internal and external forces. J. Cell Biol. *161*, 757–768.

Brodu, V., Baffet, A.D., Le Droguen, P.M., Casanova, J., and Guichet, A. (2010). A developmentally regulated two-step process generates a noncentrosomal microtubule network in Drosophila tracheal cells. Dev. Cell *18*, 790–801.

Broussard, J.A., Webb, D.J., and Kaverina, I. (2008). Asymmetric focal adhesion disassembly in motile cells. Curr. Opin. Cell Biol. *20*, 85–90.

Bush, K.T., Lynch, F.J., DeNittis, A.S., Steinberg, A.B., Lee, H.Y., and Nagele, R.G. (1990). Neural tube formation in the mouse: a morphometric and computerized three-dimensional reconstruction study of the relationship between apical constriction of neuroepithelial cells and the shape of the neuroepithelium. Anat. Embryol. (Berl.) *181*, 49–58.

Butler, L.C., Blanchard, G.B., Kabla, A.J., Lawrence, N.J., Welchman, D.P., Mahadevan, L., Adams, R.J., and Sanson, B. (2009). Cell shape changes indicate a role for extrinsic tensile forces in Drosophila germ-band extension. Nat. Cell Biol. *11*, 859–864.

Clark, I.E., Jan, L.Y., and Jan, Y.N. (1997). Reciprocal localization of Nod and kinesin fusion proteins indicates microtubule polarity in the Drosophila oocyte, epithelium, neuron and muscle. Development *124*, 461–470.

Corrigall, D., Walther, R.F., Rodriguez, L., Fichelson, P., and Pichaud, F. (2007). Hedgehog signaling is a principal inducer of Myosin-II-driven cell ingression in Drosophila epithelia. Dev. Cell *13*, 730–742.

Costantini, F., and Kopan, R. (2010). Patterning a complex organ: branching morphogenesis and nephron segmentation in kidney development. Dev. Cell *18*, 698–712.

David, D.J., Tishkina, A., and Harris, T.J. (2010). The PAR complex regulates pulsed actomyosin contractions during amnioserosa apical constriction in Drosophila. Development *137*, 1645–1655.

D'Avino, P.P., Takeda, T., Capalbo, L., Zhang, W., Lilley, K.S., Laue, E.D., and Glover, D.M. (2008). Interaction between Anillin and RacGAP50C connects the actomyosin contractile ring with spindle microtubules at the cell division site. J. Cell Sci. *121*, 1151–1158.

Feldman, J.L., and Priess, J.R. (2012). A role for the centrosome and PAR-3 in the hand-off of MTOC function during epithelial polarization. Curr. Biol. *22*, 575–582.

Fernandez-Gonzalez, R., and Zallen, J.A. (2011). Oscillatory behaviors and hierarchical assembly of contractile structures in intercalating cells. Phys. Biol. *8*, 045005.

Gorfinkiel, N., and Blanchard, G.B. (2011). Dynamics of actomyosin contractile activity during epithelial morphogenesis. Curr. Opin. Cell Biol. *23*, 531–539.

Gorfinkiel, N., Blanchard, G.B., Adams, R.J., and Martinez Arias, A. (2009). Mechanical control of global cell behaviour during dorsal closure in Drosophila. Development *136*, 1889–1898.

Hetherington, S., Gally, C., Fritz, J.A., Polanowska, J., Reboul, J., Schwab, Y., Zahreddine, H., Behm, C., and Labouesse, M. (2011). PAT-12, a potential antinematode target, is a new spectraplakin partner essential for Caenorhabditis elegans hemidesmosome integrity and embryonic morphogenesis. Dev. Biol. *350*, 267–278.

Jankovics, F., and Brunner, D. (2006). Transiently reorganized microtubules are essential for zippering during dorsal closure in Drosophila melanogaster. Dev. Cell *11*, 375–385.

Lee, S., and Kolodziej, P.A. (2002). The plakin Short Stop and the RhoA GTPase are required for E-cadherin-dependent apical surface remodeling during tracheal tube fusion. Development *129*, 1509–1520.

Lee, J.Y., and Harland, R.M. (2007). Actomyosin contractility and microtubules drive apical constriction in Xenopus bottle cells. Dev. Biol. *311*, 40–52.

Lee, C., Scherr, H.M., and Wallingford, J.B. (2007). Shroom family proteins regulate gamma-tubulin distribution and microtubule architecture during epithelial cell shape change. Development *134*, 1431–1441.

Martin, A.C., Kaschube, M., and Wieschaus, E.F. (2009). Pulsed contractions of an actin-myosin network drive apical constriction. Nature *457*, 495–499.

Martin, A.C., Gelbart, M., Fernandez-Gonzalez, R., Kaschube, M., and Wieschaus, E.F. (2010). Integration of contractile forces during tissue invagination. J. Cell Biol. *188*, 735–749.

Mason, F.M., Tworoger, M., and Martin, A.C. (2013). Apical domain polarization localizes actin-myosin activity to drive ratchet-like apical constriction. Nat. Cell Biol. *15*, 926–936.

Maybeck, V., and Röper, K. (2009). A targeted gain-of-function screen identifies genes affecting salivary gland morphogenesis/tubulogenesis in Drosophila. Genetics *181*, 543–565.

Mimori-Kiyosue, Y. (2011). Shaping microtubules into diverse patterns: molecular connections for setting up both ends. Cytoskeleton (Hoboken) *68*, 603–618.

Mogensen, M.M. (1999). Microtubule release and capture in epithelial cells. Biol. Cell *91*, 331–341.

Mohan, S., Rizaldy, R., Das, D., Bauer, R.J., Heroux, A., Trakselis, M.A., Hildebrand, J.D., and VanDemark, A.P. (2012). Structure of Shroom domain 2 reveals a three-segmented coiled-coil required for dimerization, Rock binding, and apical constriction. Mol. Biol. Cell *23*, 2131–2142.

Myat, M.M., and Andrew, D.J. (2000). Organ shape in the Drosophila salivary gland is controlled by regulated, sequential internalization of the primordia. Development *127*, 679–691.

Myat, M.M., and Andrew, D.J. (2002). Epithelial tube morphology is determined by the polarized growth and delivery of apical membrane. Cell *111*, 879–891.

Pollard, T.D., and Cooper, J.A. (2009). Actin, a central player in cell shape and movement. Science *326*, 1208–1212.

Pope, K.L., and Harris, T.J. (2008). Control of cell flattening and junctional remodeling during squamous epithelial morphogenesis in Drosophila. Development *135*, 2227–2238.

Rauzi, M., Lenne, P.F., and Lecuit, T. (2010). Planar polarized actomyosin contractile flows control epithelial junction remodelling. Nature *468*, 1110–1114.

Roll-Mecak, A., and Vale, R.D. (2008). Structural basis of microtubule severing by the hereditary spastic paraplegia protein spastin. Nature *451*, 363–367.

Röper, K. (2012). Anisotropy of Crumbs and aPKC drives myosin cable assembly during tube formation. Dev. Cell *23*, 939–953.

Röper, K., and Brown, N.H. (2003). Maintaining epithelial integrity: a function for gigantic spectraplakin isoforms in adherens junctions. J. Cell Biol. *162*, 1305–1315.

Röper, K., and Brown, N.H. (2004). A spectraplakin is enriched on the fusome and organizes microtubules during oocyte specification in Drosophila. Curr. Biol. *14*, 99–110.

Röper, K., Gregory, S.L., and Brown, N.H. (2002). The 'spectraplakins': cytoskeletal giants with characteristics of both spectrin and plakin families. J. Cell Sci. *115*, 4215–4225.

Royou, A., Field, C., Sisson, J.C., Sullivan, W., and Karess, R. (2004). Reassessing the role and dynamics of nonmuscle myosin II during furrow formation in early Drosophila embryos. Mol. Biol. Cell *15*, 838–850.

Sawyer, J.K., Choi, W., Jung, K.C., He, L., Harris, N.J., and Peifer, M. (2011). A contractile actomyosin network linked to adherens junctions by Canoe/afadin helps drive convergent extension. Mol. Biol. Cell *22*, 2491–2508.

Schaefer, A.W., Schoonderwoert, V.T., Ji, L., Mederios, N., Danuser, G., and Forscher, P. (2008). Coordination of actin filament and microtubule dynamics during neurite outgrowth. Dev. Cell *15*, 146–162.

Simões, Sde.M., Blankenship, J.T., Weitz, O., Farrell, D.L., Tamada, M., Fernandez-Gonzalez, R., and Zallen, J.A. (2010). Rho-kinase directs Bazooka/Par-3 planar polarity during Drosophila axis elongation. Dev. Cell *19*, 377–388.

Simões, Sde.M., Mainieri, A., and Zallen, J.A. (2014). Rho GTPase and Shroom direct planar polarized actomyosin contractility during convergent extension. J. Cell Biol. *204*, 575–589.

Solon, J., Kaya-Copur, A., Colombelli, J., and Brunner, D. (2009). Pulsed forces timed by a ratchet-like mechanism drive directed tissue movement during dorsal closure. Cell *137*, 1331–1342.

Somers, W.G., and Saint, R. (2003). A RhoGEF and Rho family GTPase-activating protein complex links the contractile ring to cortical microtubules at the onset of cytokinesis. Dev. Cell *4*, 29–39.

Stramer, B., Moreira, S., Millard, T., Evans, I., Huang, C.Y., Sabet, O., Milner, M., Dunn, G., Martin, P., and Wood, W. (2010). Clasp-mediated microtubule bundling regulates persistent motility and contact repulsion in Drosophila macrophages in vivo. J. Cell Biol. *189*, 681–689.

Suozzi, K.C., Wu, X., and Fuchs, E. (2012). Spectraplakins: master orchestrators of cytoskeletal dynamics. J. Cell Biol. *197*, 465–475.

Vale, R.D., Spudich, J.A., and Griffis, E.R. (2009). Dynamics of myosin, microtubules, and Kinesin-6 at the cortex during cytokinesis in Drosophila S2 cells. J. Cell Biol. *186*, 727–738.

Warburton, D., El-Hashash, A., Carraro, G., Tiozzo, C., Sala, F., Rogers, O., De Langhe, S., Kemp, P.J., Riccardi, D., Torday, J., et al. (2010). Lung organogenesis. Curr. Top. Dev. Biol. *90*, 73–158.

Westermann, S., and Weber, K. (2003). Post-translational modifications regulate microtubule function. Nat. Rev. Mol. Cell Biol. *4*, 938–947.

Zhang, D., Grode, K.D., Stewman, S.F., Diaz-Valencia, J.D., Liebling, E., Rath, U., Riera, T., Currie, J.D., Buster, D.W., Asenjo, A.B., et al. (2011). Drosophila katanin is a microtubule depolymerase that regulates cortical-microtubule plus-end interactions and cell migration. Nat. Cell Biol. *13*, 361–370.

Developmental Cell, Volume *29*

Supplemental Information

A Dynamic Microtubule Cytoskeleton Directs Medial Actomyosin Function during Tube Formation

Alexander J.R. Booth, Guy B. Blanchard, Richard J. Adams, and Katja Röper

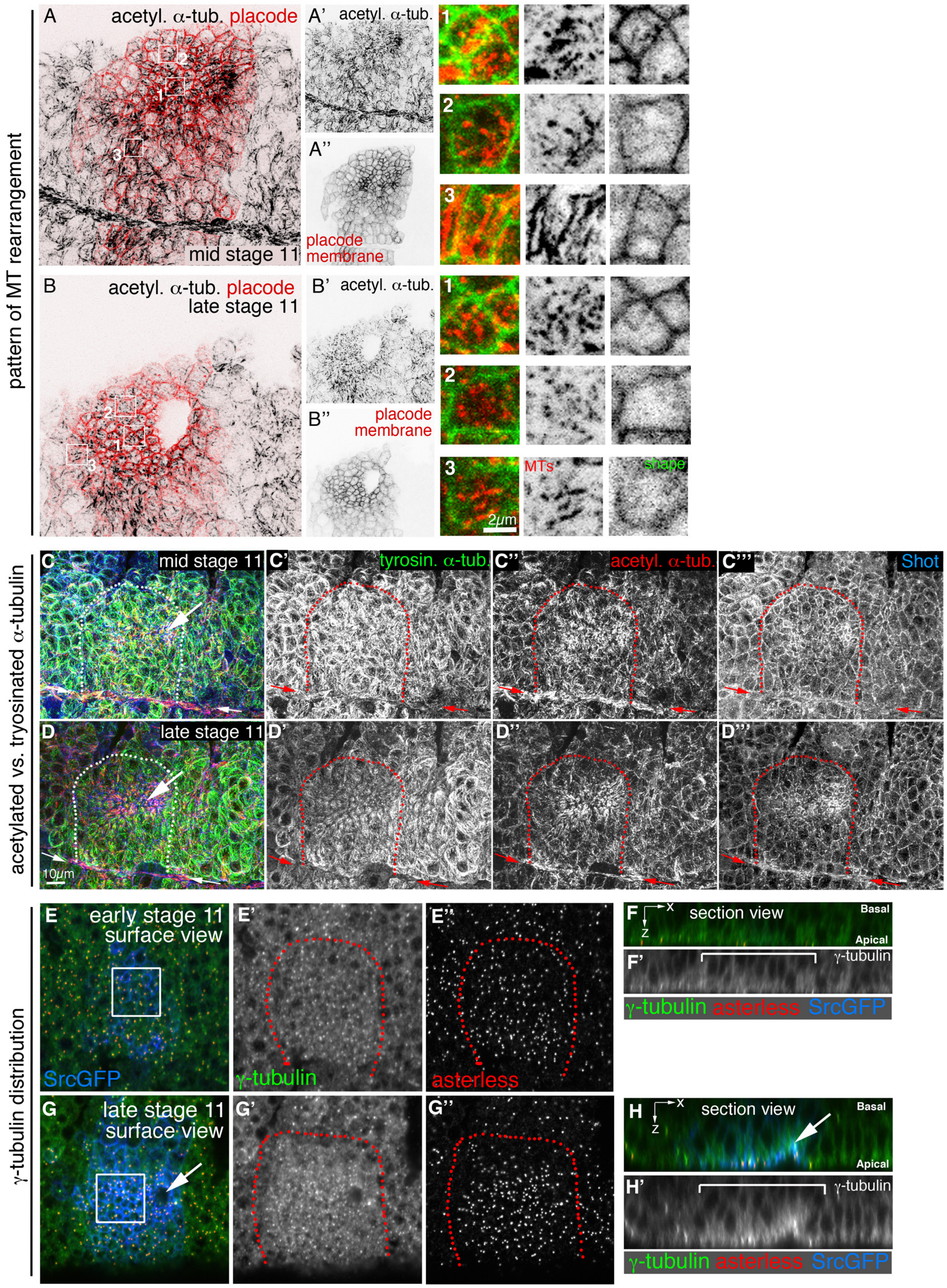
pattern of MT rearrangement
A
acetyl. α-tub. placode
mid stage 11
A' acetyl. α-tub.
A''
placode membrane
B
acetyl. α-tub. placode
late stage 11
B' acetyl. α-tub.
B''
placode membrane
MTs
shape
2μm
acetylated vs. tryosinated α-tubulin
C mid stage 11
C' tyrosin. α-tub.
C'' acetyl. α-tub.
C''' Shot
D late stage 11
D'
D''
D'''
10μm
γ-tubulin distribution
E early stage 11 surface view
SrcGFP
E'
γ-tubulin
E''
asterless
F section view
Basal
Apical
F'
γ-tubulin
γ-tubulin asterless SrcGFP
G late stage 11 surface view
G'
G''
H section view
Basal
Apical
H'
γ-tubulin
γ-tubulin asterless SrcGFP

Supplemental Figure S1 (related to Fig.1 and 2). Microtubule analysis during early gland invagination.

A-B'' The pattern of MT rearrangement across the placode during stage 11 mirrors the pattern of constriction, starting with the forming pit in the dorsal-posterior comer and moving anterior and ventral. MTs (marked by acetylated α-tubulin; black in **A**,**B** single in **A'**,**B'**, red in small panels) are shown in comparison to cell outlines (membranes of placodal cells marked by srcGFP; red in **A**,**B**, single in **A''**,**B''**, green in small panels). Small panels show examples of cells across the placode. Note that constricted and unconstricted cells with rearranged longitudinal MTs can be found (1 vs 2), but parallel apical bundles are usually only seen in unconstricted cells (3).

C-D''' Comparison of acetylated α-tubulin (red in **C**,**D**, single in **C''**, **D''**) and tyrosinated α-tubulin (green in **C**,**D**, single in **C''**, **D''**) in mid and late stage 11 placodes. Note that tyrosinated α-tubulin levels appear fairly even across epidermis and placode, whereas acetylated α-tubulin levels appear increased in constricting placodal cells. Placodes marked by dotted lines, small arrows point to ventral midline, larger arrows point to the forming pit. Shot (blue) marks cell outlines.

E-H' γ-tubulin distribution during early gland invagination Comparison of the centrosomal marker asterless (red) to γ-tubulin (green) labeling at early stage 11 (**E, F**) and late stage 11 (**G,H**). **E** and **G** show stacks of confocal images grazing the surface of the placode (surface views), whereas **F** and **H** are section views in z. The white boxes in **E, G** indicate the area shown in Fig. 2G,H; the red dotted lines in surface views and the white brackets in section views indicate the area of the placode (identified by *srcGFP* labeling using *fkhGal4*; blue). Arrows point to the invaginating pit at late stage 11.

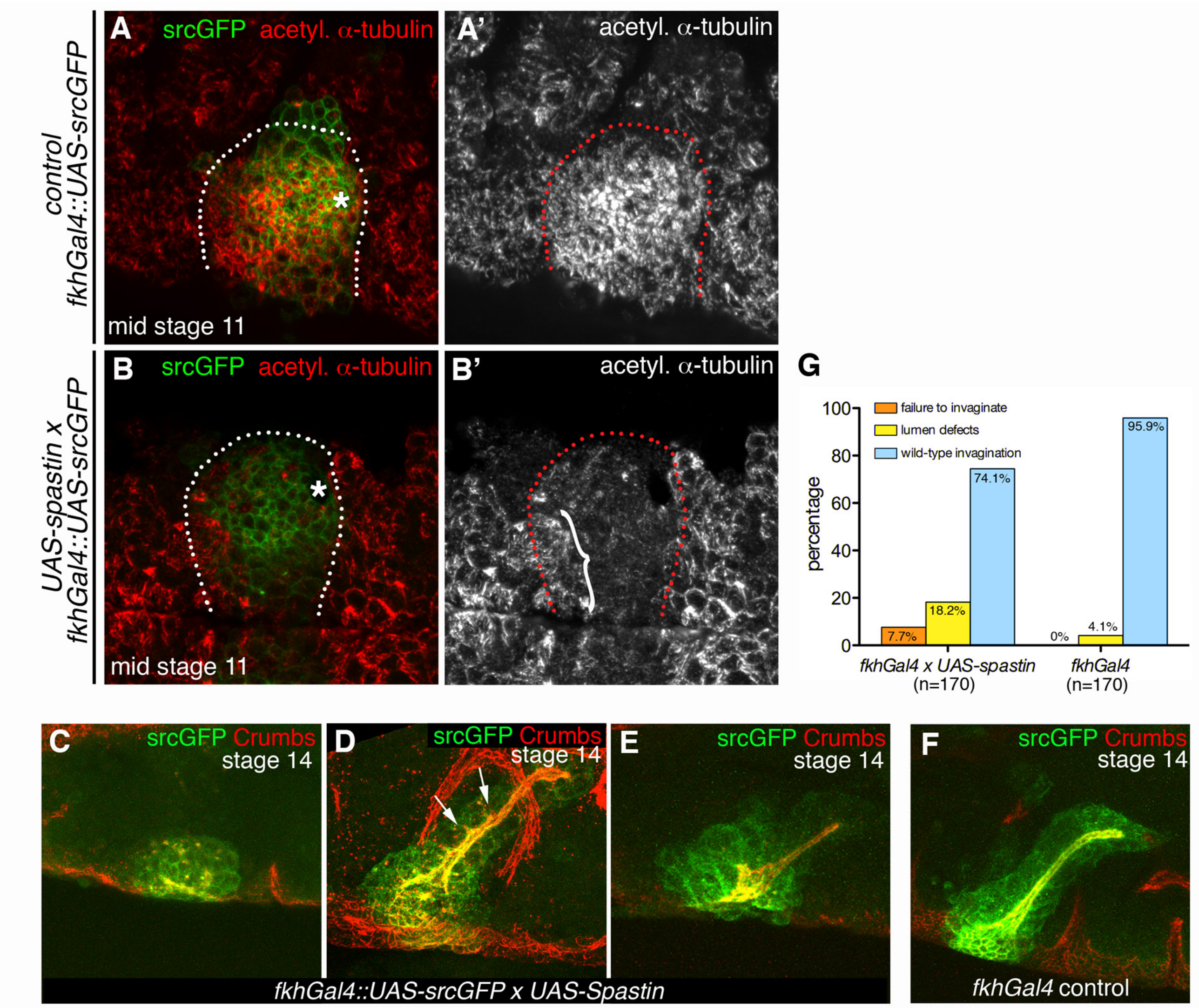

Supplemental Figure S2 (related to Fig.3). Depletion of microtubules in the salivary gland placode and resulting late embryonic phenotypes.

Expression of *UAS-Spastin* under *fkhGal4* control was used to deplete MTs specifically from the salivary gland placode.

Control placodes (**A**; placode area marked by *srcGFP*, green) show dense MTs (red) within the placode, whereas expression of UAS-Spastin under control of *fkhGal4* within the placode (**B**) leads to depletion of MTs (red) in 36% of placodes at stage 11. The dotted lines mark the boundary of the placode, the asterisks mark the invagination point. Note the loss of strong acetylated α-tubulin staining in the MT-

depleted placode. White bracket indicates placodal area where MT-depletion is incomplete, due to reduced levels of *fkhGal4* expression in this area.

C-F Analysis of MT-depleted placodes at stage 14. In contrast to wild-type placodes that have nearly completely invaginated at stage 14 (**F**), MT-depleted placodes show a range of phenotypes from complete failure to invaginate (**C**) to ectopic branching (**D**; arrows) and irregular invagination (**E**). Apical membrane is marked by Crumbs (red), and placode marked by *srcGFP* (green).

G Quantification of phenotypes at stage 14 of embryos expressing *UAS-Spastin* driven by *fkhGal4* or control embryos with only *fkhGal4* (n=170 glands for each genotype). Note that similar to stage 11, only a fraction of glands show depleted MTs when Spastin is expressed at this stage, and these MT-depleted glands show the phenotypes illustrated in **C-E**.

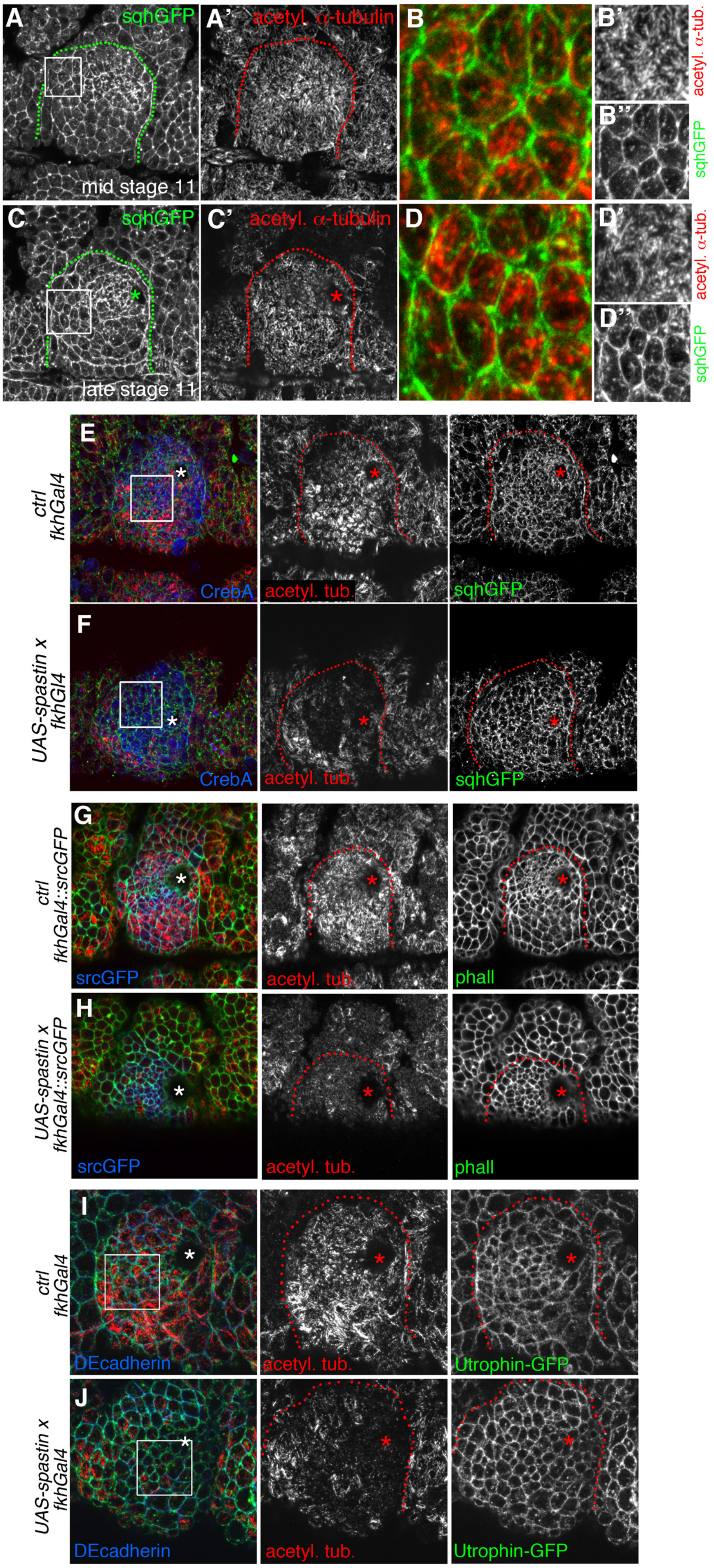
A
sqhGFP
mid stage 11
A'
acetyl. α-tubulin
B
B'
acetyl. α-tub.
B''
sqhGFP
C
sqhGFP
late stage 11
C'
acetyl. α-tubulin
D
D'
acetyl. α-tub.
D''
sqhGFP
E
ctrl
fkhGal4
CrebA
acetyl. tub.
sqhGFP
F
UAS-spastin x
fkhGl4
CrebA
acetyl. tub.
sqhGFP
G
ctrl
fkhGal4::srcGFP
srcGFP
acetyl. tub.
phall
H
UAS-spastin x
fkhGal4::srcGFP
srcGFP
acetyl. tub.
phall
I
ctrl
fkhGal4
DEcadherin
acetyl. tub.
Utrophin-GFP
J
UAS-spastin x
fkhGal4
DEcadherin
acetyl. tub.
Utrophin-GFP

Supplemental Figure S3 (related to Fig.4). Analysis of actomyosin in control and microtubule-depleted placodes.

A-D'' Apical medial myosin localisation in non-constricted cells at mid (**A**,**B**) and late (**C**,**D**) stage 11 in the anterior region of the placode. *sqhGFP* (**A**,**C**, green in **B**, **D** and **B''**,**D''**), acetylated α-tubulin (**A'**,**C'**, red in **B**, **D** and **B'**,**D'**). White boxes in **A**, **C** indicate the magnified areas in **B** and **D**. Note that in these unconstricted cells with mostly apical parallel MT bundles, MTs and medial myosin do not colocalise.

E-J Effect of microtubule-depletion on actin and myosin in the placode.

E,F Analysis of *sqhGFP* (green) in control placodes (**E**; *fkhGal4*) and MT-depleted placodes (**F**; *UAS-Spastin x fkhGal4*); placode area labeled by CrebA (blue); acetylated α-tubulin (red). The white boxes indicate the areas shown in higher magnification in Fig. 4E,G.

G-J Analysis of actin (green; using phalloidin [**G,H**] or *utrophinGFP* [**I,J**) in control placodes (**G,I**; *fkhGal4*) and MT-depleted placodes (**H,J**; *UAS-Spastin x fkhGal4*); placode area labeled by *srcGFP* (blue); acetylated α-tubulin (red); DE-Cadherin (blue). The white boxes in **I, J** indicate the areas shown in higher magnification in Fig. 4F,H.

Asterisks mark the invagination point, dotted lines mark the circumference of the placode.

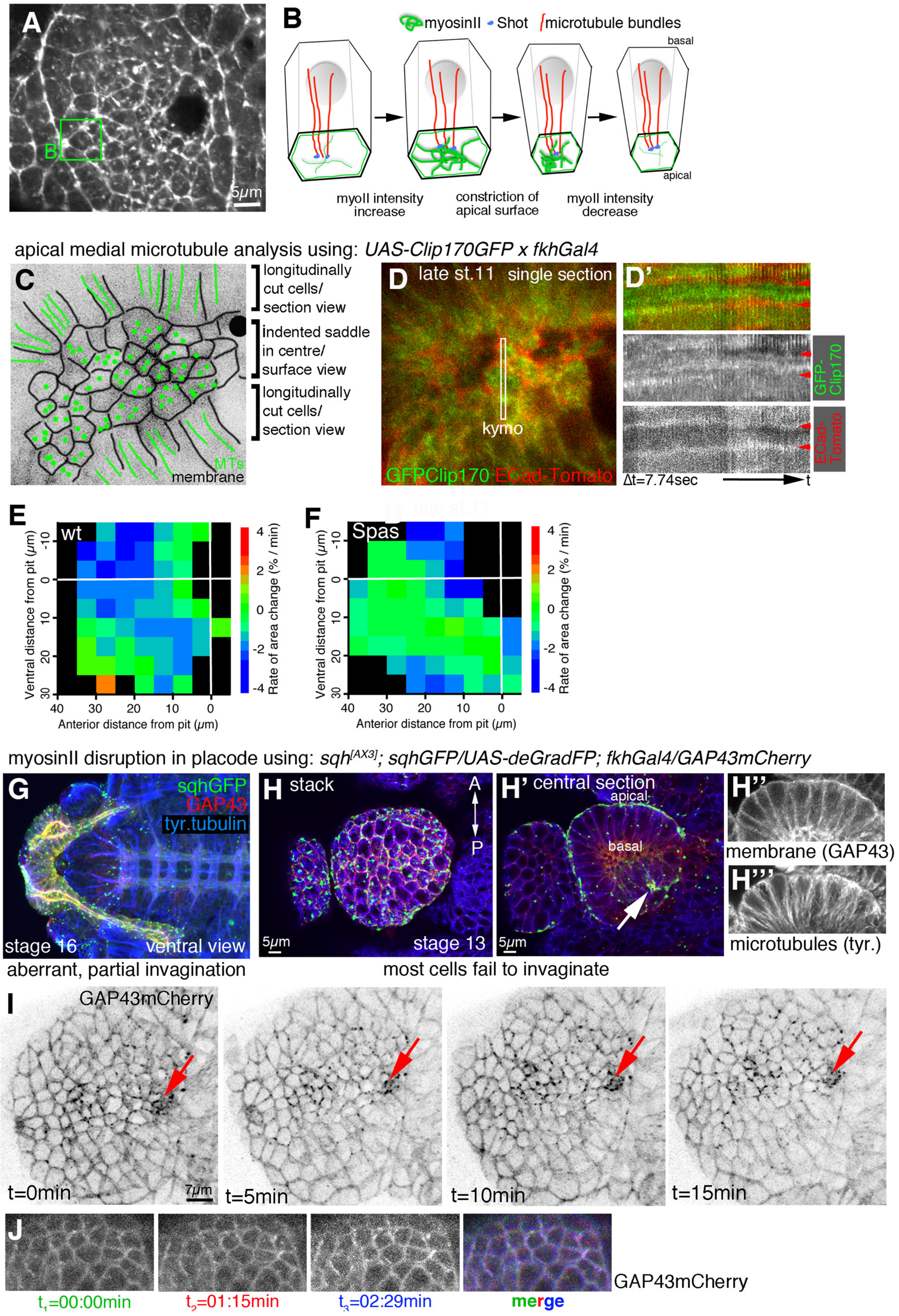

A
B
5μm
B
myosinII
Shot
microtubule bundles
basal
apical
myoII intensity increase
constriction of apical surface
myoII intensity decrease
apical medial microtubule analysis using: UAS-Clip170GFP x fkhGal4
C
longitudinally cut cells/ section view
indented saddle in centre/ surface view
longitudinally cut cells/ section view
MTs
membrane
D
late st.11
single section
kymo
GFPClip170
ECad-Tomato
D'
GFP-Clip170
ECad-Tomato
Δt=7.74sec
t
E
wt
Ventral distance from pit (μm)
Anterior distance from pit (μm)
Rate of area change (% / min)
F
Spas
Ventral distance from pit (μm)
Anterior distance from pit (μm)
Rate of area change (% / min)
myosinII disruption in placode using: sqh[AX3]; sqhGFP/UAS-deGradFP; fkhGal4/GAP43mCherry
G
sqhGFP
GAP43
tyr.tubulin
stage 16
ventral view
aberrant, partial invagination
H
stack
A
P
5μm
stage 13
H'
central section
apical
basal
5μm
H''
membrane (GAP43)
H'''
microtubules (tyr.)
most cells fail to invaginate
I
GAP43mCherry
t=0min
7μm
t=5min
t=10min
t=15min
J
GAP43mCherry
t1=00:00min
t2=01:15min
t3=02:29min
merge

Supplemental Figure S4 (related to Fig.5). Placodal cells show a contractile medial actomyosin network crucial for apical constriction and gland invagination.

A Overview of a placode of a *sqh*^{AX3}*; sqh::sqhGFP42* embryo used for time-lapse analysis. The green box indicates the area shown as stills in Fig.5B.

B Schematic model of apical medial actomyosin function in placodal cells: cycles of increase of medial myosin lead to apical surface constriction, probably through constriction of the whole network and through pulling of actomyosin fibres on associated junctional complexes. The medial actomyosin network is stabilised by longitudinal MT bundles, with the cytolinker Shot providing the link between MTs and actin.

C-D' Live analysis of medial MT end dynamics in placodal cells. MTs were labeled using *GFP-Clip170*. **C** is a schematic of the area shown in the still image in **D** and in Supplemental Movie 3 (MTs in green and cell boundaries in black), with only the cells marked in the middle being visible in a surface view, and the more peripheral cells being viewed in section views, due to the saddle-like shape of the tissue at this stage. **D** Still image of Suppl.Movie 3; the white box is indicating the area that is shown in the kymograph in **D'**. Frames are 7.74 sec apart, the last frame is at 379.26 sec, cell junctions are marked by E-Cadherin-Tomato (red); arrowheads in **D'** indicate the positions of two cell boundaries.

E,**F** Average rates of change of tissue area across the salivary gland placode. Anterior is left, ventral is down, with pit located at [0,0] where white lines cross. Data pooled from cell tracks sampled approx. every 20 seconds covering 50 minutes of mid to late stage 11. Grid squares with fewer than N = 20 cell instances were discarded. **E** Data pooled from 9 control embryos (total N cell instances = 4895). **F** Data pooled from three MT-depleted embryos (total N cell instances = 5126).

The same data are also depicted in different form in Fig. 5K.

G-H''' Targeting placodal myosin II for degradation impairs apical constriction and gland invagination. In flies of the genotype *sqh*^{AX3}*; sqh::sqhGFP42/UAS-deGradFP; fkhGal4/GAP43mCherry sqhGFP* is the only source of myosin II and is targeted for degradation by the proteasome specifically in the salivary glands. These embryos show glands that are only partially invaginated at stage 16 (**G**) or glands where most cells failed to constrict apically and invaginate, leaving two bulges on the outside of the embryo (**H**; in this panel anterior is up; arrow in **H'** points to the lumen of the only invaginated portion, the early pit). *sqhGFP* (green); *GAP43mCherry* (red); tyrosinated α-tubulin (blue). Note that *sqhGFP*, when targeted by *deGradFP*, accumulates in bright foci throughout the cells that appear to be non-functional as noted previously (Caussinus et al., 2012). Longitudinal MTs (**H'''**; compare to membrane marker in **H''**), similar to controls, can be seen in unconstricted cells in these embryos.

I,J Time-lapse analysis of glands in embryos of the genotype *sqh*^{AX3}*; sqh::sqhGFP42/UASdeGradFP; fkhGal4/GAP43mCherry* shows that (apart from the very early pit) placodal cells do not significantly constrict their apices. Panels in **I** show four time-points over 15 min of Suppl.Movie 6 (GAP43mCherry). **J** shows three individual time-points and a color-coded overlay of Suppl.Movie 7.

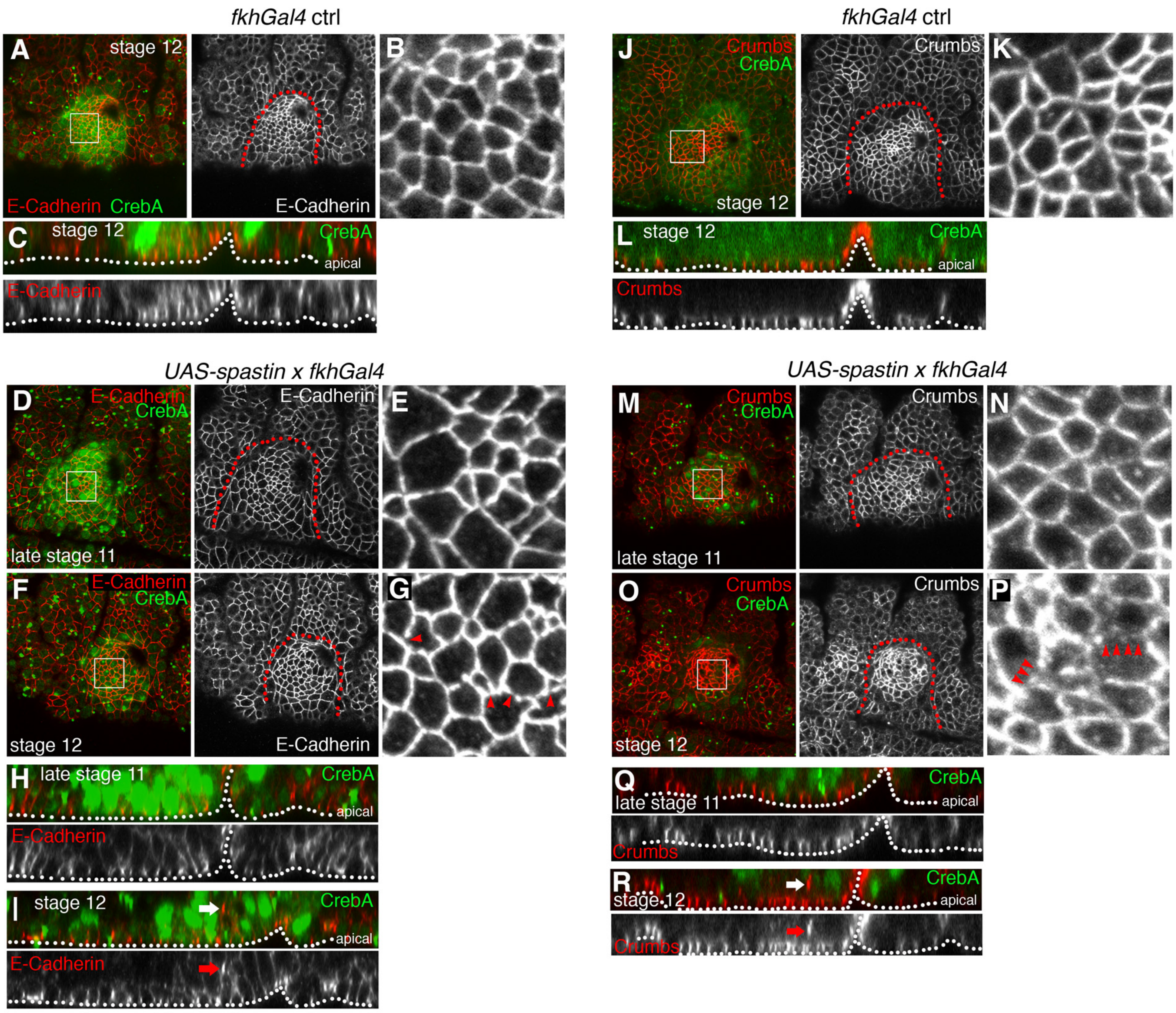

Supplemental Figure S5 (related to Fig.5). Effect of microtubule-depletion on E-Cadherin and Crumbs localisation in the placode.

A-C Control placodes (*fkhGal4*) labeled for E-Cadherin at stage 12. Shown are surface views (**A,B**) and section views (**C**) for CrebA (green), E-Cadherin (red). **B** shows a higher magnification of E-Cadherin of the area indicted by the box in **A**.

D-I MT-depleted placodes (*UAS-Spastin x fkhGal4*) at late stage 11 (**D**,**E** and **H**) and at stage 12 (**F**,**G** and **I**). Shown are surface views (**D-G**) and section views (**H,I**) for CrebA (green), E-Cadherin (red). Whereas E-Cadherin staining is indistinguishable from control placodes at late stage 11, at stage 12 discontinuities (arrowheads in **G**)

and internal foci (arrows in **I**) can be observed at times. Dotted lines mark the boundary of the placode (deduced from CrebA staining); dotted lines in section views mark the apical surface.

J-L Control placodes (*fkhGal4*) labeled for Crumbs at stage 12. Shown are surface views (**J,K**) and section views (**L**) for CrebA (green), Crumbs (red). **K** shows a higher magnification of E-Cadherin of the area indicted by the box in **J**.

M-R MT-depleted placodes (*UAS-Spastin x fkhGal4*) at late stage 11 (**M**,**N** and **Q**) and at stage 12 (**O**, **P** and **R**). Shown are surface views (**M-P**) and section views (**Q,R**) for CrebA (green), Crumbs (red). Whereas Crumbs staining is indistinguishable from control placodes at late stage 11, at stage 12 discontinuities (arrowheads) and internal foci (arrows) can be observed at times. Dotted lines mark the boundary of the placode (deduced from CrebA staining); dotted lines in section views mark the apical surface.

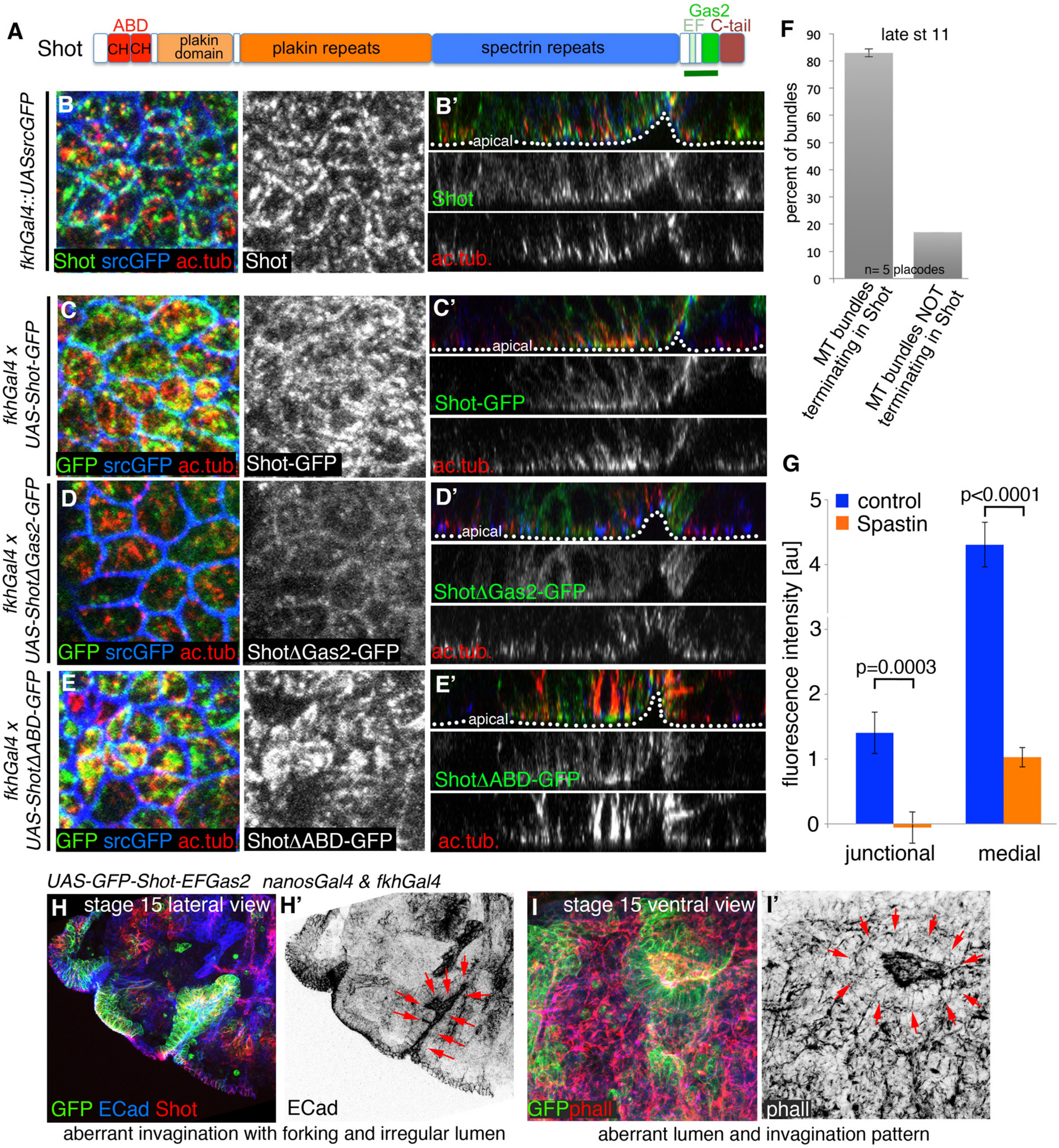

Supplemental Figure S6 (related to Figs 6 and 7). Analysis of Shot function in the placode.

A Schematic of Shot protein domain composition, with an actin-binding domain (ABD) composed of two calponin-homology (CH) domains at the N-terminus, and a microtubule-binding C-terminus including EF-hands and a Gas2-domain. The green

bar below the protein schematic indicates the region contained in Shot-EFGas2-GFP.

B-E' Shot requires its Gas2 domain to localise to the apical ends of microtubule bundles in the placode. At late stage 11, antibody labeling for Shot (green, **B,B'**) is indistinguishable from localisation of a GFP-tagged full length Shot protein (*UAS-Shot-GFP* under *fkhGal4* control; green, **C,C'**), both strongly label the apical (-) ends of MTs (red) within the placode. In contrast, a GFP-tagged version of Shot lacking the Gas2 domain (green, **D,D'**) fails to localise to MT apical (-) ends, whereas the GFP-tagged version of Shot lacking the ABD but containing the Gas2 domain (green, **E,E'**) strongly localised along the whole length of MTs, bundled them and stabilised them as evident from increased acetylated α-tubulin staining. Shown are surface (**B-E**) and section views (**B'-E'**). The apical surface is indicated by white dotted lines in the section views. Placodal membranes are marked with *srcGFP* (blue).

F 83% of MT bundles terminate in an apical focus of Shot. Shown are mean +/- SEM of bundles analysed in 5 placodes.

G Quantification of the effect of MT-depletion (using *UAS-Spastin* and *fkhGal4*) on Shot localisation within the placode at late stage 11 (shown are mean +/- SEM of placodal fluorescence intensity above epidermal base level; junctional: $p=0.0003$, medial $p<0.0001$ using Student's t-test, see Table 1). Upon MT-depletion, junctional Shot in the placode is reduced to levels of junctional Shot outside the placode, whereas medial Shot is strongly reduced.

H-I' Late gland phenotypes observed at stage 15 when endogenous Shot function was disrupted by expression of *UAS-GFP-Shot-EFGas2* under *nanosGal4* and *fkhGal4* control. Glands can show aberrant invagination with forking and irregular lumina (**H,H', I, I'**). *UAS-GFPShot-EFGas2* (green); endogenous Shot (red in **H**); E-Cadherin (blue in **H** and inverse in **H'**); phalloidin (red in **I** and inverse in **I'**). Red arrows point to the irregular lumen and gland shapes in both examples.

Figure and Panel:	Test Statistic	Sample Size / Degrees of Freedom	P-Value
Fig. 2D, centrosomal-acentrosomal MT ends	Unpaired Student's t-test t=8.854	df=10, n(early st11) = 326 MT bundles, 6 placodes n(late st11) = 384 MT bundles, 6 placodes	P<0.0001
Fig. 2J, acentrosomal γ-tubulin	Unpaired Student's t-test t=3.4	df=10, n(stage 10) = 6 placodes n(late stage 11) = 6 placodes	P=0.0069
Fig. 3E, cell area percentage of cells	K-S test. Dmax=0.1303 Dcrit for P<0.001=0.0810	n(control) = 1198 cells n(Spas) = 1122 cells, 10 placodes	P<<0.001
Fig. 3H, neighbour area difference percentage	K-S test. Dmax=0.0965 Dcrit for P<0.001=0.0810	n(control) = 1148 cells n(Spas) = 1117 cells, 10 placodes	P<<0.001
Fig. 4I, junctional myosin, ctrl vs MT-depleted	Unpaired Student's t-test t=1.912	df=58 n(control) = 30 cells, 3 placodes n(Spas) = 30 cells, 3 placodes	P=0.0609
Fig. 4I, medial myosin, ctrl vs MT-depleted	Unpaired Student's t-test t=6.556	df=58 n(control) = 30 cells, 3 placodes n(Spas) = 30 cells, 3 placodes	P<0.0001
Fig. 4J, junctional utrophin/actin, ctrl vs MT-depleted	Unpaired Student's t-test t=0.4676	df=58 n(control) = 30 cells, 3 placodes n(Spas) = 30 cells, 3 placodes	P=0.6418
Fig. 4J, medial utrophin/actin, ctrl vs MT-depleted	Unpaired Student's t-test t=4.214	df=58 n(control) = 30 cells, 3 placodes n(Spas) = 30 cells, 3 placodes	P<0.0001
Fig. 5G, Is percentage of cell time with myosin cycles the same for wt as for Spas?	G-Test of independence G=7747	n(control) = 2887 cells, 9 placodes n(Spas) = 3711 cells, 3 placodes	P<<0.001
Fig. 5H, length of myosin cycles	K-S test. Dmax=0.233 Dcrit for P<0.001=0.137	n(control) = 207 cells, 9 placodes n(Spas) = 154 cells, 3 placodes	P<<0.001
Fig. 5I, Is percentage of myosin cycling time that also has cell radius cycles the same for wt as for Spas?	G-Test of independence G=6410	n(control) = 2778 cells, 9 placodes n(Spas) = 2690 cells, 3 placodes	P<<0.001
Suppl.Fig. S6G, junctional Shot, ctrl vs MT-depleted	Unpaired Student's t-test t=3.729	df=88 n(control) = 40 cells, 4 placodes, n(Spas) = 50 cells, 5 placodes	P=0.0003
Suppl.Fig. S6G, medial Shot, ctrl vs MT-depleted	Unpaired Student's t-test t=9.333	df=88 n(control) = 40 cells, 4 placodes, n(Spas) = 50 cells, 5 placodes	P<0.0001
Fig. 7M, junctional Shot, ctrl vs GFP-Shot-EFGas2 expressing	Unpaired Student's t-test t=1.061	df=58 n(control) = 20 cells, 2 placodes n(Spas) = 40 cells, 4 placodes	P=0.2932

Fig. 7M, medial Shot, ctrl vs GFP-Shot-EFGas2 expressing	Unpaired Student's t-test t=2.613	df=58 n(control) = 20 cells, 2 placodes n(Spas) = 40 cells, 4 placodes	P=0.0114
Fig. 7N, junctional phall/actin, ctrl vs GFP-Shot-EFGas2 expressing	Unpaired Student's t-test t=1.248	df=198 n(control) = 100 cells, 6 placodes n(Spas) = 100 cells, 6 placodes	P=0.2134
Fig. 7M, medial phall/actin, ctrl vs GFP-Shot-EFGas2 expressing	Unpaired Student's t-test t=4.553	df=198 n(control) = 100 cells, 6 placodes n(Spas) =100 cells, 6 placodes	P<0.0001
Fig. 7Q, cell area percentage of cells	K-S test. Dmax=0.1315 Dcrit for P<0.01=0.1244	n(control) = 339 cells, 3 placodes n(Spas) = 348 cells, 4 placodes	P<<0.01

Supplemental Table S1 (related to Figs 2, 3, 4, 5, 7 and S6). Statistical analyses.

Significance was determined using two-tailed Student's *t*-test, Kolmogorov-Smirnov (K-S) test or G-test of independence. Results were considered significant when P<0.05. Specific tests, test statistics, degrees of freedom and p-values are all indicated above.

Supplemental Experimental Procedures

Fly stocks and husbandry

The following transgenes were used: *sqh^{AX3}; sqh::sqhGFP42*, and *yw; sqh::sqhGFP42; sqh::sqhGFP40* (Royou et al., 2004): *sqh^{AX3}; sqh::sqhGFP42; GAP43-mCherry*, *sqh::sqhmCherry* (Martin et al., 2009), *fkhGal4* (Henderson and Andrew, 2000; Zhou et al., 2001)[kind gift of Debbie Andrew]; *fkhGal4::srcGFP* (Maybeck and Röper, 2009), *UAS-Spastin* on X (Sherwood et al., 2004); *UAS-Katanin* (Christian Dahmann lab); *UAS-deGradFP II* (Caussinus et al., 2012); *UAS-GFP-Clip170* (Stramer et al., 2010); *sqh::UtrophinGFP* (Rauzi et al., 2010); *UASp-GFP-Shot-EFGas2* (Maybeck and Röper, 2009).

The following fly stocks were obtained from Bloomington and are described in FlyBase: *enGal4; nanosGal4VP16*.

To deplete MTs, virgins of *fkhGal4* were crossed to males of *UAS-Spastin (X)*. Using anti-tubulin immunofluorescence, successful MT-depletion was observed in 36% (n=105) of the resulting offspring of the genotype *UAS-Spastin (X)/+ or Y;+; fkhGal4/+*. The timing of *fkhGal4* expression, with expression starting only at the end of stage 10, is the likely reason that only 36% of placodes showed successful depletion.

To analyse myosin II localisation in MT-depleted placodes, virgins of *yw/+; sqh::sqhGFP42/+; sqh::sqhGFP40/fkhGal4* were crossed to males of *UAS-Spastin (X)/Y; sqh::sqhGFP42/+; sqh::sqhGFP40/+*. To induce degradation of *sqhGFP* specifically in the placode, males of *UAS-deGradFP/+; fkhGal4/+* were crossed to virgins of *sqh^{AX3}; sqh::sqhGFP42; GAP43mCherry*, leading to all male embryos being mutant for *sqh*. Embryos with *sqhGFP* targeted for degradation could be identified by the large aggregates that *sqhGFP* forms within the placode under these conditions (Caussinus et al., 2012). To affect endogenous Shot by expression of *UASp-GFP-Shot-EFGas2*, virgins of *nanosGal4VP16* (III) were crossed to males of

fkhGal4::UASp-GFP-Shot-EFGas2 (III), and the resulting offspring (F1) was backcrossed and the F2 embryos analysed. Note that expression of *UASp-GFP-Shot-EFGas2* by *fkhGal4* alone does not produce any dominant-negative effect (Maybeck and Röper, 2009).

In order to be able to compare equivalent developmental time points when salivary gland placode invagination was disrupted by expression of transgenes under *fkhGal4* control, we used the developmental state of the tracheal pits and their invagination as a timing control.

Embryo Immunofluorescence Labelling, Confocal, and Live Analysis

Embryos were fixed, stained and imaged using standard procedures, for details please refer to the Supplemental Experimental Procedures.

Embryos were collected on grape- or apple juice-juice plates and processed for immunofluorescence using standard procedures. Briefly, embryos were dechorionated in 50% bleach, fixed in 4% formaldehyde, and stained with phalloidin or primary and secondary antibodies in PBT (PBS plus 0.5% bovine serum albumin and 0.3% Triton X-100). anti-Crumbs and anti-E-Cadherin antibodies were obtained from the Developmental Studies Hybridoma Bank at the University of Iowa; the anti-dCrebA antibody was a kind gift from Deborah Andrew (Andrew et al., 1997); the anti Shot spectrin repeats was made in our lab (Röper and Brown, 2003); anti tyrosinated α-tubulin (YL1/2; Millipore); anti acetylated α-tubulin (clone 6-11B-1; Sigma); anti γ-tubulin (clone GTU-88; Sigma); anti asterless was a kind gift from Jordan Raff (Stevens et al., 2009). Secondary antibodies used were Alexa Fluor 488/Fluor 549/Fluor 649 coupled (Molecular Probes) and Cy3 and Cy5 coupled (Jackson ImmunoResearch Laboratories), and rhodamine-phalloidin was from Molecular Probes. Samples were embedded in Citifluor (Citifluor Ltd.) or Vectashield (Vectorlabs).

Images of fixed samples were acquired on an Olympus FluoView 1000/1200 or a Zeiss 780 Confocal Laser scanning system as z-stacks to cover the whole apical surface of cells in the placode. Z-stack projections were assembled in ImageJ or Imaris (Bitplane), 3D rendering was performed in Imaris.

For live time-lapse analysis of *sqh^{AX3}; sqh::sqhGFP42; GAP43-mCherry* embryos (alone or in combination with *fkhGal4 UAS-Spastin*), of embryos of the genotype *sqh^{AX3}; sqh::sqhGFP42/deGradFP; fkhGal4/GAP43-mCherry*, and of embryos of the genotype *GFP-Clip170/ECad-Tomato; fkhGal4*, the embryos were dechorionated in bleach, rinsed in water and attached to a coverslip with the ventral side up using heptane glue and covered with Halocarbon Oil 270 (saturated with water). Embryos harbouring *fkhGal4 UAS-Spastin* were identified by the loss of apical constriction due to MT-depletion as identified in the static analysis. Time-lapse sequences were acquired on a Zeiss 780 Laser scanning system as z-stacks. Z-stack projections to generate movies were assembled in ImageJ or Imaris.

Quantification of colocalisation

<u>*Microtubules versus myosin, centrosomes or Shot:*</u> Overlap of signal of either MT-labeling and medial myosin, MT-labeling and centrosomes, or MT-labeling and Shot localisation within the placode was counted in ImageJ using the 'Cell Counter' plugin (Kurt De Vos, Institute for Translational Neuroscience, University of Sheffield), using either images of fixed *sqh^{AX3};sqhGFP42* and *yw;sqhGFP42;sqhGFP40* stained for acetylated α-tubulin or *fkhGal4 x srcGFP* embryos stained for acetylated α-tubulin and asterless, or acetylated α-tubulin and Shot. The placode area was identified from the position of the circumferential myosin cable (Röper, 2012) or the extent of *srcGFP* expression. Colocalisation was confirmed by visually scanning through the apical-most 2 μm (5 confocal sections at 0.5 μm apart).

Fluorescence intensity quantifications

Non-centrosomal γ-tubulin: Images were taken of salivary gland placodes (marked with dCrebA or *fkh-Gal4::srcGFP*) and surrounding tissue at early stage 11 and at late stage 11. Any γ -tubulin signal that colocalised with centrosomes (marked with anti-asterless antibody) was masked using Imaris software (Bitplane). Maximum intensity projections of the apical surface of placodal cells were generated using 5 optical section separated by 0.5 µm each in z. For each embryos analysed, fluorescence measurements were made in a 10x10 µm box inside the placode (three measurements) and in the surrounding tissue (four measurements). The background fluorescence was determined by taking three fluorescence intensity measurements in areas of varying sizes outside the embryo. Corrected total cell fluorescence (CTCF) for γ -tubulin inside and outside the placode was calculated as follows:

CTCF = IntegratedDensity – (area x mean background fluorescence).

Mean fluorescence intensity per µm was calculated by dividing the sum of the CTCF by the sum of area measurements, and the ratio of [mean fluorescence intensity – inside placode]/[mean fluorescence intensity- outside placode] was generated for each projection. Six embryos were analysed at both stages.

Medial versus junctional myosin II, actin and Shot in control and MT-depleted placodes, or control and GFP-Shot-EFGas2-expressing placodes :

Images were taken of salivary gland placodes (identified by the surrounding myosin cable or by *srcGFP*) at late stage 11.

Fluorescence intensity per μm^2 was determined in ImageJ. Measurements were taken for the junctional pool and medial pool of myosin (using *sqhGFP*), actin (using *utrophinGFP*) or Shot from ≥10 cells outside the placode (i.e. outside the circumferential placodal actomyosin cable), giving a mean value for extraplacodal junctional actin/myosin/Shot and extraplacodal medial actin/myosin/Shot. Cells were then randomly selected from the placode (within the circumferential placodal

actomyosin cable; those selected in MT depleted placodes were also selected to be completely MT free; numbers of cells and placodes are listed in Table 1) and fluorescence intensity per μm^2 was determined for junctional and medial actin/myosin/Shot. A 7 pixel wide (0.728 μm) line was drawn around the junction (marked by DE-Cadherin staining) and used to measure the intensity of fluorescence at junctions. A second reading was taken over the corresponding medial area (now marked to be inside the 7 pixel line) to measure medial fluorescence.

For each placodal cell, the extraplacodal junctional and extraplacodal medial means were subtracted from the placodal junctional and medial measurements, respectively, to show how much the placodal junctional and medial fluorescence was raised above the mean junctional and mean medial fluorescence of the surrounding tissue.

Supplemental References

Andrew, D.J., Baig, A., Bhanot, P., Smolik, S.M., and Henderson, K.D. (1997). The Drosophila dCREB-A gene is required for dorsal/ventral patterning of the larval cuticle. Development *124*, 181-193.

Caussinus, E., Kanca, O., and Affolter, M. (2012). Fluorescent fusion protein knockout mediated by anti-GFP nanobody. Nature Struct. & Mol. Biol. *19*, 117-121.

Henderson, K.D., and Andrew, D.J. (2000). Regulation and function of Scr, exd, and hth in the Drosophila salivary gland. Dev. Biol. *217*, 362-374.

Martin, A.C., Kaschube, M., and Wieschaus, E.F. (2009). Pulsed contractions of an actin-myosin network drive apical constriction. Nature *457*, 495-499.

Maybeck, V., and Röper, K. (2009). A targeted gain-of-function screen identifies genes affecting salivary gland morphogenesis/tubulogenesis in Drosophila. Genetics *181*, 543-565.

Rauzi, M., Lenne, P.F., and Lecuit, T. (2010). Planar polarized actomyosin contractile flows control epithelial junction remodelling. Nature *468*, 1110-1114.

Röper, K. (2012). Anisotropy of Crumbs and aPKC Drives Myosin Cable Assembly during Tube Formation. Dev. Cell *23*, 939-953.

Röper, K., and Brown, N.H. (2003). Maintaining epithelial integrity: a function for gigantic spectraplakin isoforms in adherens junctions. J. Cell Biol. *162*, 1305-1315.

Royou, A., Field, C., Sisson, J.C., Sullivan, W., and Karess, R. (2004). Reassessing the role and dynamics of nonmuscle myosin II during furrow formation in early Drosophila embryos. Mol. Biol. Cell *15*, 838-850.

Sherwood, N.T., Sun, Q., Xue, M., Zhang, B., and Zinn, K. (2004). Drosophila spastin regulates synaptic microtubule networks and is required for normal motor function. PLoS Biol. *2*, e429.

Stevens, N.R., Dobbelaere, J., Wainman, A., Gergely, F., and Raff, J.W. (2009). Ana3 is a conserved protein required for the structural integrity of centrioles and basal bodies. J. Cell Biol. *187*, 355-363.

Stramer, B., Moreira, S., Millard, T., Evans, I., Huang, C.Y., Sabet, O., Milner, M., Dunn, G., Martin, P., and Wood, W. (2010). Clasp-mediated microtubule bundling regulates persistent motility and contact repulsion in Drosophila macrophages in vivo. J. Cell Biol. *189*, 681-689.

Zhou, B., Bagri, A., and Beckendorf, S.K. (2001). Salivary gland determination in Drosophila: a salivary-specific, fork head enhancer integrates spatial pattern and allows fork head autoregulation. Dev. Biol. *237*, 54-67.
